# *Insplico*: effective computational tool for studying splicing order of adjacent introns genome-wide with short and long RNA-seq reads

**DOI:** 10.1093/nar/gkad244

**Published:** 2023-04-07

**Authors:** André Gohr, Luis P Iñiguez, Antonio Torres-Méndez, Sophie Bonnal, Manuel Irimia

**Affiliations:** Centre for Genomic Regulation (CRG), The Barcelona Institute of Science and Technology, Barcelona, Spain; Centre for Genomic Regulation (CRG), The Barcelona Institute of Science and Technology, Barcelona, Spain; Centre for Genomic Regulation (CRG), The Barcelona Institute of Science and Technology, Barcelona, Spain; Centre for Genomic Regulation (CRG), The Barcelona Institute of Science and Technology, Barcelona, Spain; Centre for Genomic Regulation (CRG), The Barcelona Institute of Science and Technology, Barcelona, Spain; Universitat Pompeu Fabra (UPF), Barcelona, Spain; ICREA, Barcelona, Spain

## Abstract

Although splicing occurs largely co-transcriptionally, the order by which introns are removed does not necessarily follow the order in which they are transcribed. Whereas several genomic features are known to influence whether or not an intron is spliced before its downstream neighbor, multiple questions related to adjacent introns' splicing order (AISO) remain unanswered. Here, we present *Insplico*, the first standalone software for quantifying AISO that works with both short and long read sequencing technologies. We first demonstrate its applicability and effectiveness using simulated reads and by recapitulating previously reported AISO patterns, which unveiled overlooked biases associated with long read sequencing. We next show that AISO around individual exons is remarkably constant across cell and tissue types and even upon major spliceosomal disruption, and it is evolutionarily conserved between human and mouse brains. We also establish a set of universal features associated with AISO patterns across various animal and plant species. Finally, we used *Insplico* to investigate AISO in the context of tissue-specific exons, particularly focusing on SRRM4-dependent microexons. We found that the majority of such microexons have non-canonical AISO, in which the downstream intron is spliced first, and we suggest two potential modes of SRRM4 regulation of microexons related to their AISO and various splicing-related features. *Insplico* is available on gitlab.com/aghr/insplico.

## INTRODUCTION

Precursor mRNA (pre-mRNA) splicing is the processing step in the gene expression pathway that involves the removal of intronic sequences and ligation of exonic sequences to form mature RNAs (mRNAs). This process is carried out by the spliceosome, a complex molecular machinery that needs to be re-assembled *de novo* for every splicing reaction. By differentially selecting competing splice sites in each pre-mRNA molecule, the spliceosome can give rise to multiple mRNA isoforms per gene, a process referred to as alternative splicing. These alternative choices of splice sites are determined by intronic and exonic *cis*-acting elements and auxiliary proteins known as RNA binding proteins (RBPs). Some *trans*-acting factors have a tissue-specific expression and, therefore, contribute to the establishment of splicing regulatory networks responsible for cell specialization, particularly in the nervous system (1). The different steps of the gene expression pathway, including transcription itself, are interconnected and can form additional layers of (alternative) splicing regulation (2–4).

It is now well established in different species that the vast majority of genes undergo co-transcriptional splicing, i.e. splicing takes place as transcription is still occurring or shortly after (5–8). However, this does not necessarily imply that splicing always follows the order in which introns are transcribed, as initially thought (9). Single-gene studies as well as more recent genome-wide analyses have confirmed that intron splicing order does not always occur linearly following the order of transcription (10–16). In fact, when considering the average internal exon as reference, the downstream intron is spliced before the upstream one nearly as often as the opposite (15). In this study, we refer to the relative order of splicing of the two introns around an internal exon of interest as Adjacent Introns' Splicing Order (AISO).

Considering that early spliceosomal components are recruited at the time of transcription, multiple variables may influence AISO. As in alternative splicing regulation, both *cis*-acting elements – including strength of the splice sites and polypyrimidine tract, distance from branch point to the 3′ splice site (3′ ss), and other genomic features such as the size of the introns/exons and GC content (15)—and *trans*-acting factors (15), as well transcription kinetics (17–19) have been reported to affect AISO. Importantly, it is known that AISO can impact splicing decisions. For instance, mutations in an acceptor splice site in the *COL5A1* gene leads to changes in AISO, which affect the inclusion rate of the neighboring exons (20). Moreover, the exon junction complex (EJC), deposited upon splicing completion, has been shown to impact the splice site selection in subsequent splicing events (21,22), providing one possible mechanism by which AISO can modulate splicing decisions. However, the converse, i.e. whether (alternative) splicing regulation across tissues or conditions affects AISO, remains unknown. Similarly, how alternative splicing regulation through specific *trans*-acting factors relates to AISO is poorly understood.

To investigate AISO across different cell and tissue types as well as regulatory conditions, and to facilitate further research on this topic, we developed *Insplico*, the first standalone software to investigate AISO applicable to both short and long RNA sequencing reads. We demonstrated its robustness and effectiveness using simulated RNA-seq data and by comparing it to previous studies on AISO (15,16), which recapitulated previous findings and revealed unappreciated biases introduced by long read sequencing. Next, we compared AISO in different cell and tissue types from multiple species (mammals, non-vertebrate model organisms and plants), identifying universal genomic features associated with different modes of AISO and showing that AISO is highly constant across cell and tissue types within a given species. Finally, we explored AISO for introns flanking microexons whose inclusion is dependent on SRRM4 expression, and found two subsets of microexons with opposite AISO patterns and seemingly distinct features, suggesting two distinct modes of regulation.

## MATERIALS AND METHODS

### *Insplico*: input data generation and exon definition

*Insplico* takes as input mapped RNA-seq reads in BAM format. These must have been aligned with a splice-aware mapper, e.g. STAR or HISAT2 for short reads, or with Minimap2 for ONT/PacBio long reads. Furthermore, *Insplico* needs a tab-separated table defining the exons for which read statistics are desired. Exons are defined by their start and end coordinates, strand, and the end/start coordinate(s) of their upstream/downstream neighbor exons, respectively. More details on this exon-defining table can be found on gitlab.com/aghr/insplico. In addition, a script (*extract_exons_from_gtf.pl*) that allows users to create such a suitable table from a gene annotation file in GTF format is provided. This script clusters overlapping exons from several transcripts of the same gene into complex exon entities with potentially several start and end coordinates (following the logic of *vast-tools* (23)) and it implements a heuristic to identify intron-retention events that are not considered true exons. In addition, it identifies and assigns an exon type to each exon (Figure 3), e.g. *sfrst* (second first), if the exon is the second-first exon in all transcripts where it appears, *slst* (second last), if it is always the second-last exon, or *diverse* if it appears in different positions in different transcripts. From the exon table, *Insplico* extracts for each exon the set of start coordinates, the set of end coordinates, the strand, and the upstream and downstream intron regions. These regions are defined by the intronic region between the exon and its direct upstream and downstream neighbor exons while considering the maximal extent of these exons. Importantly, to reduce potential biases stemming from upstream and downstream introns of different lengths, *Insplico* utilizes as effective length of both introns the length of the shorter intron. For instance, if the upstream intron is 500 nts and the downstream one is 3000 nts, only the neighboring 500 nts for both introns are considered by *Insplico*. This approach effectively results in no biases associated with F*upfi* quantifications for different intron lengths, as shown by simulated RNA-seq data (Figure 2F, G).

### *Insplico*: algorithmic details

**Figure 1.**
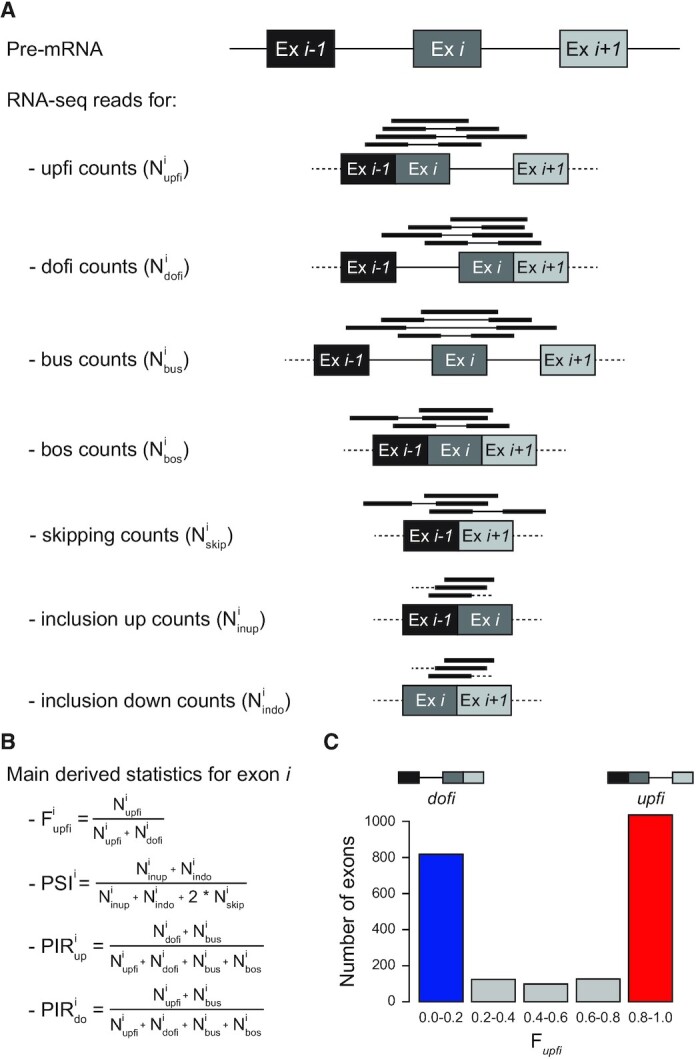
Summary of AISO and splicing related statistics provided by *Insplico*. (**A**) Schematic representation of mapped short reads that are informative for each type of processing state for a specific exon (Ex *i*). These include counts for exons in which either the upstream or downstream intron has been spliced first (*upfi* and *dofi*, respectively), and for which none or both of the adjacent introns have been spliced (*bus* and *bos*, respectively). It also includes exon-exon junction counts, for skipping or inclusion, used to derive exon inclusion levels (*skip*, *inup* and *indo*). Thin lines in mapped reads represent the non-sequenced fragment of paired-end reads. (**B**) Main statistics used in this study, as provided by *Insplico*. (**C**) Histogram showing the distribution of a representative set of exons based on their F*upfi* values. Throughout the study, exons with F*upfi* ≥0.8 (red) and ≤0.2 (blue) are considered *upfi* and *dofi* exons, respectively.

*Insplico* is implemented in Perl, uses exclusively Perl libraries shipped with the standard installation of Perl and depends on Samtools (24) and Bedtools. As such, it can be readily run after download on Unix-like systems where Perl, Samtools, and Bedtools are available. Considering all exon starts and ends and the upstream and downstream intronic regions of same length, *Insplico* inspects the mapped reads to identify those in *upfi*, *dofi*, *bos* and *bus* configurations (Figure 1), as well as the counts for exon skipping, inclusion upstream and inclusion downstream. These read count statistics, together with estimates of F*upfi* (fraction of *upfi* reads over the total number of *upfi* + *dofi* reads), proportion of exon inclusion (using the standard proportion-spliced-in metric, PSI) and proportion of intron retention (PIR) of the upstream and downstream introns, are output as a tab-separated table where rows (exons) are ordered correspondingly to the rows of the input table. Unavailable estimates are indicated by NA.

Three features of *Insplico*’s implementation facilitate its usability: (i) *Insplico* works out-of-the-box with mapped short and long reads. Algorithmically, long reads are treated identically to single-end short reads as there are no conceptual differences between these two for AISO analysis; (ii) users do not need to define the strandedness of the RNA-seq reads, which is often a cumbersome matter for incompletely documented RNA-seq data and (iii) users can use 0- or 1-based genetic coordinate systems without the need to change the reference system because *Insplico* uses a fuzzy matching of coordinates. When comparing the splice site coordinates of reads to exon start and end coordinates, *Insplico* can apply a user-definable fuzziness to detect matching coordinates; by default, coordinates are matched within ±3 nt. Fuzzy matching of coordinates is useful when ambiguities in splice site mapping occur where *Insplico* is capable of exploiting such reads for its count statistics.

Another capability that sets *Insplico* apart from previous approaches is the bias correction for processing long reads such as those from ONT or PacBio. When these reads do not cover the entire transcript, from transcription start site to termination site, they might cause a bias in the *upfi* and *dofi* count statistics of the first and last internal exons covered by the reads. To reduce this bias, *Insplico* implements a simple but effective heuristic which can be activated by the user (see ‘Long read bias correction by *Insplico*’ for more details). Further details on *Insplico*, available command arguments and applications can be found on gitlab.com/aghr/insplico.

### Simulation of RNA-seq data and assessment of detection and quantification biases

**Figure 2.**
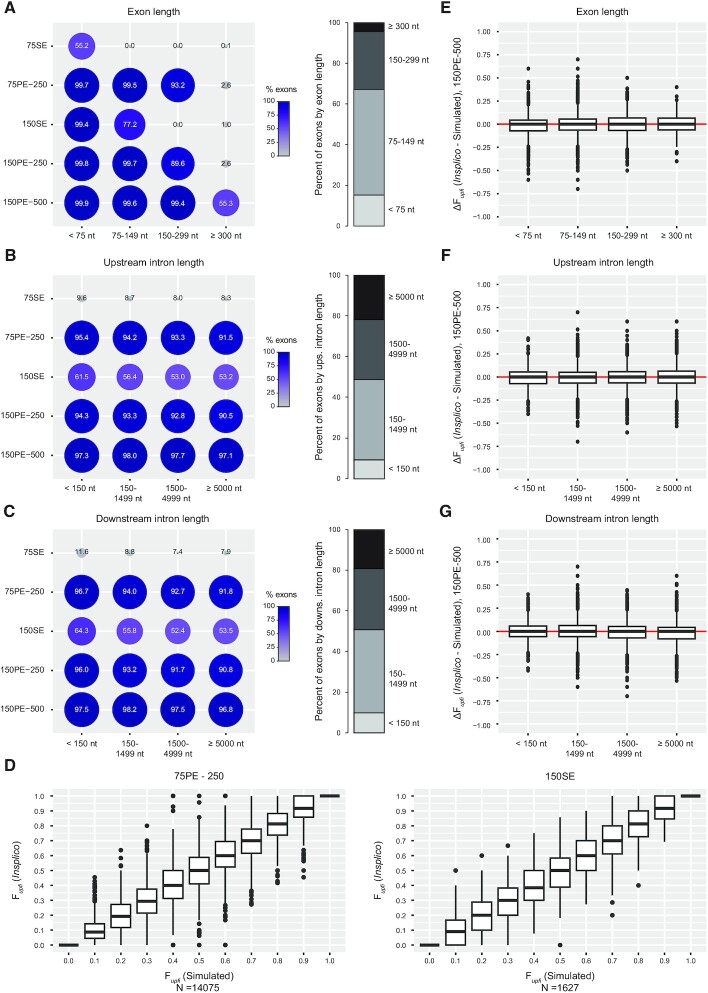
Assessment of potential *Insplico* biases using simulated reads. (**A–C**) Right: Dot plots showing the percent of exons for which at least one *upfi* or *dofi* count could be extracted using *Insplico* for different ranges of exon length (A), upstream intron length (B) or downstream intron length (C), using simulated RNA-seq reads of different lengths (75 or 150 nts) that are either single-end (SE), or paired-end (PE) with two possible average insert sizes (250 or 500 nts). For example, 150PE-500 corresponds to 150-nt PE reads with an average insert size of 500 nts. Dot size and heatmap are relative to the percent of detected exons. Left: proportion of internal exons for each feature range in human. (**D**) Boxplots of F*upfi* values as quantified by *Insplico* (*Y* axes) for exons with N*(upfi + dofi)* ≥ 10 for each simulated median F*upfi* group (*X* axes) for 75PE-250 and 150SE reads. (**E–G**) Difference between the F*upfi* values calculated by *Insplico* and those expected from the simulations (*Y* axes) for each range of exon length (E), upstream intron length (F) or downstream intron length (G), for simulated 150PE-500 reads. Median values around the red line (*Y* = 0) imply no bias in *Insplico*’s quantifications. Different types of simulated reads yielded similar results.

To generate the mRNA fasta file (i.e. transcriptome) used as input for each iteration of simulation of RNA-seq reads, 1000 random internal exons were selected from the Ensembl hg38 assembly (version 85), using only transcripts with support level 1 and limiting the number of selected exons to one per isoform and only one isoform per gene. The isoform selected per gene was chosen randomly weighting it by the number of exons; this means that isoforms with more exons were more likely to be selected per gene. Additionally, genes with an elevated number of exons had higher chances of being selected. For each gene we generated between 10 to 20 mRNA molecules with either the upstream intron or the downstream intron of the selected exon being ‘retained’ (i.e. not yet spliced out), whereas the rest of introns from the selected transcript were spliced out. The proportion of molecules with the upstream versus downstream retained intron (simulated F*upfi* value) was generated randomly using 0.1 intervals from 0 to 1 at equal probability (uniformly distributed). For each of these exons, we then extracted the internal exon length, upstream intron length and downstream intron length, as well as simulated F*upfi* value. We used the generated mRNA fasta file to simulate RNA-seq reads of different lengths (75 and 150 nts) that were either single-end or paired-end with different average insert sizes (250 or 500 nts, from the beginning of the forward read to the end of the reverse read) were generated employing the *simulate_experiment_countmat* function from the bioconductor R package *polyester* v.1.34.0 with the error model of ‘illumina5’ for 75 bp reads and ‘uniform’ for 150 bp and the options: strand_specific = F, error_rate = 0.005, bias = ‘none’. This process was repeated 20 times (*i* = 1...20 iterations) to generate data for a total of 20 000 internal exons, using seed = 20221107 + i to ensure reproducibility. Next, each generated RNA-seq file was mapped with STAR and processed with *Insplico*. For the different analyses of Figure 2, simulated exons were grouped according to their length or the length of their upstream or downstream introns. For detection assessment, we plotted the percent of simulated exons for which *Insplico* could extract at least one *upfi* or *dofi* read (i.e. N(*upfi* + *dofi*) ≥ 1). For quantification assessments, we restricted the analyses to exons with N(*upfi* + *dofi*) ≥ 10. Scripts for reproducing simulated data can be found at https://github.com/liniguez/Insplico_simulations.

### Comparative re-analysis of published data (Kim *et al.*, 2017)

**Figure 3.**
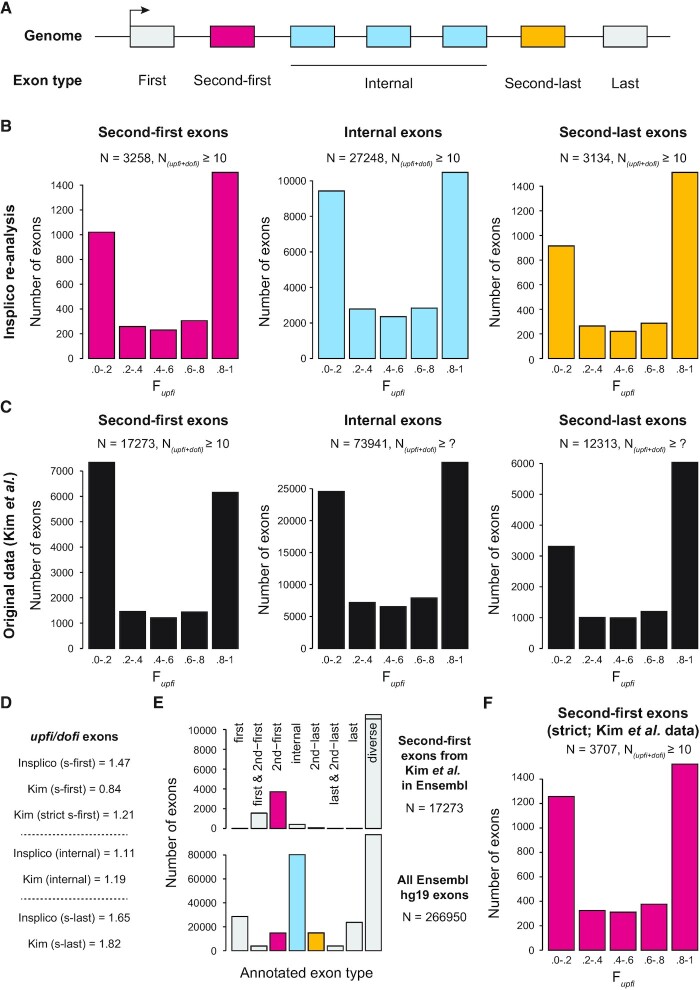
Reproduction of major AISO patterns from Kim *et al.* (2017) using *Insplico*. (**A**) Schematic representation of the different groups of exons investigated for a representative gene. (**B**) Distribution of exons according to F*upfi* values generated by *Insplico* for second-first (left), truly internal (middle) and second-last (right) exons as defined by *extract_exons_from_gtf.pl*. (**C**) Distribution of exons according to F*upfi* values for second-first (left), truly internal (middle) and second-last (right) exons as provided by Kim *et al.* N(*upfi + dofi)* for internal and second-last exons was not provided in the original publication. (**D**) Distribution of second-first exons as annotated by Kim *et al.* according to exon types extracted by *extract_exons_from_gtf.pl*. ‘Diverse’ correspond to exons with more than one type of annotation across transcripts. (**E**) Distribution of exon types as extracted by *extract_exons_from_gtf.pl* for all exons in the Ensembl hg19 annotation. (**F**) Distribution of strictly defined second-first exons from Kim *et al.*’s original set according to F*upfi* values as provided by Kim *et al.* (15).

GEO IDs of all 57 RNA-seq data sets used by Kim *et al.* (15) from total or nuclear RNA without poly-selection are listed in Supplementary Table S1. After download, we removed the Illumina universal adapter AGATCGGAAGAGC with cutadapt v2.4 from the 3′ ends of read1 and read2, keeping only reads with a minimum length of 15 nts. As per the original study, these reads were mapped to the human hg19 genome assembly with the splice-aware mapper STAR v2.7.1a, requiring a minimal overlap of 5 nts on both sides of splice junctions and keeping only uniquely mapping reads. The resulting BAM files with mapped reads were analyzed with *Insplico*. To create the exon definition table, we applied the script *extract_exons_from_gtf.pl* to the Ensembl v75 hg19 gene annotation GTF, which gave 266950 exons together with their exon types across all transcripts where they appear. The most prominent exon types were diverse/mixed (39.2%), internal (30.1%), first (10.7%) and last (8.9%), as shown in Figure 3E. We discarded exons of the ‘diverse’ type as they may introduce noise, and focused on second-first, internal and second-last exons. Only exons with a minimum of ten *upfi* + *dofi* reads (*N*(*upfi + dofi)* ≥ 10) were included in the analysis. To directly compare these results with those published by Kim *et al.*, we downloaded the tables with their results as provided on http://fairbrother.biomed.brown.edu/data/Order. The table first_introns_splicing_pair_counts.txt contained a list of 43 547 second-first exons as identified by Kim *et al.* with F*upfi* values, of which 17 273 had *N*(*upfi + dofi*) ≥ 10. The tables middle_intron_scores.txt and last_intron_scores.txt contained 73941 internal and 12313 second-last exons, respectively, all of which were plotted since N(*upfi + dofi)* was not provided. Finally, to investigate the cause of the difference in AISO profiles obtained by our and Kim *et al.*'s analysis for second-first exons, we matched the 17 273 second-first exons identified by Kim *et al.* to the exons types we extracted from the Ensembl hg19 gene annotation, which revealed that most of these exons were of the ‘diverse’ type.

### Comparative re-analysis of published ONT long read data (Drexler *et al.*, 2020)

Drexler *et al.* (16) sequenced chromatin, 4sU enriched RNA from K562 cells using ONT. We downloaded this dataset and mapped it to the human genome hg38 assembly with the *Minimap2* v2.17-r974-dirty (25) in splice mode with seeds of length 14 nts. To create the exon input table, we applied the script *extract_exons_from_gtf.pl* to the gene annotation GTF from Ensembl v88, together with all exons from *VastDB* (23), obtaining a total of 233 306 unique exons. The BAM file from *Minimap2* and the exon table were used to run *Insplico* in standard mode to extract raw read count statistics. A histogram of F*upfi* values was plotted for all exons with N(*upfi +*d*ofi*) ≥ 4. In addition, we downloaded Illumina paired-end short reads for chromatin, 4sU-enriched RNA also generated by Drexler *et al.* (Supplementary Table S1). We removed the Illumina universal adapter AGATCGGAAGAGC with cutadapt v2.4 from the 3′ ends of read1 and read2, and kept reads with a minimum length of 15 nts. These reads were mapped to the same human hg38 assembly with STAR v2.7.1a, requiring a minimal overlap of 5 nts on both sides of splice junctions and keeping only uniquely mapping reads. We then ran *Insplico* on the resulting BAM files with the same exon input table used for the analysis of ONT long reads and generated F*upfi* histograms for all exons with N(*upfi +*d*ofi*) ≥ 10.

### Long read bias correction by *Insplico*

**Figure 4.**
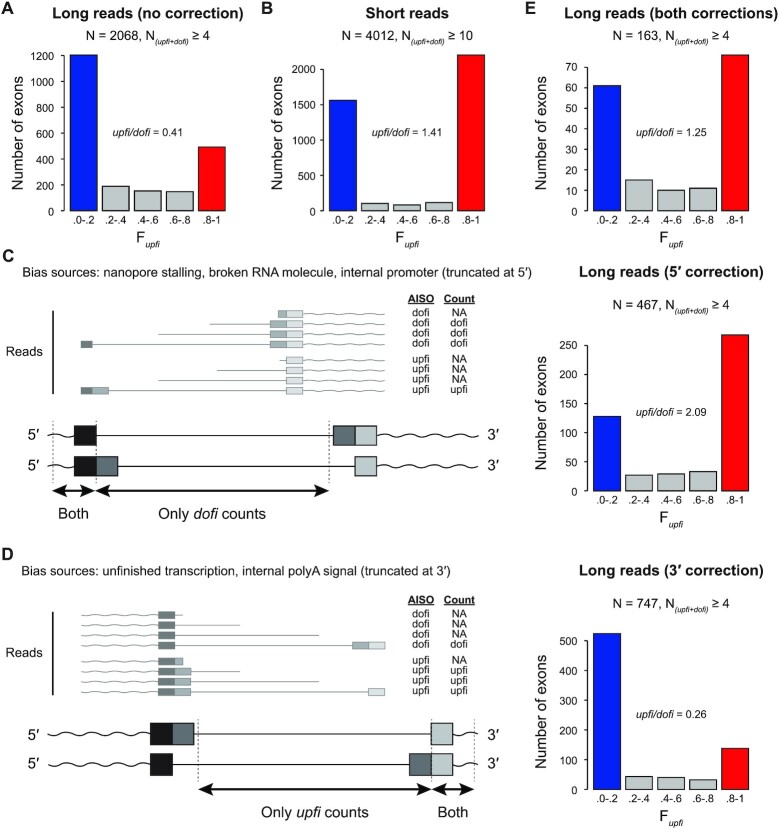
Re-analysis of AISO patterns using Drexler *et al.* (2020) ONT data. (**A**) Distribution of internal exons according to F*upfi* values generated by *Insplico* using ONT reads from Drexler *et al.* (16) without correction reproduces the excess of *dofi* exons (blue) reported by the original study. (**B**) Distribution of internal exons according to F*upfi* values generated by *Insplico* using Illumina short reads for the same cell type as in (A). (**C**, **D**) Left: Schemes depicting how ONT reads can be truncated at their 5′ (C) or 3′ (D) ends, which is predicted to bias the total read count against *upfi* and *dofi* RNA molecules, respectively. Schemes depict the focus internal exon (dark grey) and the upstream (black) and downstream (light grey) exons, which are separated by equally long neighboring introns in this example. Wavy lines correspond to other parts of the RNA molecule. ONT read examples are shown smaller and with transparent coloring. The table indicates, for each depicted ONT read, the real AISO pattern (‘AISO’) and the count obtained by *Insplico* (‘Count’). Right: impact of corrections done by *Insplico* for each these biases in the AISO profiles from (A). (**E**) Distribution of internal exons according to F*upfi* values generated by *Insplico* using ONT reads from Drexler *et al.* (16) with full correction more closely reproduces the AISO profile obtained by short reads.

Given the different profiles obtained for short and long reads in the re-analysis of Drexler *et al.* (2020), we hypothesized that these differences could be caused by specific biases introduced by ONT. Specifically, we reasoned that there could be two types of biases: those resulting from 5′ truncations of the RNA molecule or sequence (Figure 4C) and those from 3′ truncations (Figure 4D). Each of these can come from different sources. For instance, since ONT sequencing proceeds from 3′ to 5′, 5′ truncations can be caused by broken RNA molecules or by molecule stalling at the nanopore, leaving the sequencing incomplete. In the case of 3′ truncations, these can occur when transcription of the RNA has not finished, as it often occurs in datasets of nascent RNA. Since the probability of these biases to occur generally depend on the length of the RNA molecule, and *Insplico* needs both ends of the target exon to be present in the sequence to provide a valid count, this can lead to a relative depletion of *upfi* and *dofi* counts for 5′ and 3′ truncations, respectively, as exemplified in Figure 4C, D for an exon with two neighboring introns of equal length.

To mitigate each of these types of bias, we implemented a simple heuristic in *Insplico* that users can activate with the option --biascorr. With this option activated, for each ONT read processed, *Insplico* identifies the first and last exons covered by the read and discards them for further processing as well as the sequence upstream or downstream to them, respectively. In other words, each ONT read will be cut at the first and last detected exons, which will account for 5′ and 3′ truncations, respectively. This implies that no *upfi* or *dofi* counts will be extracted for these discarded exons but, also, that in most cases no *upfi* or *dofi* counts will be extracted for the neighboring second-first and second-last exons from that read either. Therefore, with this heuristic activated, *Insplico* extracts *upfi* and *dofi* counts only for the subsequent internal exons covered by the read. It should be mentioned that, while this heuristic reduces global biases on AISO, it comes at the price of considerably reducing the number of extracted *upfi* and *dofi* counts. Moreover, as long read technologies and protocols improve, we expect fewer biases associated with AISO. Therefore, we opted for not having this correction active by default, but to leave its use to the user's discretion.

### Consistency of AISO across different replicates, tissues, experimental conditions and species

For studying AISO across different replicates, cell types, tissues and experimental conditions, we first obtained exon input tables for human hg38 and mouse mm10 Ensembl v88 annotations enriched with *VastDB* exons, as described above. We then downloaded RNA-seq datasets from various sources (Supplementary Table S1), removed the Illumina universal adapter AGATCGGAAGAGC with *cutadapt* v2.4 from the 3′ ends of read1 and read2, kept the reads with length ≥15 nts, and mapped them to the respective genomes using STAR v2.7.1a requiring a minimal overlap of 5 nts on both sides of splice junctions and keeping only uniquely mapping reads. These BAM files were used to run *Insplico*. In addition, for all datasets we also mapped and analyzed with *Insplico* the associated polyA-selected and/or cytoplasmic RNA samples, and utilized the estimated PIR values to filter out exons associated with retained introns (PIR ≤ 0.15 for the upstream or downstream intron), and only truly internal exons (as defined above) were used for these comparisons. Then, to compare AISO patterns between two conditions (biological replicates, different cell types, different species), we first selected exons with *N*(*upfi + dofi)* ≥ 10 in both conditions. Next, we defined *upfi*, *dofi* and intermediate exons in the query condition (first stack plot) as those with F*upfi* ≥ 0.8, F*upfi* ≤ 0.2 and 0.2 > F*upfi* > 0.8, respectively, and investigated what fraction of each of them was *upfi, dofi* or had intermediate F*upfi* values in the target condition (second to fourth stack plots). As explained in Supplementary Figure S2A, if AISO is similar in both conditions (‘Consistent’), most *upfi* exons of the query condition will also be *upfi* in the target condition (and the same for *dofi* exons). On the other hand, if AISO patterns are not maintained between conditions (‘Random’), the *upfi* and *dofi* exon sets in the query condition should have an AISO pattern similar to the genome-wide pattern in the target condition.

### Universal features associated with AISO patterns

To investigate which intron-exon related features affected AISO genome-wide across multiple species, we first selected 13 RNA-seq datasets from different species (Supplementary Table S1) and processed them with *Insplico*. As a standard procedure, we removed the Illumina universal adapter AGATCGGAAGAGC with *cutadapt* v2.4 from the 3′ ends of read1 and read2, keeping only reads with length ≥15 nts. These reads were mapped to the corresponding genomes of each species with the splice-aware mapper STAR v2.7.1a requiring a minimal overlap of 5 nts on both sides of splice junctions and keeping only uniquely mapping reads. The STAR index was built considering the gene annotations for each species and we obtained the exon-definition table applying *extract_exons_from_gtf.pl* to each GTF file together with exons annotated in *vast-tools* (23), except for rice (not available). Specifically, we used the following species and genome versions: *Homo sapiens* (hg38, Ensembl v88), *M. musculus* (mm10, Ensembl v88), *D. melanogaster* (dm6, Ensembl Metazoa v26), *C. elegans* (ce11, Ensembl v87), *A. thaliana* (araTha10, Ensembl Plants v31) and *O. sativa* (IRGSP1, Ensembl Plants v48).

Next, we plotted the distribution of F*upfi* values for subsets of exons according to multiple intron-exon related features extracted using *Matt* (26). Specifically, we used the *Matt* command *cmpr_features*, and, for each studied feature, we split the exons into five subsets of equal size with increasing feature value. These plots allow us to study how F*upfi* distributions change for subsets of exons with different feature values. A summary of these plots for those features consistently and significantly associated with AISO across species is shown in Figure 6C for all datasets. All the results, together with further details (violin plots with F*upfi* distributions, axis values, sample sizes, etc.) can be found in Supplementary File 1. It should be noted that, although we summarize the results in Figure 6C as median values, the distributions of F*upfi* values are bimodal, as it can be observed in the violin plots of Supplementary File 1. For all datasets, we only considered exons with N*(upfi + dofi)* ≥ 10 and for exons with multiple start and/or end coordinates, we chose the version with the longest length. In addition, for those datasets with matched polyA/cytoplasmic RNA-seq (Supplementary Table S1 and Supplementary File 1), we used the *Insplico* information to discard those exons whose upstream and/or downstream introns had PIR > 0.1.

### Comparisons of AISO and genomic features for exons based on their tissue-specific regulation

We generated a barplot of F*upfi* values for mouse exons depending on their splicing pattern (Figure 7A) as determined from the cytoplasmic polyA-selected brain and liver data that we generated for this study (Supplementary Table S1). In particular, we defined the following groups: (i) constitutively spliced (CS): exons with PSI > 0.99 in both tissues; (ii) tissue-regulated (TR): exons with an absolute difference in PSI between brain and liver higher than 0.25; and (iii) alternatively spliced (AS): exons with 0.1 < PSI < 0.9 in liver and/or brain and that are not TR. Only exons with at least 20 reads contributing to the PSI estimates both in brain and in liver polyA-selected samples and at least N*(upfi + dofi)* ≥ 10 in the brain chromatin-associated RNA-seq sample were used for the analysis. Exons with multiple start and/or end coordinates were discarded. Exon-intron related features were retrieved using *Matt cmpr_exons* (26) and plotted as *Z*-score values (Figure 7A). *P*-values corresponded to Bonferroni-corrected *P*-values from Wilcoxon Rank-Sum tests with respect to the distribution of the CS exons. Full details of the comparisons and all statistical tests are reported in Supplementary File 2.

### Analysis of SRRM4-dependent microexons

To define SRRM4-dependent microexons, we processed with *Insplico* as described above two replicates of total (generated for this study, see below) and matched polyA-selected (from (27) and (28)) RNA-seq data from human HEK293 cells ectopically expressing GFP (control) or 3xFlag-tagged human SRRM4. The two replicates were pooled together to increase read depth. To obtain the exon-definition table, we clustered Ensembl v88 annotations for hg38 and VastDB exons with *extract_exons_from_gtf.pl*. We then defined two sets of microexons, defined here as exons of length ≤51 nts: (i) SRRM4-dependent microexons: exons with a ΔPSI (SRRM4 - control) > 0.15 and a PSI in control cells ≤0.2 in the polyA-selected samples; and (ii) control microexons: with |ΔPSI| <0.02 and 0.05 ≤ PSI ≤ 0.95 in at least one of the samples. For exons with multiple start and/or end coordinates, we chose the version with the shortest length. Only exons with N(*upfi + dofi*) ≥ 5 in the SRRM4 OE total RNA sample were used for AISO analyses. Exons with *upfi* and *dofi* patterns were defined as those with F*upfi* ≥0.8 and ≤0.2, respectively. Intron-exon related features were extracted using *Matt cmpr_exons* (26) and the full report is shown in Supplementary File 3. The RNA map showing the distribution of UGC motifs was generated using *Matt rna_maps* (26), using a sliding window of 25 nts and limits of 20 and 150 nts for the exonic and intronic regions, respectively.

### Validation of AISO patterns through RT-PCR assays

Flp-in-T-REx 293 cells expressing either GFP or 3xFlag-tagged human SRRM4 were induced with 1 ug/ml for 24 h (27). Total RNA was extracted using the Illustra RNAspin Mini Isolation kit (GE Healthcare). Reverse transcription was performed using oligo-dT and random hexamer primers with an *in-house* enzyme produced by the Protein Technologies Unit at CRG. PCRs were performed using GoTag DNA polymerase (Promega) and primers annealing either of the flanking exons to look at the pattern of splicing or exon-junction (EJ) overlapping and intronic (I) primers to investigate the order of intron splicing. Primers (5′ to 3′, Sense (S) and AntiSense (AS)) used: *ERC1_EJ1_S*: AGCTGAGTTGGAAAGTCTCACCTC;*ERC1_I2_AS*:TCCCCTCCTCTTTCCTCGTA;*ERC1_I1_S*:TGTGACTCCTTCCCTTCTCT;*ERC1_EJ2_AS*:TATTCTGGTCTTTCACTTGCCTTGAGGTG;*GRAMD1A_EJ1_S*:TCATCAGCATTGTGATCTGT;*GRAMD1A_I2_AS*:CCCATTGCAGAGGAGGAGAA;*GRAMD1A_I1_S*:CGTCCTGAGAGAGTGGAGAC;*GRAMD1A_EJ2_AS*:GAGGATGATAAGGCTCACAC;*KIF1B_EJ1_S*:CTTGGCCGAGGTGGATAACT*;KIF1B_I2_AS*:ACCCACAGACACACAATCCA;*KIF1B_I1_S*:ATGCTGTTGATTTGAGGGCC;*KIF1B_EJ2_AS*:TCTTCTTTTTACTCTTGCTA;*UGGT2_EJ1_S*:TTTCTCTTTGGGAAACTAAAACAAGGAA;*UGGT2_I2_AS*:GAGAACCACCCTGAGAGTCC;*UGGT2_I1_S*:GCCCCAAAGAAAAGAAAACGT;*UGGT2_EJ2_AS*:TCTAAGATCTGAATATATTTCTCATGCTATTCCTTG. Events were selected among those having ΔPSI (SRRM4-GFP) > 40 and N*(upfi + dofi)* ≥ 3 in both SRRM4 total and SRRM4 polyA-selected RNA-seq.

### Tissue dissociation and cellular fractionation

Female mice (6–7 weeks old, B6CBAF1) were injected intraperitoneally with 5 IU of pregnant mare serum gonadotropin (PMSG), followed by intraperitoneal injection of 5 IU of human chorionic gonadotropin (hCG) 47 h after. Females were mated after hCG injection and tissues collected 20 h post hCG injection. Mouse euthanasia was performed by cervical dislocation. All animal related protocols were carried out in accordance to the European Community Council Directive 2010/63/EU and approved by the local Ethics Committee for Animal Experiments (Comitè Ètic d’Experimentació Animal-Parc de Recerca Biomèdica de Barcelona, CEEA-PRBB, CEEA number 9086).

The tissues (liver, cerebellum and cortex) were collected post-mortem in cold PBS and rinsed to remove the excess of blood. The tissues were sliced into small pieces using a blade and resuspended in 40 ml of dissociation buffer (trypsin 0.05% (ThermoFisher); 0.02 units/ml dispase (Life Technologies); 0.025 mg/ml collagenase (Life Technologies); 18 units/ml DNAse I (Sigma)). Samples were incubated at 4°C head-over-tail overnight before being filtered through a 100 micron strainer (BD Biosciences) to remove the undissociated tissues. Cells pellets were obtained by centrifugation at 1000 rpm for 5 min at 4°C and washed once in 1 ml PBS. Pellets were resuspended in pre-chilled HMKE buffer (20 mM HEPES pH 7.2; 5 mM MgCl_2_; 10 mM KCl; 1 mM EDTA; 250 mM sucrose; 1× protease inhibitors cocktail (Roche); 200 ug/ml digitonin (Sigma)) described in (29); supplemented with 0.1% NP40. Samples were incubated for 10 min on ice and centrifuged at 500 g for 10 min at 4°C. The supernatant for each tissue was kept and saved as cytoplasmic fraction. The pellet, containing the nuclei, was washed in PBS supplemented with 1 mM DTT, centrifuged again and treated following the protocol described by (30). Briefly, pellets were resuspended in pre-chilled buffer 1 (20 mM Tris–HCl pH 7.9; 75 mM NaCl; 0.5 mM EDTA; 0.85 mM DTT; 1× protease inhibitors cocktail (Roche); 50% glycerol). An equal volume of pre-chilled buffer 2 (10 mM HEPES pH 7.6; 1 mM DTT; 7.5 mM MgCl_2_; 0.2 mM EDTA; 0.3 M NaCl; 1 M Urea; 1% NP40) was added and the samples were vortexed twice for 2 s before being incubated for 10 min on ice.

The samples were centrifuged at 15 000 g for 2 min at 4°C. The pellet (chromatin fraction) was resuspended in PBS. All fractions were treated with proteinase K for one hour at 65°C (an equal volume of Proteinase 2x buffer (200 mM Tris 7.5, 25 mM EDTA, 300 mM NaCl, 2% SDS) was added to each fraction supplemented with 2 mg/ml proteinase K (Roche Diagnostics). A phenol/chloroform extraction was performed followed by a chloroform extraction and ethanol precipitation. The nucleic acid pellets were resuspended in water and treated with DNAse (RQ1, Promega) for one hour at 37°C according to the manufacturer's instructions. Phenol/chloroform and chloroform extractions and ethanol precipitation were performed and the pellets were resuspended in water.

### Preparation of RNA-seq libraries and short read sequencing

Total RNA libraries were prepared using the TruSeq Stranded Total RNA Library Prep Kit with Ribo-Zero Human/Mouse/Rat Kit (Ref: RS-122-2201/2202, Illumina) according to the manufacturer's protocol. Briefly, from 11.7 to 100 ng of total RNA were used for ribosomal RNA depletion. Then, ribosomal depleted RNA was fragmented for 4.5 min at 94°C. The remaining steps of the library preparation were followed according to the manufacturer's instructions. Final libraries were analysed on an Agilent Technologies 2100 Bioanalyzer system using the Agilent DNA 1000 chip to estimate the quantity and validate the size distribution, and were then quantified by qPCR using the KAPA Library Quantification Kit KK4835 (Ref: 07960204001, Roche) prior to amplification with Illumina's cBot. PolyA-selected libraries were prepared using the TruSeq stranded mRNA Library Prep according to the manufacturer's protocol using from 25 to 200 ng of total RNA as starting material.

Both total (cerebellum, cortex, liver and HEK293) and polyA-selected (cerebellum, cortex and liver) RNA libraries were sequenced on an Illumina HiSeq 2500 machine to generate 125 nt paired-end reads. Read numbers and mapping statistics are provided in Supplementary Table S1, and all samples were submitted to Gene Expression Omnibus (GEO), under the ID GSE207459. For all analyses, cerebellum and cortex RNA-seq reads were pooled together to generate a single ‘brain’ sample.

## RESULTS

### Algorithm overview and definitions

*Insplico* works with both short single-end or paired-end reads (Illumina or Ion Torrent) and long reads (Oxford Nanopore Technology [ONT] or PacBio), using as input BAM files of mapped reads together with a set of user-specified exons with flanking introns. Since splicing takes place in the nucleus during or shortly after transcription, chromatin-associated or nuclear RNA, or at least total ribo-depleted RNA, should preferably be used for studying AISO. *Insplico* automatically detects the read type (single-end or paired-end and their strandedness), and extracts counts of fragments mapping locally to each exonic region in different configurations (Figure 1A). Specifically, for a given exon, fragments mapping to the junction of that exon with any other upstream exon and to the unspliced downstream intron are labelled as upstream-first (*upfi*). Conversely, downstream-first (*dofi*) counts represent fragments that map to the unspliced upstream intron and the junction joining the exon with any other downstream exon. Besides these two types, *Insplico* quantifies fragments that map to both-unspliced (*bus*) flanking introns, as well as fragments supporting fully processed mRNA, i.e. mapping to both the upstream and downstream splice junctions with any upstream or downstream exon (both-spliced, *bos*). Additionally, it extracts counts of fragments supporting exon inclusion (from upstream or downstream exon-exon junctions) or exon exclusion, containing a splice junction that skips the exon (Figure 1A). With these counts, *Insplico* estimates different measures for each input exon (Figure 1B). The most relevant for AISO analysis of a given exon is F*upfi*, the fraction of *upfi* fragments in relation to the sum of *upfi* and *dofi* fragments. A F*upfi* close to 1 implies that the AISO of this exon is predominantly *upfi*, while F*upfi* close to 0 means it is predominantly *dofi*. In this study, we summarize the distributions of F*upfi* values for sets of exons of interest by empirical histograms (Figure 1C), which can be complemented with violin or density plots of F*upfi* values. In addition, for each input exon, *Insplico* provides: proportion of exon inclusion (PSI) and proportion of intron retention (PIR) for both flanking introns. These measures can be used to identify alternative versus constitutive exons, or exons that are specifically (mis-)regulated in a given condition (see below). Moreover, by additionally utilizing matched cytoplasmic and/or polyA-selected RNA-seq, PIR values of flanking introns can be estimated and used to remove stably retained introns, which may bias the analysis of AISO.

### Assessment of potential biases in AISO quantification using simulated reads

To evaluate *Insplico*’s performance in terms of quantification precision and assess possible biases coming from exon and intron lengths both in terms of detection ability and F*upfi* quantification, we first performed a controlled study using simulated short RNA-seq reads. We simulated RNA-seq reads of different lengths (75 and 150 nts) that were either single-end or paired-end with different insert sizes (250 and 500 nts) for a total of 20 000 random internal human exons (see Methods). With regard to detection, as expected by design (Figure 1A), the capability of *Insplico* to extract count statistics for exons above a certain length is limited by the read length and the insert size, in the case of paired-end reads (Figure 2A). For instance, with 75-nt single-end reads *Insplico* could extract counts only for exons of length ≤ 75 nts, while exons > 300 nts were only partially detected by 150-nt paired-end reads with insert sizes of 500 nts (Figure 2A, left panel). It should be noted, however, that exons > 300 nts account for < 5% of all internal exons in human (Figure 2A, right panel). In the case of the neighboring introns, neither the length of the upstream intron nor of the downstream intron had a major impact on the capability of *Insplico* to extract counts (Figure 2B, C). With regard to the quantification, we found no major differences in F*upfi* accuracy or precision related to the type of RNA-seq used (Figure 2D). More importantly, we found that the lengths of neither the exon, the upstream intron nor the downstream intron introduced any biases in the distributions of F*upfi* values measured by *Insplico* when compared to the simulated ones (Figure 2E–G), strongly supporting the validity of *Insplico* to quantify and compare AISO across the genome.

### *Insplico* analyses of published datasets reproduce previous results

Next, we tested the effectiveness of *Insplico* by applying it to the publicly available RNA-seq datasets used in Kim *et al.* (15) (Figures 3 and Supplementary Figure S1A). We downloaded and mapped the same 57 human RNA-seq samples from GEO, summing up to 7.3 billion 72–76 nt paired-end reads for non-polyA-selected total or nuclear RNA fractions from 16 different human cell lines (Supplementary Table S1). We then extracted all exons that were consistently second-first (i.e. always the second exon in every transcript in which it is present), truly internal, or second-last (i.e. always the second to last exon in every transcript in which it is present) from the complete Ensembl hg19 gene annotation (Figure 3A; see Materials and Methods), and applied *Insplico* to the mapped reads to extract AISO count statistics for exons with at least 10 (*upfi* + *dofi*) counts (N(*upfi* + *dofi*) ≥ 10). In agreement with Kim *et al.*, we found that, genome-wide, the majority of truly internal exons had either a clear *upfi* or *dofi* AISO pattern (F*upfi* ≥ 0.8 or ≤ 0.2, respectively) (Figure 3B,C; center plots), with a slight excess of *upfi* exons (Figure 3D; ratio *upfi*/*dofi* = 1.11; *P* = 6e-14, one-sided Binomial test). Second-last exons showed a stronger enrichment of *upfi* splicing patterns (Figure 3D; ratio *upfi*/*dofi* = 1.65; *P* = 2e–34, one-sided Binomial test), again in agreement with Kim *et al.* (Figure 3B, C; right plots). However, for second-first exons, we found a similar excess of *upfi* AISO (Figure 3D; ratio *upfi*/*dofi* = 1.47; *P* = 3e–22, one-sided Binomial test), similar to the trend observed for second-last exons, while Kim *et al.* reported an excess of *dofi* AISO (Figure 3B, C; left plots). To clarify this discrepancy, we further investigated the set of second-first exons as defined by Kim *et al.* using our exon-type classification. Remarkably, only a minority of these exons were truly second-first in all the transcripts in which they appear; the majority fell in the ‘diverse’ exon category (Figure 3E), which was the most common one in the annotation (Figure 3E). Restricting the analysis to the subset of exons used by Kim *et al.* that were strictly annotated as second-first by our annotation in combination with the F*upfi* values estimated by Kim *et al.* gave a pattern of AISO more similar to that obtained with *Insplico*, with an excess of *upfi* AISO (Figure 3D, F; ratio *upfi*/*dofi* = 1.21; *P* = 3e–7, one-sided Binomial test).

### Correction of long read biases recovers short read splicing patterns

We next used RNA-seq data of nascent chromatin-associated RNA from human K562 cells published by Drexler *et al.* (16) (Figures 4 and Supplementary Figure S1B). These data consist of ∼2 million long ONT reads, which we mapped to the human genome with *Minimap2*. Applying *Insplico* to these mapped reads showed an excess of *dofi* AISO (Figure 4A; ratio *upfi*/*dofi* = 0.41; *P* = 7e–69, one-sided Binomial test), in agreement with Drexler *et al.* (16). However, this pattern disagrees with the slight excess of *upfi* AISO described above obtained using short reads (Figure 3; (15)). To investigate potential causes of this discrepancy, we processed with *Insplico* ∼160 million 80-nt paired-end Illumina reads from 4sU-labelled RNA also from K562 cells generated by Drexler *et al.* We observed the same excess of *upfi* AISO (Figure 4B; ratio *upfi*/*dofi* = 1.41; *P* = 7e–26, one-sided Binomial test), suggesting that the nature of the sequencing data (long versus short read sequencing) may have a considerable impact on the results. We reasoned that this discrepancy could be explained by biases introduced by long reads. On the one hand, since ONT sequences the RNA molecule from 3′ to 5′, for broken molecules or those eventually stalled at the nanopore, the 5′-most exon identified in the read could have a bias towards *dofi* counts, since its upstream exon will often not be present and thus cannot produce *upfi* counts by definition (5′ truncations; Figure 4C). On the other hand, partially transcribed pre-mRNAs could generate a bias towards *upfi* splicing for the 3′-most exon identified in the sequenced molecule, since the upstream exon will already be transcribed (and thus potentially spliced to the focus exon) but not the downstream one (3′ truncations; Figure 4D). To reduce these potential biases, we added to *Insplico* an optional correction module for long reads. Effectively, the implemented strategy discards both the first and last exons identified in each long read from count extraction (see Methods). Correcting for each of these biases separately strongly shifted the F*upfi* distributions in the expected direction (Figure 4C, D, right panels). Importantly, correction of both biases at the same time retrieved a similar distribution to the one obtained by short reads, i.e. with an excess of *upfi* splicing (Figure 4C; ratio *upfi*/*dofi* = 1.25 versus 1.41; *P* = 0.48, two-sided Fisher's Exact test). This suggests that the observed discrepancies may be due to differences in the sequencing technology (i.e. long versus short read sequencing) and that *Insplico* can correctly account for such differences through its long read bias-correction module (option --biascorr).

### AISO is highly stable across different biological conditions

**Figure 5.**
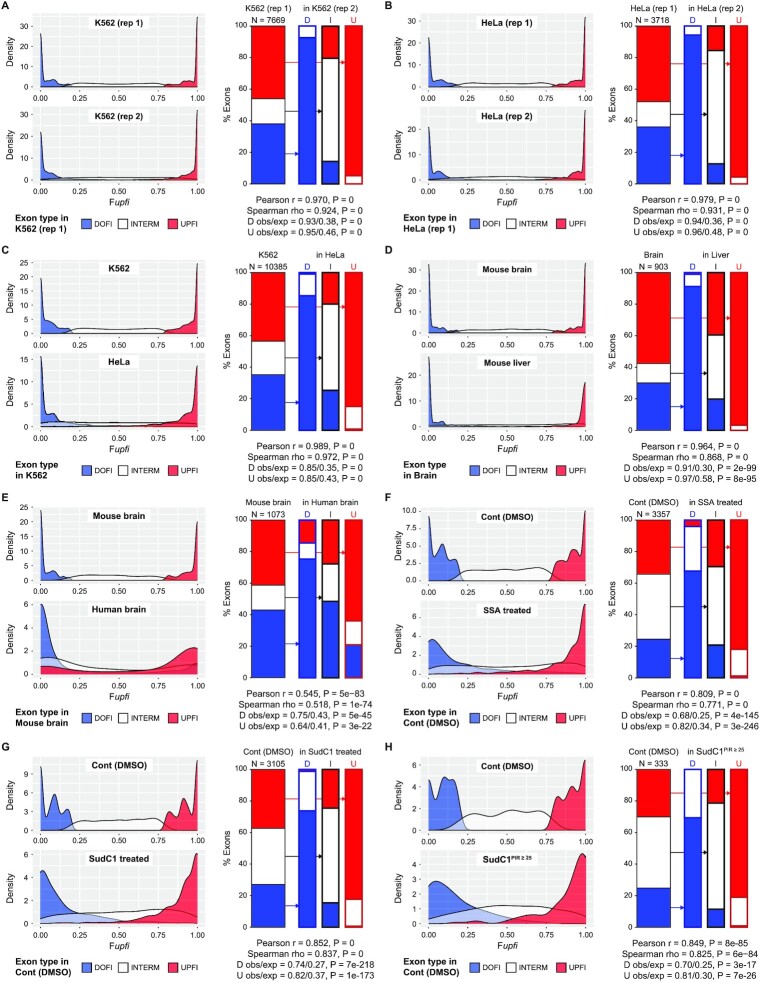
AISO patterns are largely consistent across cell and tissue types and experimental conditions. For each panel (A-H), for a given sample (e.g. ‘K562 (rep 1)’), its AISO profile is derived showing the exons that are strongly *upfi* (red), *dofi* (blue) or intermediate (white), either through *Fupfi* density plots (left) or displaying the proportion of each category using stack plots (right). Then, density plots for *Fupfi* values for the same exons are shown below for the other sample (e.g. ‘K562 (rep 2)’), but each exon is colored according to the AISO pattern in the first sample. Moreover, *upfi*, intermediate, and *dofi* exons in the first sample are separately interrogated in the second sample, as indicated by the arrows (columns ‘U’, ‘I’ and ‘D’, respectively), and their proportion of AISO profiles displayed (further explanations can be found in Supplementary Figure S2A). In addition, results from various statistical tests are provided for each comparison. (**A, B**) Consistent AISO patterns across biological replicates in K562 cells (A, data from (8)) and in HeLa cells (B, data from (31)). (**C, D**) Consistent AISO patterns between human HeLa and K562 cells (C) and mouse brain and liver tissues (D, data from this study). (**E**) Significant evolutionary conservation of AISO patterns among orthologous exons in human and mouse brain (data from (49) and this study). (**F–H**) Consistent AISO patterns in HeLa cells treated with DMSO (control) or spliceostatin (SSA) (F) or sudemycin C1 (SudC1) (G). AISO patterns were largely consistent even for exons for which both neighboring introns were highly affected by drug treatment (ΔPIR > 0.25) (H). Data from (32).

Despite these consistent genome-wide patterns, the AISO of a given exon may vary across different cell/tissue types or conditions, a possibility that has not been investigated yet. To address this, we compared exons with strong *upfi* and *dofi* splicing (F*upfi* ≥ 0.8 or ≤ 0.2, respectively), in various pairs of deep chromatin-associated RNA-seq samples. We reasoned that if AISO is widely maintained between the two samples, the sets of *upfi* (red) and *dofi* (blue) exons in the first sample will tend to be *upfi* and *dofi*, respectively, in the second sample (‘consistent’, Supplementary Figure S2A). Otherwise, in the opposite scenario (‘random’ AISO), the background distribution will be observed in the second sample for each AISO type in the first sample (Supplementary Figure S2A, B). First, we investigated the overall consistency of AISO patterns between replicates of the same studies for exons with sufficient informative reads (N*(upfi + dofi)* ≥ 10) in the two compared samples. Biological replicates of human K562 cells (8) showed extremely consistent AISO patterns (Figures 5A and Supplementary Figure S2C), which can be statistically assessed using a Binomial test for *upfi* or *dofi* exons independently (e.g. 95% of *upfi* exons in replicate 1 are also *upfi* in replicate 2, compared to the 46% expected by chance; *P* = 0, one-sided Binomial test; Figure 5A). This consistency can also be observed by the profile of F*upfi* values in both replicates, in which the same exons in both samples have been colored based on the type of AISO in the first replicate (Figure 5A, density plots). Moreover, AISO values in both samples can be compared using scatter plots, and the global consistency assessed through Pearson and Spearman correlations (Supplementary Figure S3A). For instance, for the two replicates of K562 cells, Pearson's *r* = 0.97 (*P* = 0) and Spearman's *rho* = 0.924 (*P* = 0). Using these complementary metrics, we also found a similarly high consistency of AISO patterns between two RNA-seq replicates of human HeLa cells (31) (Figures 5B, Supplementary Figure S2D and Supplementary Figure S3B). Second, we compared the patterns in K562 and HeLa cells, which also exhibited very consistent AISO distributions (Figures 5C, Supplementary Figure S2E and Supplementary Figure S3C). Third, we aimed at comparing cells of very different origin and function. For this purpose, we generated deep chromatin-bound RNA-seq data from mouse brain (cortex and cerebellum) and liver (Supplementary Table S1), as well as paired cytoplasmic polyA-selected RNA-seq to identify and discard potential intron retention events. Remarkably, *upfi* and *dofi* exons in the brain showed nearly identical patterns in the liver (Figures 5D, Supplementary Figure S2F and Supplementary Figure S3D), suggesting that AISO patterns are highly consistent even across very divergent cell types. Moreover, we assessed if these patterns were conserved between species by comparing AISO between human and mouse brains (Figures 5E, Supplementary Figure S2G and Supplementary Figure S3E). Although the consistency was not as strong as within each species, we found significant conservation of AISO patterns: of 443 *upfi* exons in mouse, 283 were also *upfi* in human (64% versus 41% expected; *P* = 3e–22, one-sided Binomial test), and of 461 *dofi* mouse exons, 347 had their AISO conserved in human (75% versus 43% expected; *P* = 5e–45, one-sided Binomial test). Finally, we investigated whether AISO consistency may be disrupted upon major spliceosomal interference. We used RNA-seq of 5-Bromouridine (BrU)-labelled RNA from HeLa cells treated with two SF3B1-targeting drugs, spliceostatin (SSA) and sudemycin C1 (SudC1), which cause widespread intron retention and exon skipping (32). Even under these conditions, AISO was largely maintained with respect to DMSO-treated control cells (Figures 5F,G, Supplementary Figure S2H, I and Supplementary Figure S3F,G), including the AISO patterns around exons whose immediately upstream and downstream introns were both substantially affected by the treatments (ΔPIR (Treatment – DMSO) ≥ 0.25; Figures 5H, Supplementary Figure S2J and Supplementary Figure S3H).

### Universal genome-wide features of AISO

**Figure 6.**
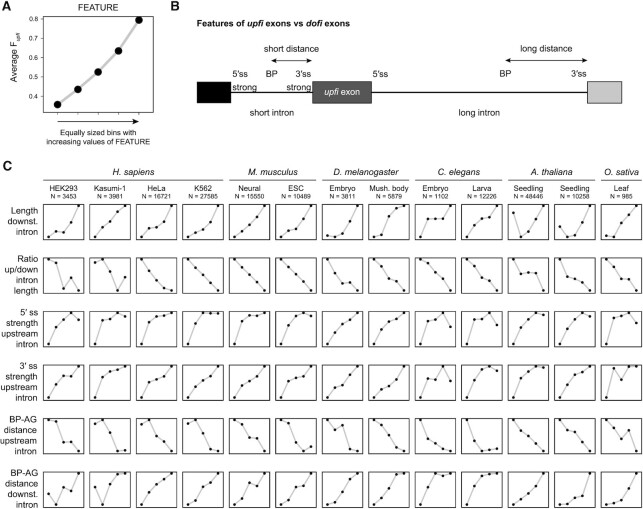
Genomic features universally associated with AISO. (**A**) Schematic representation of the plots obtained in this analysis. For a given feature, the average of F*upfi* values (*Y* axis) is shown for five equal-sized groups of exons binned by increasing values of that feature (*X* axis). Consistently increasing or decreasing associations are considered. (**B**) Summary representation of the features consistently associated with exons whose upstream intron is spliced first. BP, branch point; ss, splice site. (**C**) Summary plots for the main features universally associated with AISO patterns across samples and species. Further details for all features as well as violin plots showing the full F*upfi* distributions are provided in Supplementary File 1.

Given the stability of AISO across different biological conditions, it is likely that its main determinants are to a large extent hardcoded in the genome. Consistently, different sequence features have been previously shown to influence this process (see Introduction). To further identify universal features across conditions and species, we separately processed with *Insplico* 13 short read RNA-seq datasets from nuclear or chromatin-associated RNA fractions from four animals (human, mouse, fruitfly and round worm) and two plants (Arabidopsis and rice) (Supplementary Table S1), which have very different intron densities, genome architectures and alternative splicing patterns (33,34). From each dataset, we considered all truly internal exons with sufficient informative reads, computed their AISO pattern, and extracted 55 splicing-related genomic features using *Matt* (26) (see Methods). To assess the possible contribution of each of these features on AISO, we partitioned for each feature the exons into five equal-sized subsets according to increasing feature values, and plotted the average F*upfi* value per bin (Figure 6A; note that, although we use average F*upfi* values for simplicity, F*upfi* distributions are largely bimodal, as shown in Supplementary File 1 for all features). We identified several genomic features that systematically, linearly or nonlinearly, correlated with AISO with the same functional relationship across all datasets from all species (Figure 6B,C). Specifically, exons whose upstream flanking intron is removed first (*upfi* exons) have well-defined upstream introns with: (i) strong 5′ ss, (ii) strong 3′ ss, and (iii) branch points (BPs) close to their 3′ ss (AG). On the other hand, their downstream introns are (iv) long, (v) particularly with respect to the upstream intron, and (vi) their BP is far from their 3′ ss. Altogether, these results highlight the importance of a strongly defined upstream intron as well as the length and the BP-AG distance of both flanking introns relative to other sequence features.

### Splicing of SRRM4-dependent neural microexons is predominantly *dofi*

**Figure 7.**
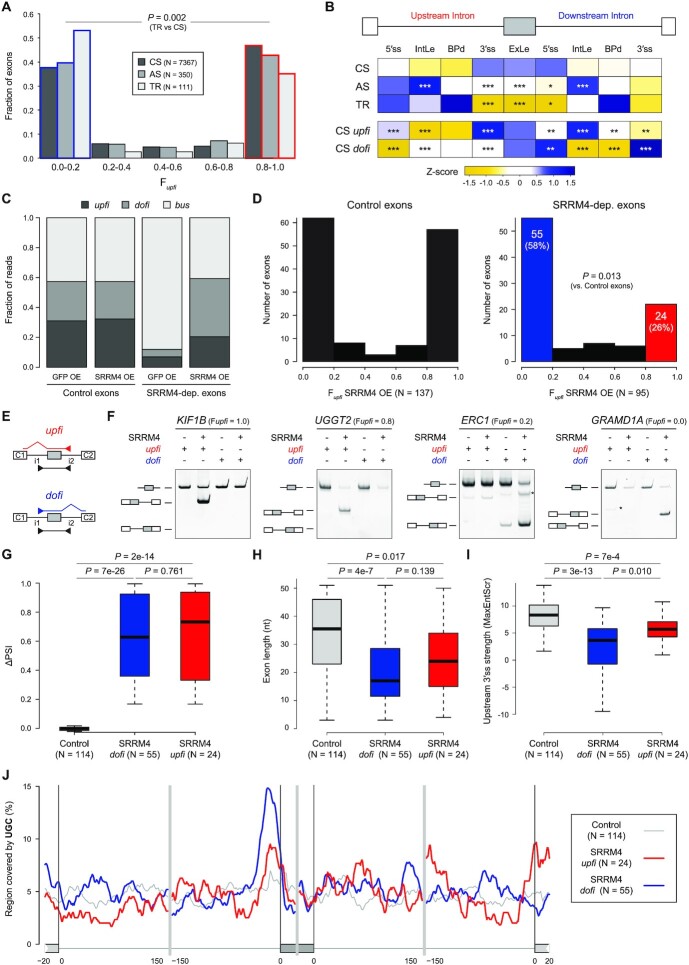
AISO patterns differentiate two subsets of SRRM4-dependent microexons. (**A**) Distribution of constitutive (CS), alternative (AS) and tissue-regulated (TR) exons according to F*upfi* values. P-value corresponds to the comparison between *upfi* (red) and *dofi* (blue) TR vs. CS exons using a two-sided Fisher's Exact test. (**B**) Z-scored median values for each feature for each exon type as well as CS exons that are strongly *upfi* (‘CS *upfi*’) and *dofi* (‘CS *dofi*’). IntLe, intron length; BPd, distance from branch point to 3′ ss; ExLe, exon length. P-values correspond to Bonferroni-corrected Wilcoxon Rank-Sum tests against the CS distributions for each feature. * 0.05 < *P* ≤ 0.01, ** 0.01 < *P* ≤ 0.001, *** *P* < 0.001. All details for all features are provided in Supplementary File 2. (**C**) Distribution of unprocessed reads (*upfi*, *dofi* and *bus*) for control and SRRM4-dependent (SRRM4-dep.) microexons in each condition. (**D**) Distribution of exons according to F*upfi* values for control (left) and SRRM4-dependent (right) microexons in HEK293 cells ectopically expressing human SRRM4. *P*-value corresponds to the comparison between *upfi* (red) and *dofi* (blue) control and SRRM4-dependent exons using a two-sided Fisher's Exact test. (**E, F**) Validation of AISO for four SRRM4-dependent microexons with *upfi* or *dofi* patterns in SRRM4-expressing HEK293 cells. Primers in upstream and downstream introns (i1 and i2) in combination with exon junction primers between the exon C1 upstream and the microexon (*upfi*, red) and between the microexon and the exon C2 downstream (*dofi*, blue) were used for specific detection of partially processed transcripts, as depicted in (E). Unidentified amplification products in F are indicated with an asterisk. (**G–I**) Distributions of ΔPSI (SRRM4 OE vs control; G), exon length (H) and 3′ ss strength of the upstream intron (using the MaxEntScr metric; I) for control and SRRM4-dependent exons with strong *dofi* (blue) and *upfi* (red) patterns. *P*-values correspond to Wilcoxon Rank-Sum tests. (**J**) RNA map showing the percent sequence covered by UGC motifs using a sliding window of 25 nts for each microexon type. For C-J, only microexons with *N(upfi + dofi)* ≥ 5 in the SRRM4 OE sample were considered for all the analyses.

Given these consistent genomic features correlating with AISO and the global consistency in the patterns across cell types, we asked how tissue-specific regulation of alternative splicing relates to AISO. Previous studies have shown that alternatively spliced exons are more often *dofi*, compared to constitutive exons (15). Interestingly, by separating mouse alternatively spliced exons (0.1 < PSI < 0.9 in liver and/or brain) into those that are or that are not regulated in a tissue-dependent manner (|ΔPSI liver versus neural| ≥ 0.25), we found that the excess of *dofi* AISO was only substantial and statistically significant for tissue-regulated (TR) exons and not for all alternatively spliced exons (Figure 7A; TR versus constitutive exons [CS], *P* = 0.002, Fisher's Exact test). However, when looking at the key genomic features identified above for TR exons we found a mix of patterns, indicating that their genomic features cannot solely explain the excess of *dofi* AISO (Figure 7B and Supplementary File 2).

Therefore, we hypothesized that their tissue-specific splicing regulators may impose unique regulatory architectures with non-canonical AISO patterns. To begin assessing this possibility, we focused on a particular case of extreme tissue-specific regulation: neural-specific microexons that are dependent on the splicing factor SRRM4 for their inclusion. The mode of action of the splicing factor SRRM4 is not fully understood, but interactions between its major functional protein domain, the eMIC domain, and early spliceosomal components have been demonstrated (27,35). To shed more light into the mechanism of SRRM4-dependent microexon inclusion, we generated total ribo-depleted RNA-seq data from human HEK293 cells ectopically expressing either GFP or SRRM4 and applied *Insplico* for PSI and F*upfi* estimations. We identified 677 exons that were more included upon SRRM4 overexpression (OE) compared to the control (ΔPSI ≥ 0.15) and that were lowly included in control cells (PSI < 0.2). As expected, most of these (511/677, 76%) were microexons (defined here as length ≤ 51 nts). We then compared these SRRM4-dependent microexons with a control exon set of length ≤ 51 nts, high inclusion in the control (PSI > 0.8) and not affected by SRRM4 OE (|ΔPSI| < 0.1). As expected, by looking at all *Insplico* counts for unprocessed transcripts (i.e. *upfi, dofi*, *bus*; Figure 1), we found that SRRM4-dependent microexons need SRRM4 even for partial processing (Figure 7C). Remarkably, in SRRM4 OE cells, we found that 58% of SRRM4-dependent microexons had strongly *dofi* splicing patterns (F*upfi* ≤ 0.2), while only 26% of them seem to be spliced in an *upfi* manner (F*upfi* ≥ 0.8; Figure 7D), in contrast to roughly equal percentages in the control set (*P* = 0.013, Fisher's Exact test). Importantly, RNA-seq-based AISO patterns were validated experimentally using *upfi* and *dofi* specific primers for four SRRM4-dependent microexons (Figure 7E, F).

We next asked whether *upfi* and *dofi* SRRM4-dependent microexons showed distinct characteristics. First, being *upfi* or *dofi* was not significantly associated with a different magnitude of the response to SRRM4 OE (Figure 7G; *P* = 0.761, Wilcoxon Sum-Rank test). Second, despite all being microexons by definition, *dofi* microexons tended to be shorter than *upfi* microexons (medians 17 versus 24 nts, respectively), although this difference did not reach statistical significance (Figure 7H; *P* = 0.139). Third, a major feature previously associated with SRRM4-dependent microexons, namely weaker 3′ ss contexts in the upstream intron (27,35,36), was significantly more prominent for *dofi* microexons (Figure 7I; *P* = 0.010, Wilcoxon Sum-Rank test; Supplementary File 3), consistent with the general pattern of all *dofi* exons (Figures 6 and 7B). Finally, the presence of the UGC motif near the 3′ ss, also characteristic of eMIC-dependent regulation by SRRM4 (27,35–37), was more prevalent in *dofi* SRRM4-dependent microexons (Figure 7J). In summary, these results suggest that the previously reported features associated with SRRM4 regulation are more associated with *dofi* splicing and raise the possibility that two modes of regulation by SRRM4 may exist in connection with opposite AISO patterns. However, further studies are required to increase the robustness of these conclusions and evaluate their mechanistic relevance.

## DISCUSSION

We developed *Insplico*, the first publicly available standalone software dedicated to the study of the splicing order of adjacent introns applicable to both short and long read sequencing technologies, and demonstrated its effectiveness using simulated reads and by comparing it with two published studies of AISO (15,16). Some previous studies had reported methods and code for the analysis of AISO with either short or long reads (13,15,16). However, *Insplico* facilitates the analysis of AISO by providing a single user-friendly and standalone tool that can process both types of sequencing technologies and that includes additional functionality. Importantly, despite specifically studying intron splicing, *Insplico* is an exon-centric, not intron-centric tool. It obtains key measurements to investigate pre-mRNA processing around user-specified exons of interest (*upfi*, *dofi*, *bus* and *bos* counts) and estimates the fraction of transcripts in which the upstream intron is spliced before the downstream one (F*upfi*). Moreover, another unique feature of *Insplico* is that it also quantifies the level of inclusion of exons (PSI) and the level of intron retention (PIR) of the two neighboring introns, allowing more complex integrated analyses as well as further filtering and stratification strategies within a single software.

Contrary to short reads, which only give local information, long reads have the potential to provide a snapshot of all introns of a transcript at once, thus being particularly promising to study intron splicing order. Intriguingly, a pioneer study (16) obtained AISO patterns in human using long reads that did not match those previously reported for short reads. Here, we showed that such discrepancies could be due to 5′ and 3′ biases in long read sequencing and library preparation that, when corrected for, harmonize the patterns obtained by both types of sequencing technologies. The optional module to perform these corrections is a unique feature of *Insplico*. On the other hand, short read sequencing has its own limitations. For example, given that *upfi* and *dofi* fragments require mapping across the entire exon to cover both splice sites (Figure 1A), the length of the reads (and the fragment size of paired-end reads) limits the maximum length of the exons that are quantifiable by *Insplico* using short reads, as we illustrated using simulated reads. Moreover, although we have explicitly focused on the relative splicing order of the pairs of introns directly flanking the input exons (AISO), short reads can barely provide splicing order information beyond them as long reads can do (38).

After demonstrating its effectiveness, we applied *Insplico* to multiple public and newly generated RNA-seq datasets to gain insights into AISO across cell types, conditions and species. Splicing is known to mainly occur co-transcriptionally and be influenced by transcriptional related features, RNA *cis*-acting elements and binding proteins. How these factors determine AISO and how it varies across different conditions is largely unknown. Remarkably, through our analyses, we not only observed a strong consistency of AISO across cell and tissue types from the same species, but also even under disruptive spliceosomal conditions such as those induced by SF3B1-targeting drugs. Thus, these findings suggest that, even though experimentally induced changes in AISO have been shown to affect splicing outcomes (20,22), AISO is largely independent of the splicing outcome under physiological and non-physiological conditions. Moreover, these results argue that, for most exons, the main contributors to this predefined AISO are likely hardcoded in the genome. In line with this idea, our cross-species analysis revealed several universal genomic features that are strongly associated with exons whose upstream intron is spliced before the downstream one across various animal and plant species. In particular, such exons are typically flanked by a short upstream intron with strong 5′ ss and 3′ ss (including a long polypyrimidine tract and short BP-AG distance) and a long downstream intron with large BP-AG distance, but otherwise regular splice sites. Those features are generally in line with and expand previous studies (15,16,39), and are consistent with the model known as ‘first come, first spliced’, whereby a co-transcriptional recruitment of the spliceosome occurs and triggers the splicing of well-defined introns as soon as they are fully transcribed (10–14). However, consistent with previous studies (13,15,16), we also found that there are nearly as many exons whose downstream introns are spliced prior to the upstream one as there are of the converse ‘canonical’ case, overall resulting in a strong bimodal distribution of F*upfi* values genome-wide for all studied species. This large number of *dofi* exons, which are particularly associated with tissue-regulated alternative exons, is consistent with previous studies indicating large fractions of delayed or post-transcriptional splicing (40) and the exceptional dependence of these introns on specific *trans*-acting factors for their splicing (1,41). Therefore, to fully comprehend the universal AISO code, it will be necessary to not only dissect the role of spliceosomal components and RBPs in intron splicing order (15,16,38), but also to expand and integrate recently developed mathematical modelling of co-transcriptional splicing (42) and splicing decisions based on RBP binding rate and position on the pre-mRNA (43) with chromatin features (44,45), spliceosomal recruitment and splicing kinetics (46,47).

As an example of *trans*-acting dependent splicing regulation, we focused here on a unique case of tissue-regulated exons, neural microexons, which are characterized by their short length (defined here as smaller than 51 nts) and dependence on SRRM4 for inclusion (36). An appealing hypothesis, which we investigated here, is that these microexons are highly dependent on a particular AISO for inclusion. Along this line, and consistent with the pattern of AISO for TR exons, we observed that the majority of SRRM4-dependent microexons are preferentially spliced in a *dofi* manner. Such *dofi* microexons tend to be shorter, have significantly weaker 3′ ss, and much more marked UGC peak at a shorter distance to the 5′ ss than *upfi* ones. This suggests that the simultaneous assembly of U1 and U2 snRNPs is not possible on the surrounding 5′ and 3′ splice sites of these microexons due to steric hindrance. Therefore, it could be possible that they are mainly recognized at the level of their 5′ ss, which are known to be particularly strong (27,36), by the U1 snRNP whose recruitment could then favor splicing of the downstream intron. After splicing of the downstream intron and, possibly, recruitment of RNPS1 (a component of the Exon Junction Complex) (48), SRRM4 binding could favor 3′ ss recognition by promoting the recruitment of early spliceosomal components (27) and subsequent splicing of the upstream intron. Intriguingly, however, a non-negligible fraction of SRRM4-dependent microexons are spliced in an *upfi* manner. Those tend to be longer, with stronger 3′ ss and a less marked UGC peak, suggesting that they may be recognized and spliced in a way more similar to constitutive exons. Although those hypotheses remain to be formally tested, our AISO analysis thus suggests two possible mechanisms of splicing of SRRM4-dependent neural microexons.

## DATA AVAILABILITY

RNA seq data have been deposited to Gene Expression Omnibus (GEO) and are accessible under the ID GSE207459. Scripts for reproducing simulated data can be found at https://github.com/liniguez/Insplico_simulations and https://doi.org/10.5281/zenodo.7759745.

## Supplementary Material

gkad244_Supplemental_Files

## References

[1] Raj B. , Blencowe B.J. Alternative splicing in the mammalian nervous system: recent insights into mechanisms and functional roles. Neuron. 2015; 87:14–27. 26139367 10.1016/j.neuron.2015.05.004

[2] Maniatis T. , Reed R. An extensive network of coupling among gene expression machines. Nature. 2002; 416:499–506. 11932736 10.1038/416499a

[3] Naftelberg S. , Schor I.E., Ast G., Kornblihtt A.R. Regulation of alternative splicing through coupling with transcription and chromatin structure. Annu. Rev. Biochem. 2015; 84:165–198. 26034889 10.1146/annurev-biochem-060614-034242

[4] Herzel L. , Neugebauer K.M. Quantification of co-transcriptional splicing from RNA-Seq data. Methods. 2015; 85:36–43. 25929182 10.1016/j.ymeth.2015.04.024

[5] Khodor Y.L. , Rodriguez J., Abruzzi K.C., Tang C.H., Marr M.T. 2nd, Rosbash M. Nascent-seq indicates widespread cotranscriptional pre-mRNA splicing in Drosophila. Genes Dev. 2011; 25:2502–2512. 22156210 10.1101/gad.178962.111 PMC3243060

[6] Godoy Herz M.A. , Kubaczka M.G., Brzyzek G., Servi L., Krzyszton M., Simpson C., Brown J., Swiezewski S., Petrillo E., Kornblihtt A.R Light regulates plant alternative splicing through the control of transcriptional elongation. Mol. Cell. 2019; 73:1066–1074. 30661982 10.1016/j.molcel.2018.12.005

[7] Ameur A. , Zaghlool A., Halvardson J., Wetterbom A., Gyllensten U., Cavelier L., Feuk L. Total RNA sequencing reveals nascent transcription and widespread co-transcriptional splicing in the human brain. Nat. Struct. Mol. Biol. 2011; 18:1435–1440. 22056773 10.1038/nsmb.2143

[8] Tilgner H. , Knowles D.G., Johnson R., Davis C.A., Chakrabortty S., Djebali S., Curado J., Snyder M., Gingeras T.R., Guigo R. Deep sequencing of subcellular RNA fractions shows splicing to be predominantly co-transcriptional in the human genome but inefficient for lncRNAs. Genome Res. 2012; 22:1616–1625. 22955974 10.1101/gr.134445.111 PMC3431479

[9] Beyer A.L. , Osheim Y.N. Splice site selection, rate of splicing, and alternative splicing on nascent transcripts. Genes Dev. 1988; 2:754–765. 3138163 10.1101/gad.2.6.754

[10] Hoffman B.E. , Grabowski P.J. U1 snRNP targets an essential splicing factor, U2AF65, to the 3' splice site by a network of interactions spanning the exon. Genes Dev. 1992; 6:2554–2568. 1285125 10.1101/gad.6.12b.2554

[11] Neugebauer K.M. Nascent RNA and the coordination of splicing with transcription. Cold Spring Harb. Perspect. Biol. 2019; 11:a032227. 31371351 10.1101/cshperspect.a032227 PMC6671939

[12] Nasim F.H. , Spears P.A., Hoffmann H.M., Kuo H.C., Grabowski P.J. A Sequential splicing mechanism promotes selection of an optimal exon by repositioning a downstream 5' splice site in preprotachykinin pre-mRNA. Genes Dev. 1990; 4:1172–1184. 2210374 10.1101/gad.4.7.1172

[13] Li M. Calculating the most likely intron splicing orders in S. pombe, fruit fly, *Arabidopsis thaliana*, and humans. BMC Bioinf. 2020; 21:478. 10.1186/s12859-020-03818-6 PMC7585206 33099301

[14] Kessler O. , Jiang Y., Chasin L.A. Order of intron removal during splicing of endogenous adenine phosphoribosyltransferase and dihydrofolate reductase pre-mRNA. Mol. Cell Biol. 1993; 13:6211–6222. 8413221 10.1128/mcb.13.10.6211-6222.1993 PMC364680

[15] Kim S.W. , Taggart A.J., Heintzelman C., Cygan K.J., Hull C.G., Wang J., Shrestha B., Fairbrother W.G. Widespread intra-dependencies in the removal of introns from human transcripts. Nucleic Acids Res. 2017; 45:9503–9513. 28934498 10.1093/nar/gkx661 PMC5766209

[16] Drexler H.L. , Choquet K., Churchman L.S. Splicing kinetics and coordination revealed by direct nascent RNA sequencing through nanopores. Mol. Cell. 2020; 77:985–998. 31839405 10.1016/j.molcel.2019.11.017 PMC7060811

[17] Schor I.E. , Gomez Acuna L.I., Kornblihtt A.R. Coupling between transcription and alternative splicing. Cancer Treat. Res. 2013; 158:1–24. 24222352 10.1007/978-3-642-31659-3_1

[18] Fong N. , Kim H., Zhou Y., Ji X., Qiu J., Saldi T., Diener K., Jones K., Fu X.D., Bentley D.L. Pre-mRNA splicing is facilitated by an optimal RNA polymerase II elongation rate. Genes Dev. 2014; 28:2663–2676. 25452276 10.1101/gad.252106.114 PMC4248296

[19] Maslon M.M. , Braunschweig U., Aitken S., Mann A.R., Kilanowski F., Hunter C.J., Blencowe B.J., Kornblihtt A.R., Adams I.R., Caceres J.F. A slow transcription rate causes embryonic lethality and perturbs kinetic coupling of neuronal genes. EMBO J. 2019; 38:e101244. 30988016 10.15252/embj.2018101244 PMC6484407

[20] Takahara K. , Schwarze U., Imamura Y., Hoffman G.G., Toriello H., Smith L.T., Byers P.H., Greenspan D.S. Order of intron removal influences multiple splice outcomes, including a two-exon skip, in a COL5A1 acceptor-site mutation that results in abnormal pro-alpha1(V) N-propeptides and Ehlers-Danlos syndrome type I. Am. J. Hum. Genet. 2002; 71:451–465. 12145749 10.1086/342099 PMC379186

[21] Boehm V. , Britto-Borges T., Steckelberg A.L., Singh K.K., Gerbracht J.V., Gueney E., Blazquez L., Altmuller J., Dieterich C., Gehring N.H. Exon junction complexes suppress spurious splice sites to safeguard transcriptome integrity. Mol. Cell. 2018; 72:482–495. 30388410 10.1016/j.molcel.2018.08.030

[22] Blazquez L. , Emmett W., Faraway R., Pineda J.M.B., Bajew S., Gohr A., Haberman N., Sibley C.R., Bradley R.K., Irimia M. et al. Exon junction complex shapes the transcriptome by repressing recursive splicing. Mol. Cell. 2018; 72:496–509. 30388411 10.1016/j.molcel.2018.09.033 PMC6224609

[23] Tapial J. , Ha K.C.H., Sterne-Weiler T., Gohr A., Braunschweig U., Hermoso-Pulido A., Quesnel-Vallières M., Permanyer J., Sodaei R., Marquez Y. et al. An atlas of alternative splicing profiles and functional associations reveals new regulatory programs and genes that simultaneously express multiple major isoforms. Genome Res. 2017; 27:1759–1768. 28855263 10.1101/gr.220962.117 PMC5630039

[24] Danecek P. , Bonfield J.K., Liddle J., Marshall J., Ohan V., Pollard M.O., Whitwham A., Keane T., McCarthy S.A., Davies R.M. et al. Twelve years of SAMtools and BCFtools. Gigascience. 2021; 10:giab008. 33590861 10.1093/gigascience/giab008 PMC7931819

[25] Li H. Minimap2: pairwise alignment for nucleotide sequences. Bioinformatics. 2018; 34:3094–3100. 29750242 10.1093/bioinformatics/bty191 PMC6137996

[26] Gohr A. , Irimia M. Matt: unix tools for alternative splicing analysis. Bioinformatics. 2019; 35:130–132. 30010778 10.1093/bioinformatics/bty606

[27] Torres-Méndez A. , Bonnal S., Marquez Y., Roth J., Iglesias M., Permanyer J., Almudí I., O’Hanlon D., Guitart T., Soller M. et al. A novel protein domain in an ancestral splicing factor drove the evolution of neural microexons. Nature Ecol. Evol. 2019; 3:691–701. 30833759 10.1038/s41559-019-0813-6

[28] Head S.A. , Hernandez-Alias X., Yang J.S., Ciampi L., Beltran-Sastre V., Torres-Mendez A., Irimia M., Schaefer M.H., Serrano L. Silencing of SRRM4 suppresses microexon inclusion and promotes tumor growth across cancers. PLoS Biol. 2021; 19:e3001138. 33621242 10.1371/journal.pbio.3001138 PMC7935315

[29] Girard C. , Mouaikel J., Neel H., Bertrand E., Bordonne R. Nuclear localization properties of a conserved protuberance in the Sm core complex. Exp. Cell. Res. 2004; 299:199–208. 15302587 10.1016/j.yexcr.2004.05.018

[30] Hsiao Y.E. , Bahn J.H., Yang Y., Lin X., Tran S., Yang E.W., Quinones-Valdez G., Xiao X. RNA editing in nascent RNA affects pre-mRNA splicing. Genome Res. 2018; 28:812–823. 29724793 10.1101/gr.231209.117 PMC5991522

[31] Ke S. , Pandya-Jones A., Saito Y., Fak J.J., Vagbo C.B., Geula S., Hanna J.H., Black D.L., Darnell J.E. Jr, Darnell R.B m(6)A mRNA modifications are deposited in nascent pre-mRNA and are not required for splicing but do specify cytoplasmic turnover. Genes Dev. 2017; 31:990–1006. 28637692 10.1101/gad.301036.117 PMC5495127

[32] Vigevani L. , Gohr A., Webb T., Irimia M., Valcarcel J. Molecular basis of differential 3' splice site sensitivity to anti-tumor drugs targeting U2 snRNP. Nat. Commun. 2017; 8:2100. 29235465 10.1038/s41467-017-02007-z PMC5727392

[33] Martín G. , Márquez Y., Mantica F., Duque P., Irimia M. Alternative splicing landscapes in *Arabidopsis thaliana* across tissues and stress conditions highlight major functional differences with animals. Genome Biol. 2021; 22:35. 33446251 10.1186/s13059-020-02258-y PMC7807721

[34] Grau-Bove X. , Ruiz-Trillo I., Irimia M. Origin of exon skipping-rich transcriptomes in animals driven by evolution of gene architecture. Genome Biol. 2018; 19:135. 30223879 10.1186/s13059-018-1499-9 PMC6142364

[35] Raj B. , Irimia M., Braunschweig U., Sterne-Weiler T., O’Hanlon D., Yuan-Lin Z., Chen I.G., Easton L., Ule J., Gingras A.C. et al. Global regulatory mechanism underlying the activation of an exon network required for neurogenesis. Mol. Cell. 2014; 56:90–103. 25219497 10.1016/j.molcel.2014.08.011 PMC4608043

[36] Irimia M. , Weatheritt R.J., Ellis J., Parikshak N.N., Gonatopoulos-Pournatzis T., Babor M., Quesnel-Vallières M., Tapial J., Raj B., O’Hanlon D. et al. A highly conserved program of neuronal microexons is misregulated in autistic brains. Cell. 2014; 159:1511–1523. 25525873 10.1016/j.cell.2014.11.035 PMC4390143

[37] Nakano Y. , Jahan I., Bonde G., Sun X., Hildebrand M.S., Engelhardt J.F., Smith R.J., Cornell R.A., Fritzsch B., Bánfi B. A mutation in the Srrm4 gene causes alternative splicing defects and deafness in the Bronx waltzer mouse. PLoS Genet. 2012; 8:e1002966. 23055939 10.1371/journal.pgen.1002966 PMC3464207

[38] Choquet K. , Koenigs A., Dülk S.L., Smalec B.M., Rouskin S., Churchman L.S. Pre-mRNA splicing order is predetermined and maintains splicing fidelity across multi-intronic transcripts. 2022; bioRxiv doi:12 August 2022, preprint: not peer reviewed 10.1101/2022.08.12.503515. PMC10653200 37443198

[39] Zeng Y. , Fair B.J., Zeng H., Krishnamohan A., Hou Y., Hall J.M., Ruthenburg A.J., Li Y.I., Staley J.P. Profiling lariat intermediates reveals genetic determinants of early and late co-transcriptional splicing. Mol. Cell. 2022; 82:4681–4699. 36435176 10.1016/j.molcel.2022.11.004 PMC10448999

[40] Vargas D.Y. , Shah K., Batish M., Levandoski M., Sinha S., Marras S.A., Schedl P., Tyagi S. Single-molecule imaging of transcriptionally coupled and uncoupled splicing. Cell. 2011; 147:1054–1065. 22118462 10.1016/j.cell.2011.10.024 PMC3245879

[41] Gordon J.M. , Phizicky D.V., Neugebauer K.M. Nuclear mechanisms of gene expression control: pre-mRNA splicing as a life or death decision. Curr. Opin. Genet. Dev. 2021; 67:67–76. 33291060 10.1016/j.gde.2020.11.002 PMC8084925

[42] Davis-Turak J. , Johnson T.L., Hoffmann A. Mathematical modeling identifies potential gene structure determinants of co-transcriptional control of alternative pre-mRNA splicing. Nucleic Acids Res. 2018; 46:10598–10607. 30272246 10.1093/nar/gky870 PMC6237756

[43] Horn T. , Gosliga A., Li C., Enculescu M., Legewie S. Position-dependent effects of RNA-binding proteins in the context of co-transcriptional splicing. NPJ Syst. Biol. Appl. 2023; 9:1. 36653378 10.1038/s41540-022-00264-3 PMC9849329

[44] Agirre E. , Oldfield A.J., Bellora N., Segelle A., Luco R.F. Splicing-associated chromatin signatures: a combinatorial and position-dependent role for histone marks in splicing definition. Nat. Commun. 2021; 12:682. 33514745 10.1038/s41467-021-20979-x PMC7846797

[45] Petrova V. , Song R., Consortium D., Nordstrom K.J.V., Walter J., Wong J.J.L., Armstrong N.J., Rasko J.E.J., Schmitz U. Increased chromatin accessibility facilitates intron retention in specific cell differentiation states. Nucleic Acids. Res. 2022; 50:11563–11579. 36354002 10.1093/nar/gkac994 PMC9723627

[46] Wachutka L. , Caizzi L., Gagneur J., Cramer P. Global donor and acceptor splicing site kinetics in human cells. Elife. 2019; 8:e45056. 31025937 10.7554/eLife.45056 PMC6548502

[47] Wickramasinghe V.O. , Gonzalez-Porta M., Perera D., Bartolozzi A.R., Sibley C.R., Hallegger M., Ule J., Marioni J.C., Venkitaraman A.R. Regulation of constitutive and alternative mRNA splicing across the human transcriptome by PRPF8 is determined by 5' splice site strength. Genome Biol. 2015; 16:201. 26392272 10.1186/s13059-015-0749-3 PMC4578845

[48] Gonatopoulos-Pournatzis T. , Wu M., Braunschweig U., Roth J., Han H., Best A.J., Raj B., Aregger M., O’Hanlon D., Ellis J.D. et al. Genome-wide CRISPR-Cas9 interrogation of splicing networks reveals a mechanism for recognition of autism-misregulated neuronal microexons. Mol. Cell. 2018; 72:510–524. 30388412 10.1016/j.molcel.2018.10.008

[49] Burke E.E. , Chenoweth J.G., Shin J.H., Collado-Torres L., Kim S.K., Micali N., Wang Y., Colantuoni C., Straub R.E., Hoeppner D.J. et al. Dissecting transcriptomic signatures of neuronal differentiation and maturation using iPSCs. Nat. Commun. 2020; 11:462. 31974374 10.1038/s41467-019-14266-z PMC6978526

