# Supplementary material for: Insplico: effective computational tool for studying splicing order of adjacent introns genome-wide with short and long RNA-seq reads
Source: Nucleic Acids Res. 2023 Apr 7;51(10):e56. doi: 10.1093/nar/gkad244 (PMC10250204; doi:10.1093/nar/gkad244)
Supplement: gkad244_Supplemental_Files [file gkad244_supplemental_files.zip › Gohr_et_al-Sup_Materials-R1.pdf]

**Supplementary Materials for:**

***Insplico*: Effective computational tool for studying splicing order of adjacent introns genome-wide with short and long RNA-seq reads**

Andre Gohr[1], Luis P. Iñiguez, Antonio Torres-Méndez[1], Sophie Bonnal[1,4], and Manuel Irimia[1,2,3,4]

1 - Centre for Genomic Regulation (CRG), The Barcelona Institute of Science and Technology, Barcelona, Spain.

2 - Universitat Pompeu Fabra (UPF), Barcelona, Spain.

3 - ICREA, Barcelona, Spain.

4 - Corresponding authors: mirimia@gmail.com, sophie.bonnal@crg.eu.

**Table S1 - RNA-seq samples used in this study.**

**Supplementary File 1 -** Plots showing the associations between AISO patterns and all features as obtained by *Matt cmpr_features*. Further methodological details as well as exon sample sizes are included.

**Supplementary File 2 -** Report from *Matt cmpr_exons* for constitutive (CS), alternative (AS) and tissue-regulated (TR) exons, CS *upfi* and CS *dofi* exons.

**Supplementary File 3 -** Report from *Matt cmpr_exons* for control and SRRM4-dependent *upfi* and *dofi* microexons.

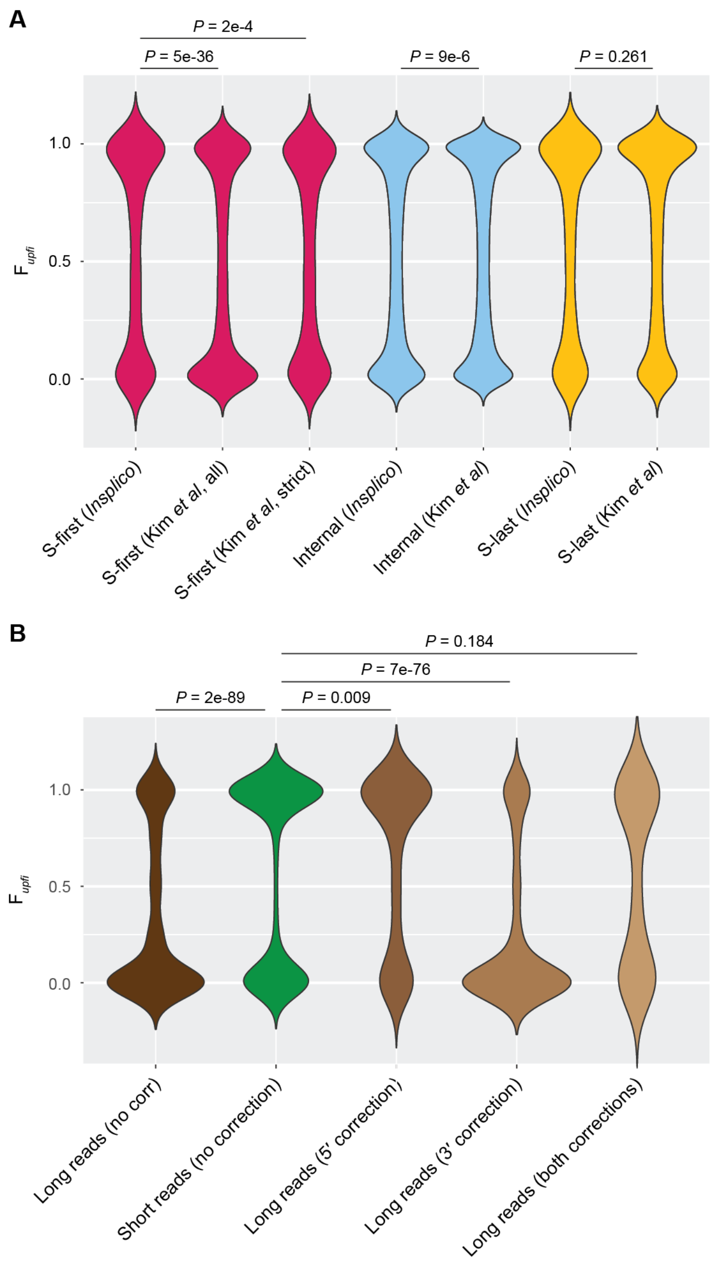


**Figure S1 - Violin plots of F*upfi* distributions for Figs. 3 and 4**

**A,B**) Violin plots showing the F*upfi* values for each of the histograms from Fig. 3 (A) or Fig. 4 (B). P-values correspond to Wilcoxon sum-rank tests.

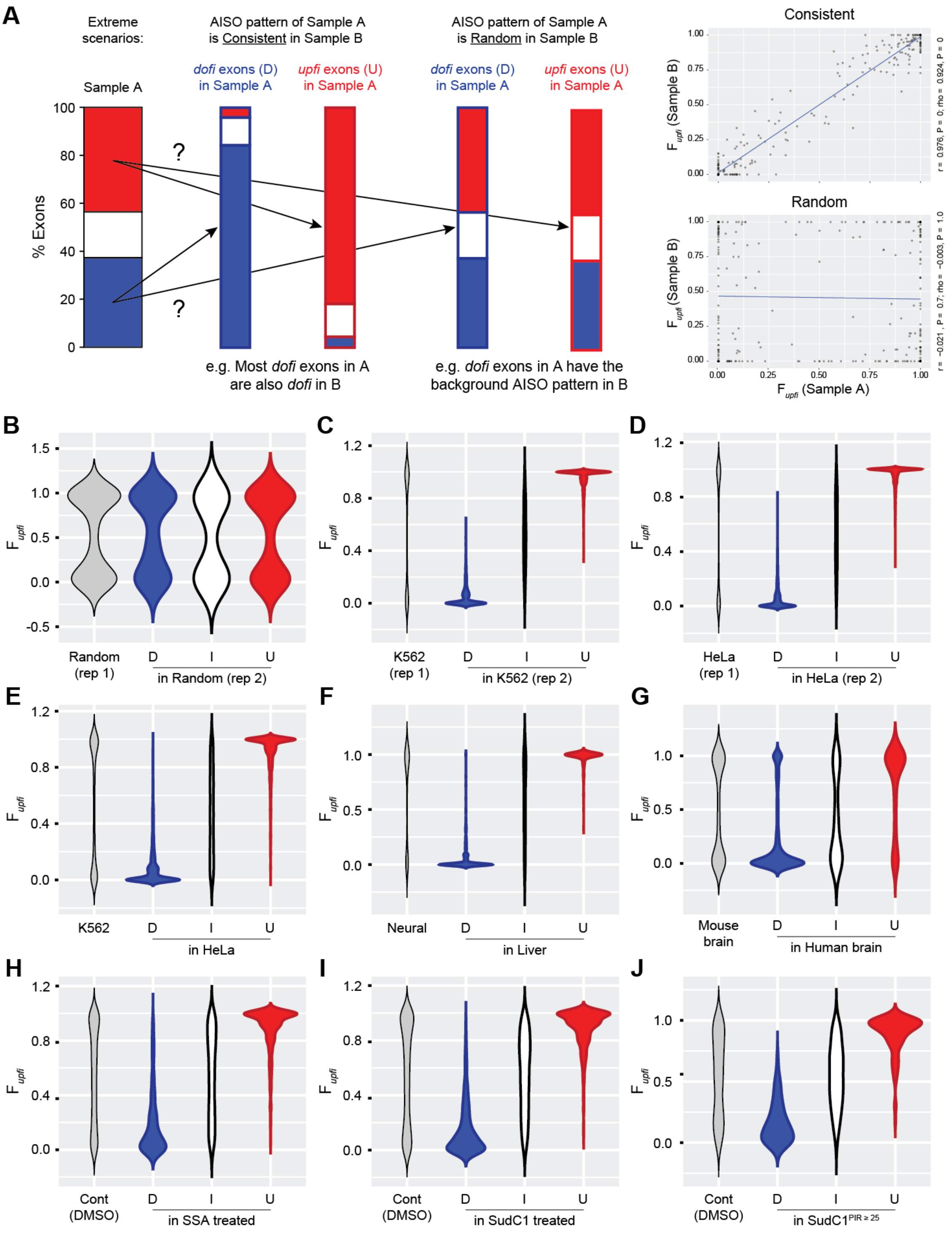


**Figure S2 - AISO is highly consistent across samples**

**A**) Schematic summary of the analysis shown as stack plots in Fig. 5. For a given Sample A, its AISO profile is derived showing the proportion of exons that are strongly *upfi* (red), *dofi* (blue) or intermediate (white). Then, these three groups of exons are separately interrogated in a different Sample B, and their AISO profiles derived in the same manner. Two extreme

patterns can be envisioned: "Consistent", in which most *upfi* exons in Sample A will also be *upfi* in Sample B (and the same for *dofi* exons), and "Random", in which *upfi* and *dofi* exons separately will reproduce the background distribution in Sample B. Right: scattered plots showing examples of consistent (top) and random (bottom) patterns comparing F*upfi* values for the same exons in two samples. **B**) Violin plots showing the distributions of F*upfi* values for the example of two samples with random AISO patterns. **C-J**) Violin plots showing the distribution of F*upfi* values for the exon groups displayed as stack plots in Fig. 5, comparing replicates of K562 cells (C, data from (8)), replicates of HeLa cells (D, data from (23)), human HeLa and K562 cells (E), mouse brain and liver tissues (F; data from this study), orthologous exons in human and mouse brain (G; data from (49) and this study), HeLa cells treated with DMSO (control) and spliceostatin (SSA)(H) or sudemycin C1 (SudC1)(I), and for exons for which both neighboring introns were highly affected by SudC1treatment ($\Delta$PIR > 0.25)(J). Data from (24).

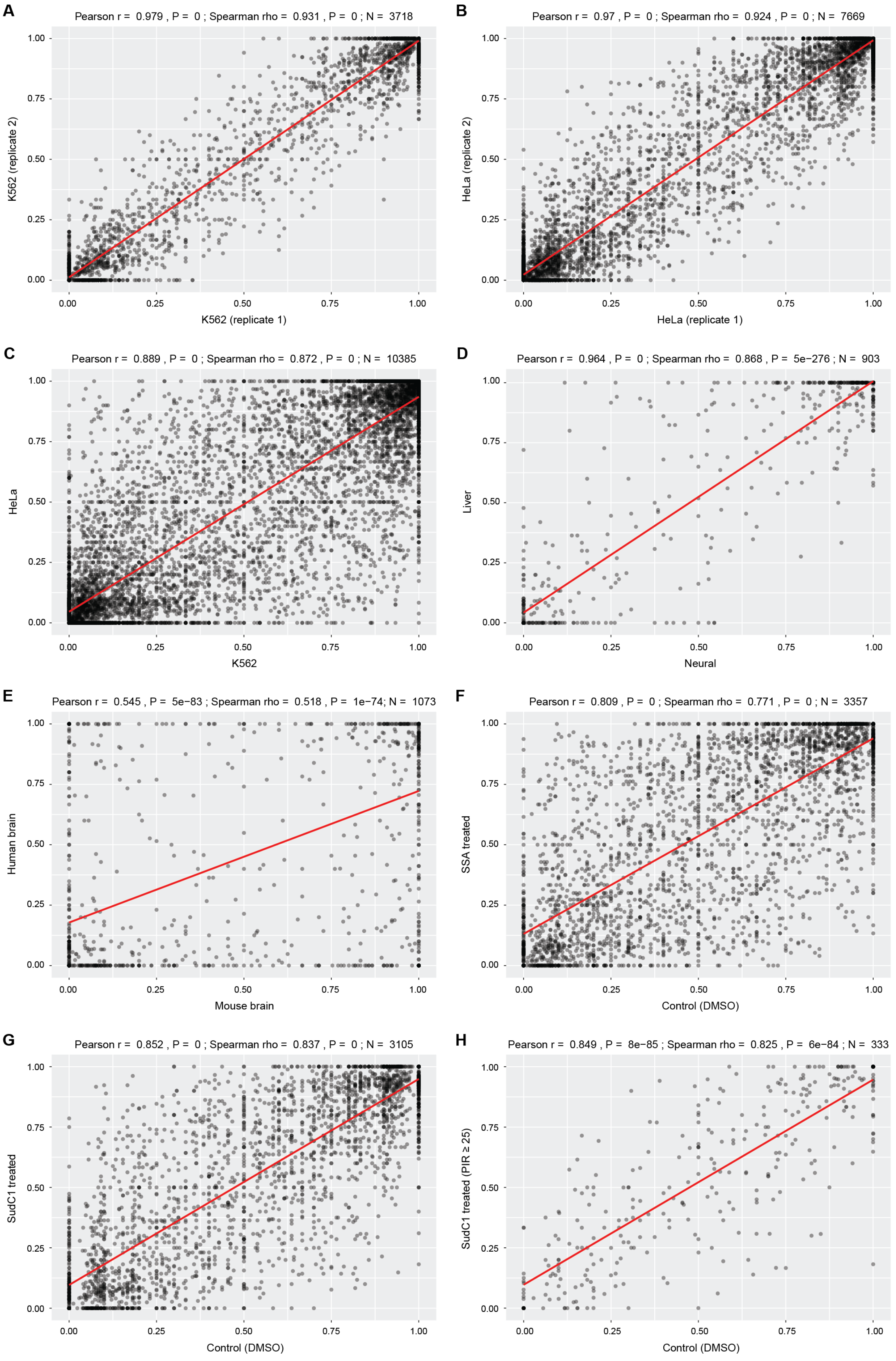


**Figure S3 - Scattered plots of F*upfi* values for pairs of compared samples**

**A-H**) Scatter plots showing F*upfi* values for all the exons compared between pairs of samples in Fig. 5, including replicates of K562 cells (A, data from (8)), replicates of HeLa cells (B, data from (23)), human HeLa and K562 cells (C), mouse brain and liver tissues (D; data from this study), orthologous exons in human and mouse brain (E; data from (49) and this study), HeLa cells treated with DMSO (control) and spliceostatin (SSA)(F) or sudemycin C1 (SudC1)(G), and for exons for which both neighboring introns were highly affected by SudC1treatment ($\Delta$PIR > 0.25)(H). Data from (24).
