# Supplementary material for: Insplico: effective computational tool for studying splicing order of adjacent introns genome-wide with short and long RNA-seq reads
Source: Nucleic Acids Res. 2023 Apr 7;51(10):e56. doi: 10.1093/nar/gkad244 (PMC10250204; doi:10.1093/nar/gkad244)

1 Which exon related features affect intron removal order?

The plots show avg(Fupfi) for up to 5 groups of exons defined by increasing feature values. E.g.:

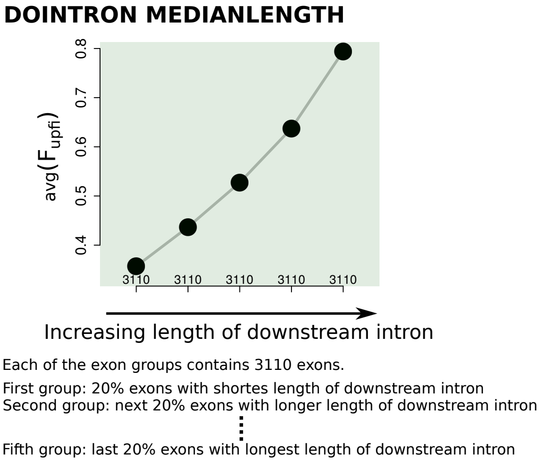

All RNA-Seq data sets come from the nuclear or chromatin RNA fraction. Exons considered here are those for which we have sufficient read support ($N_{upfidofi} > 9$, exception Chen: $N_{upfidofi} > 4$) in Insplico analyses. For data sets Jia, Ke, Tilgner, Bonnal, McCorcindale we had in addition poly-A selected RNA-seq data from the cytoplasm. For these data sets, the set of considered exons was further reduced to those with both flanking introns with PIR<10%. Green color indicates statistical significance with FDR $\leq$ 5% applying the Kruskal-Wallis rank sum across all tests. Features highlighted in red seem most consistent across all data sets of this study.

FUPFI HISTOGRAMS NUPFIDOFI > 9 (> 4 for Chen)

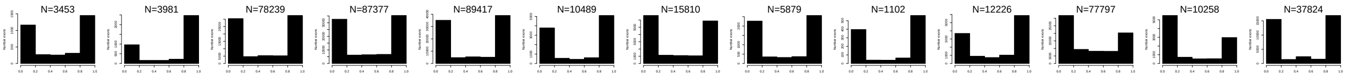

FUPFI HISTOGRAMS NUPFIDOFI > 9 (> 4 for Chen) $\wedge$ flanking PIR<10%

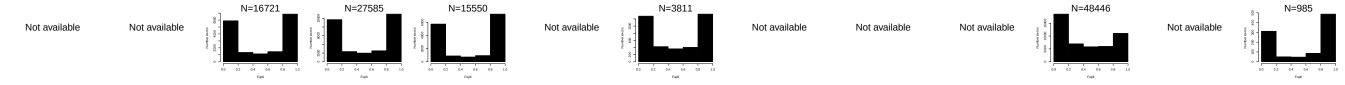

EXON LENGTH

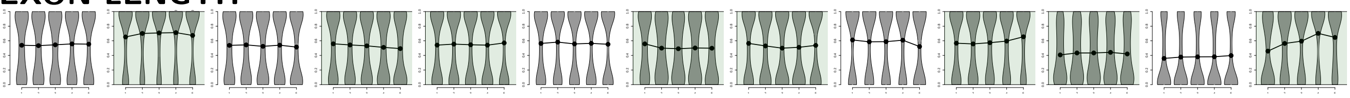

UPEXON MEDIANLENGTH

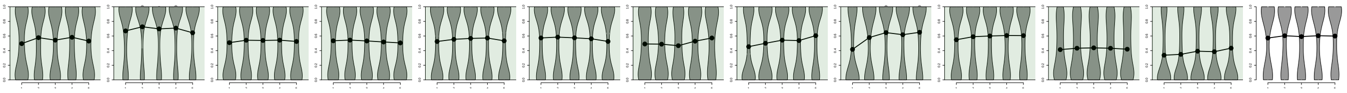

DOEXON MEDIANLENGTH

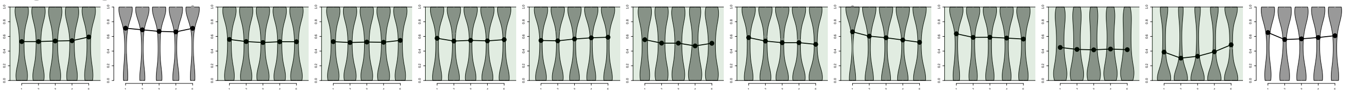

RATIO UPEXLEN DOEXLEN

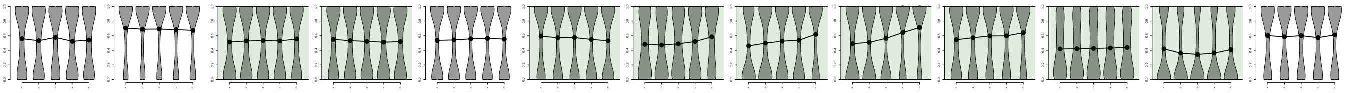

RATIO UPEXON EXON LENGTH

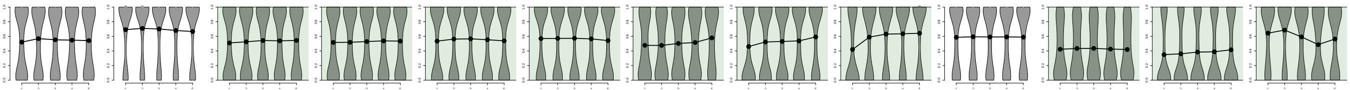

RATIO DOEXON EXON LENGTH

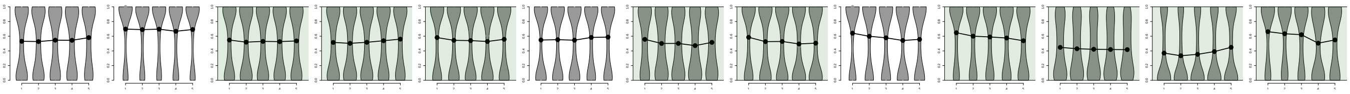

UPINTRON MEDIANLENGTH

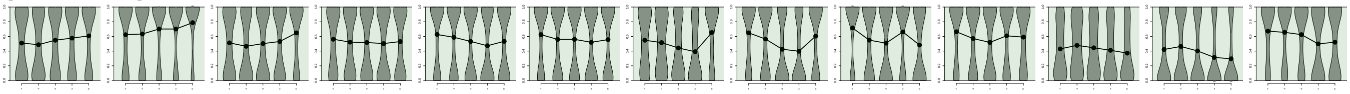

DOINTRON MEDIANLENGTH

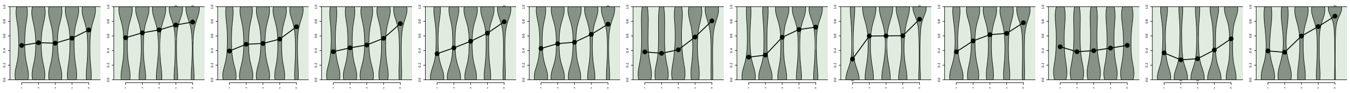

RATIO UPINTRON EXON LENGTH

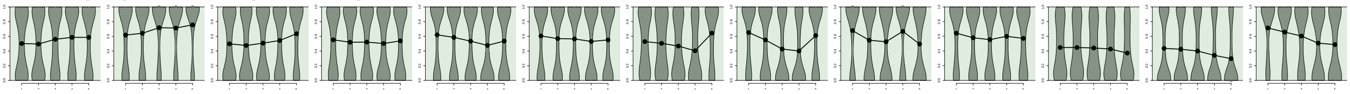

RATIO DOINTRON EXON LENGTH

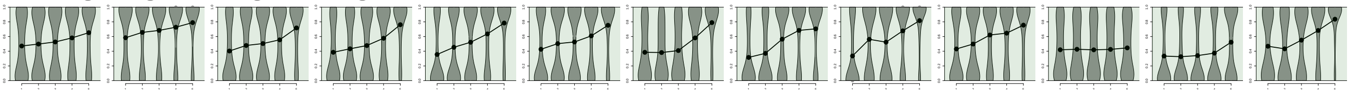

RATIO UPINTLEN DOINTLEN

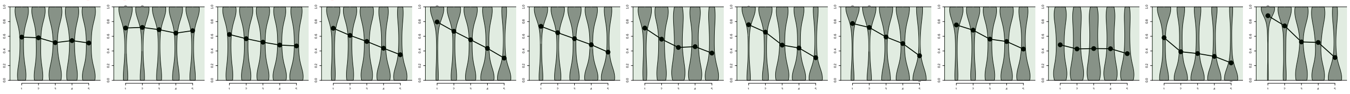

EXON GCC

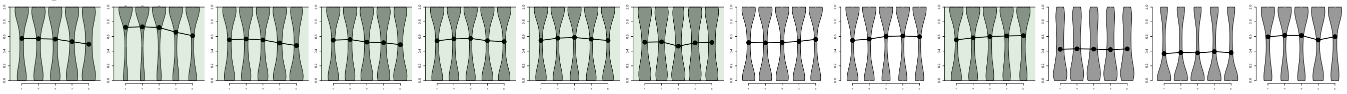

UPINTRON GCC

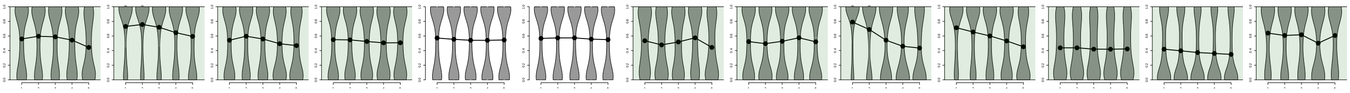

UPEXON GCC

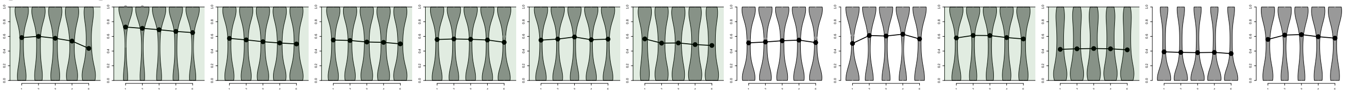

DOINTRON GCC

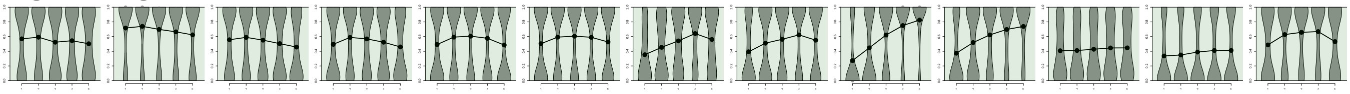

DOEXON GCC

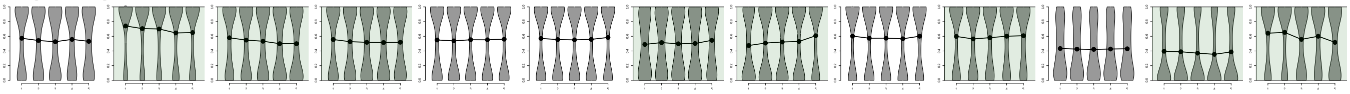

RATIO UPEXON EXON GCC

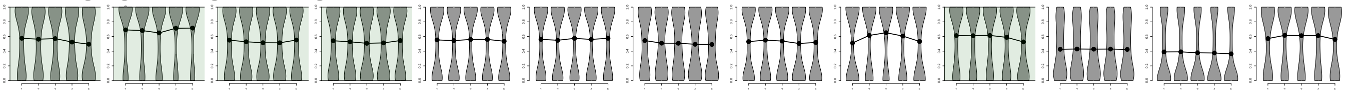

RATIO UPINTRON EXON GCC

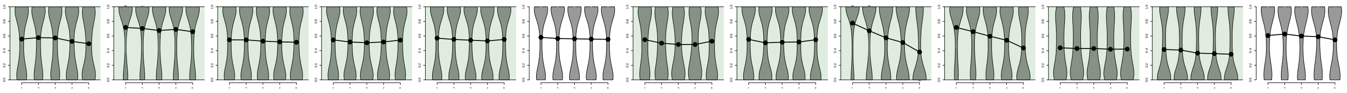

RATIO DOINTRON EXON GCC

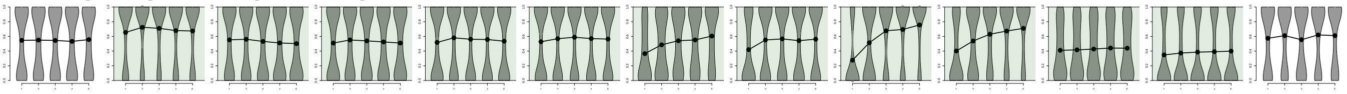

RATIO DOEXON EXON GCC

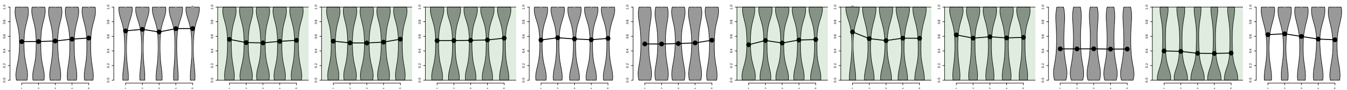

SF1 HIGHESTSCORE 3SS UPINTRON

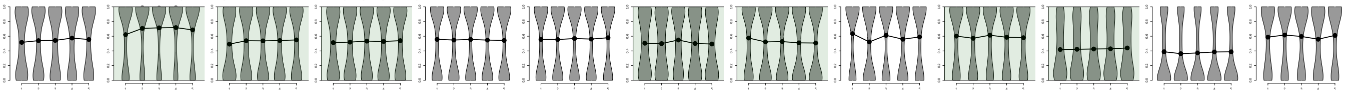

SF1 HIGHESTSCORE 3SS DOINTRON

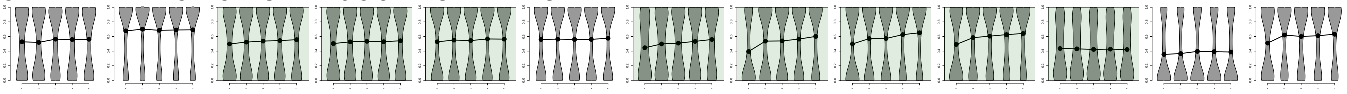

UP 5SS 20INT10EX GCC

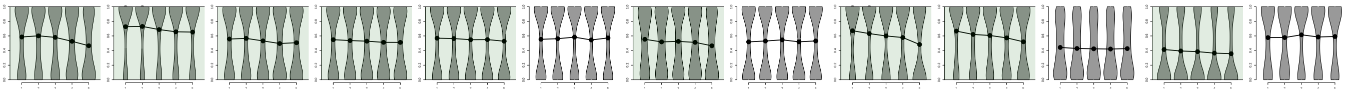

GCC 3SS 20INT10EX

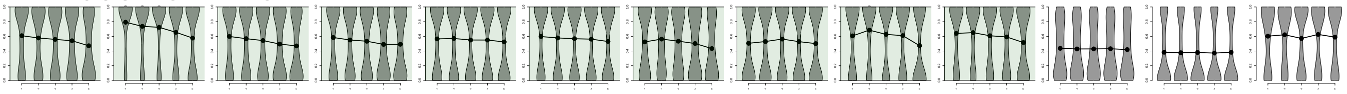

GCC 5SS 20INT10EX

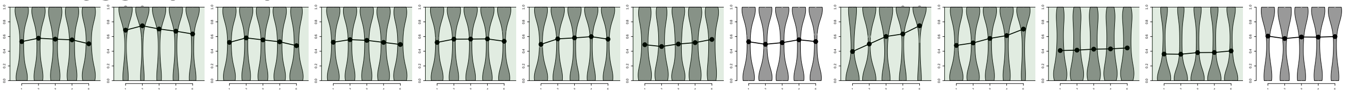

DO 3SS 20INT10EX GCC

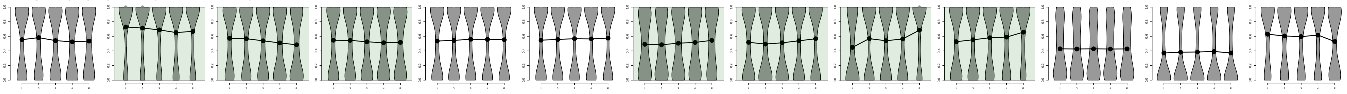

MAXENTSCR HSAMODEL UPSTRM 5SS

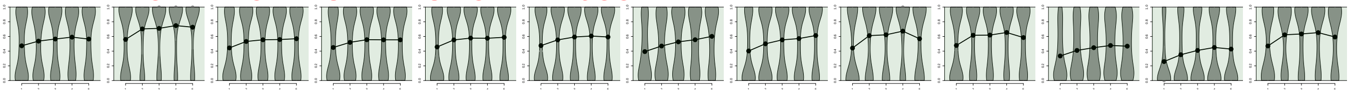

MAXENTSCR HSAMODEL 3SS

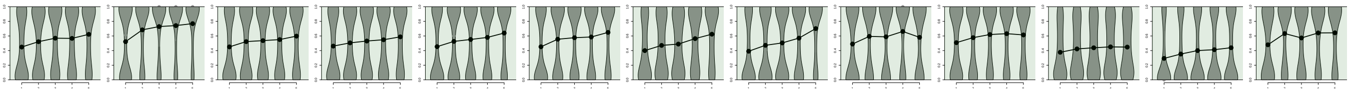

MAXENTSCR HSAMODEL 5SS

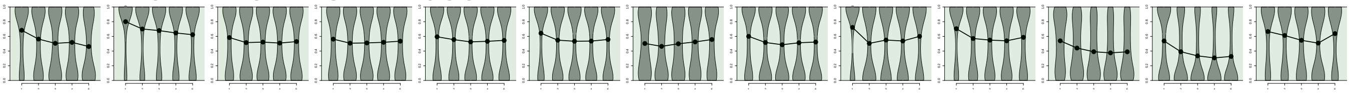

MAXENTSCR HSAMODEL DOWNSTRM 3SS

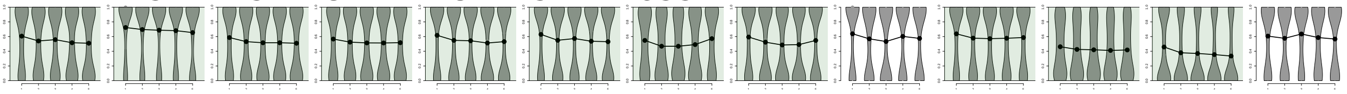

DIST FROM MAXBP TO 3SS UPINTRON

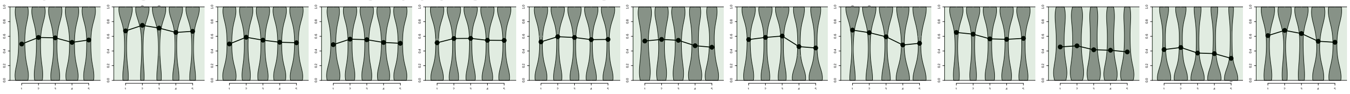

SCORE FOR MAXBP SEQ UPINTRON

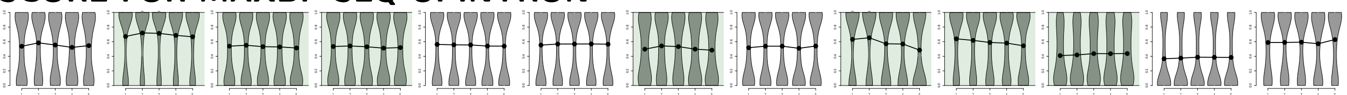

PYRIMIDINECONT MAXBP UPINTRON

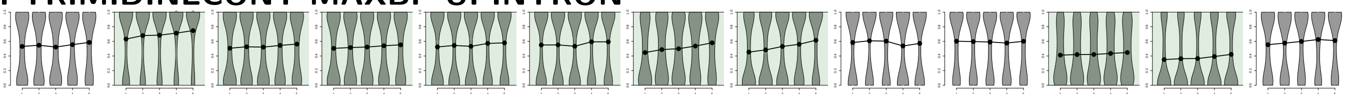

POLYPYRITRAC OFFSET MAXBP UPINTRON

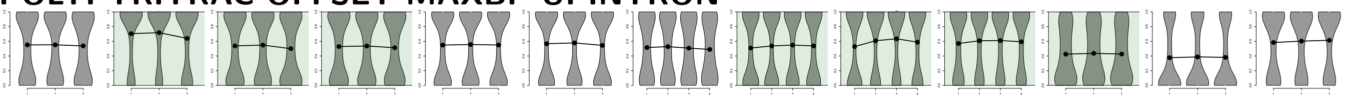

POLYPYRITRAC LEN MAXBP UPINTRON

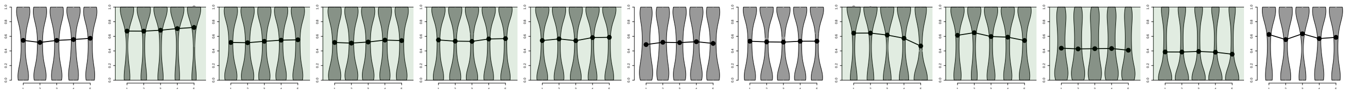

POLYPYRITRAC SCORE MAXBP UPINTRON

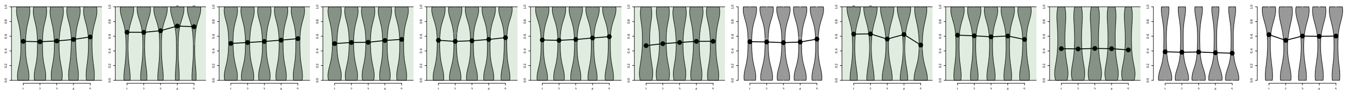

BPSCORE MAXBP UPINTRON

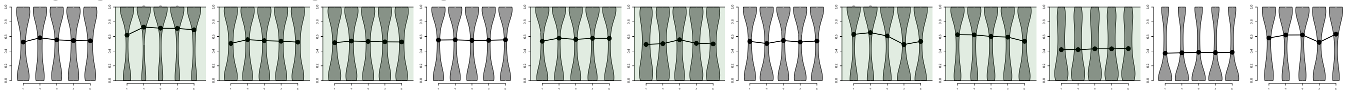

NUM PREDICTED BPS UPINTRON

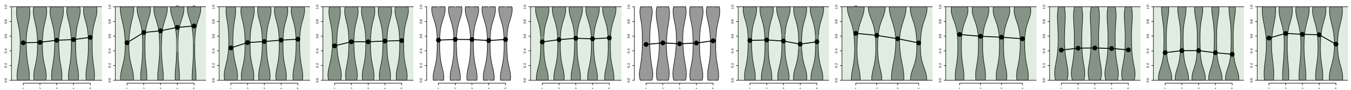

MEDIAN DIST FROM BP TO 3SS UPINTRON

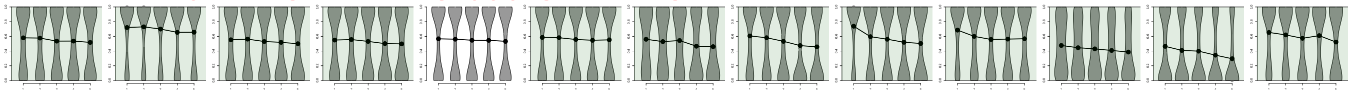

MEDIAN SCORE FOR BPSEQ UPINTRON

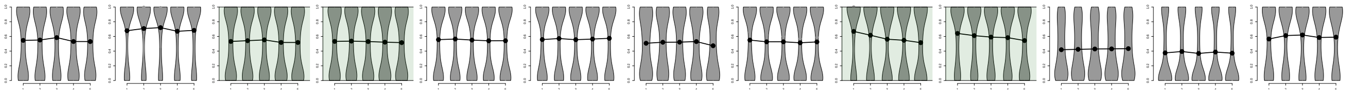

MEDIAN PYRIMIDINECONT UPINTRON

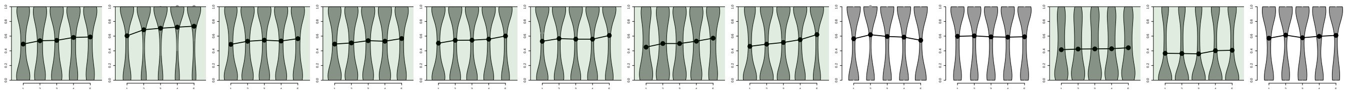

MEDIAN POLYPYRITRAC OFFSET UPINTRON

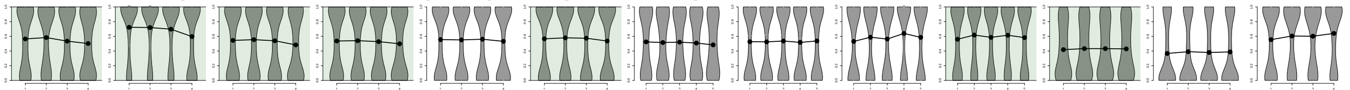

MEDIAN POLYPYRITRAC LEN UPINTRON

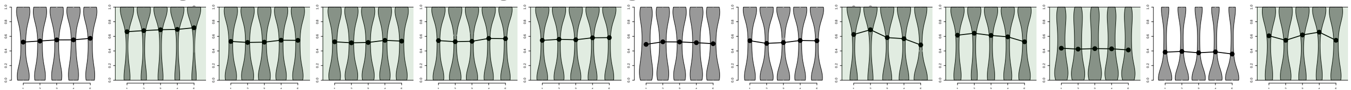

MEDIAN POLYPYRITRAC SCORE UPINTRON

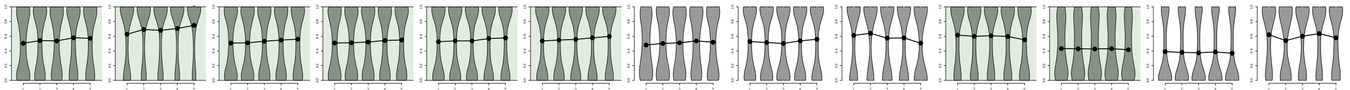

MEDIAN BPSCORE UPINTRON

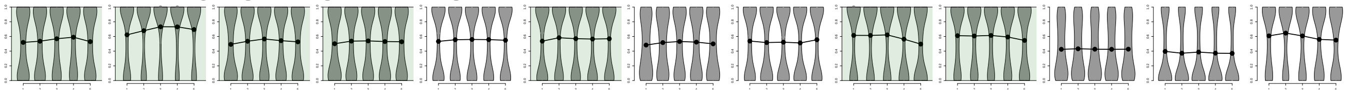

DIST FROM MAXBP TO 3SS DOINTRON

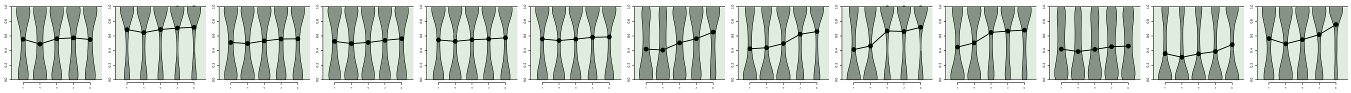

SCORE FOR MAXBP SEQ DOINTRON

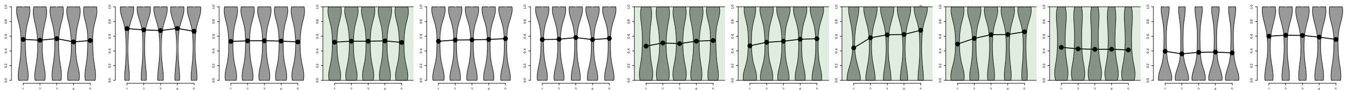

PYRIMIDINECONT MAXBP DOINTRON

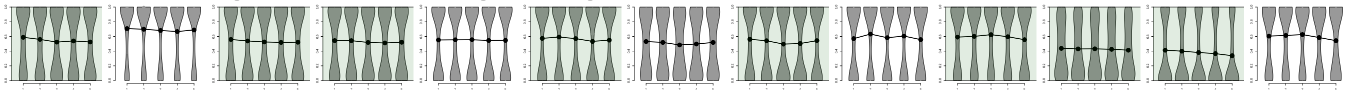

POLYPYRITRAC OFFSET MAXBP DOINTRON

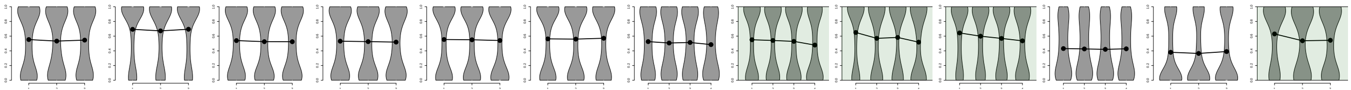

POLYPYRITRAC LEN MAXBP DOINTRON

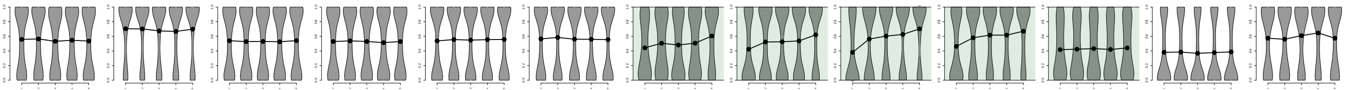

POLYPYRITRAC SCORE MAXBP DOINTRON

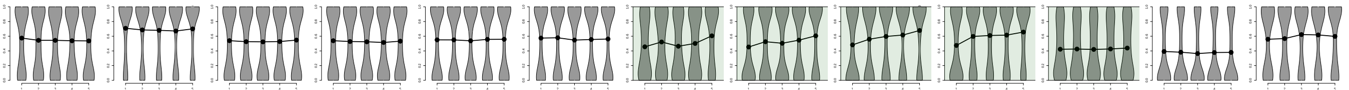

BPSCORE MAXBP DOINTRON

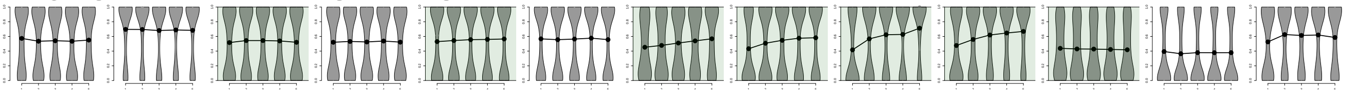

NUM PREDICTED BPS DOINTRON

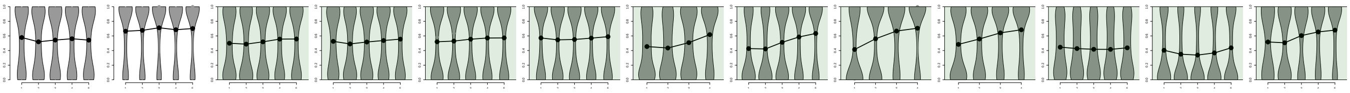

MEDIAN DIST FROM BP TO 3SS DOINTRON

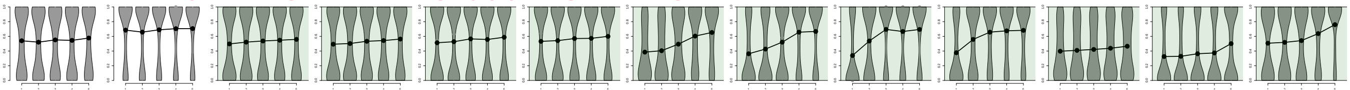

MEDIAN SCORE FOR BPSEQ DOINTRON

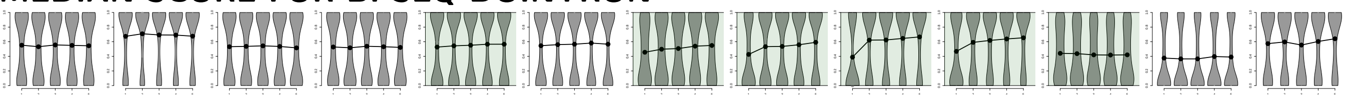

MEDIAN PYRIMIDINECONT DOINTRON

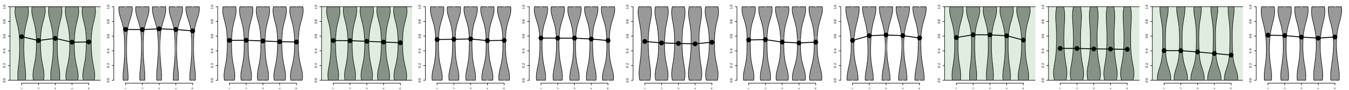

MEDIAN POLYPYRITRAC OFFSET DOINTRON

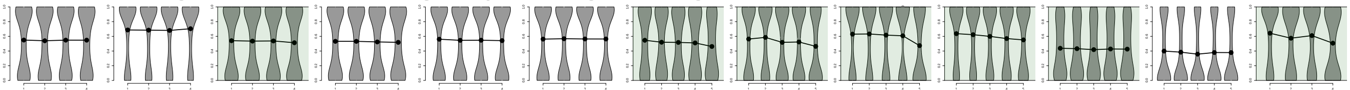

MEDIAN POLYPYRITRAC LEN DOINTRON

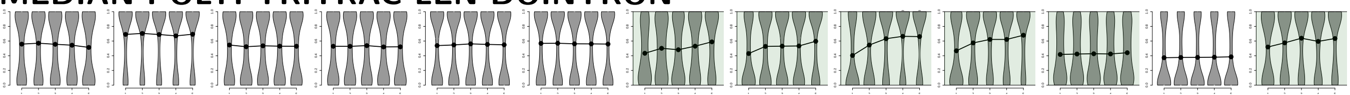

MEDIAN POLYPYRITRAC SCORE DOINTRON

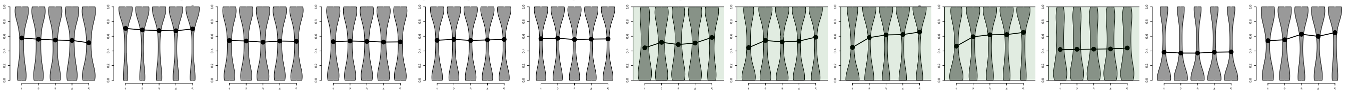

MEDIAN BPSCORE DOINTRON

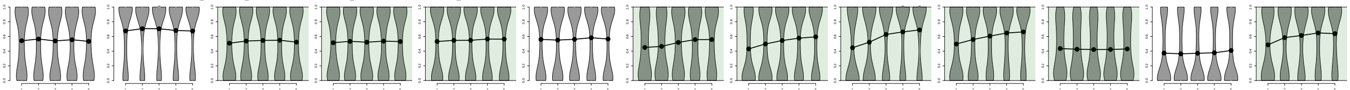

MEDIAN TR LENGTH

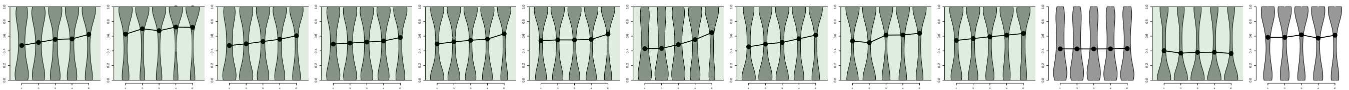

MEDIAN EXON NUMBER

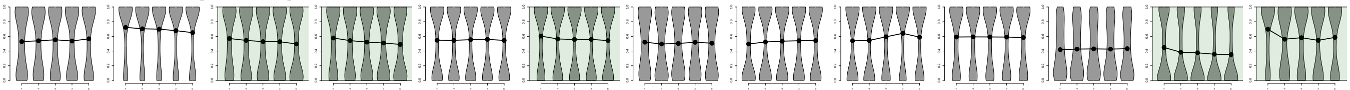

EXON MEDIANRANK

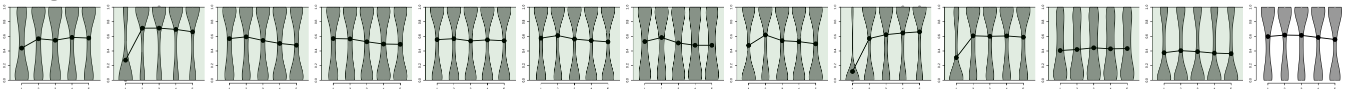

EXON MEDIANRELATIVERANK

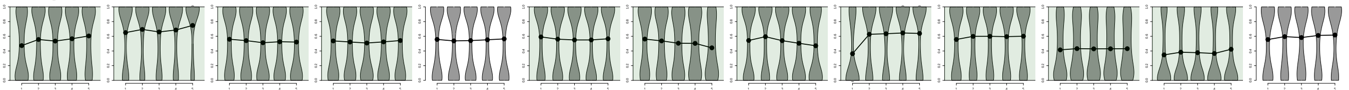

EXON MEDIANRELATIVERANK 3BINS

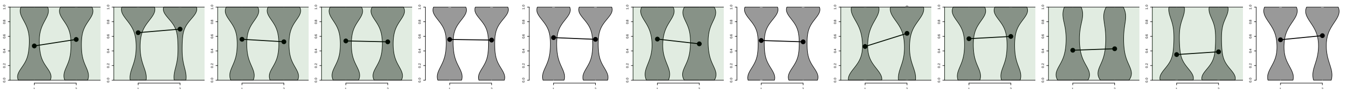

EXON MEDIANRELATIVERANK 5BINS

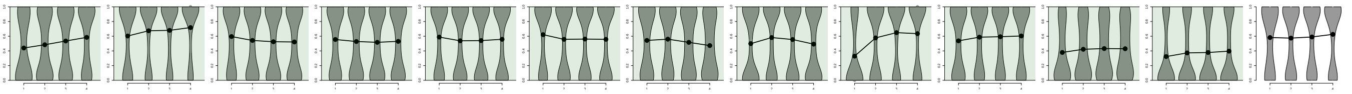

EXON MEDIANRELATIVERANK 10BINS

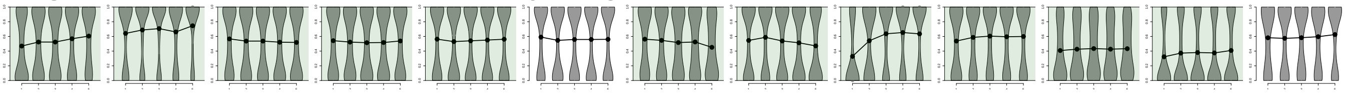

PROP FIRST EXON

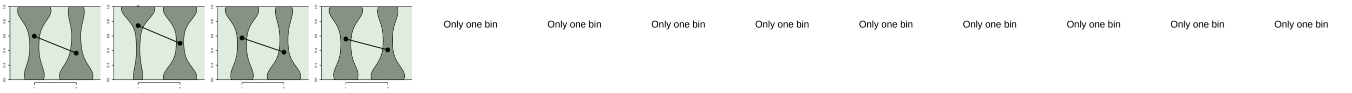

PROP LAST EXON

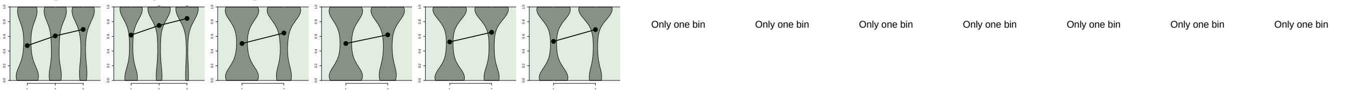

PROP INTERNAL EXON

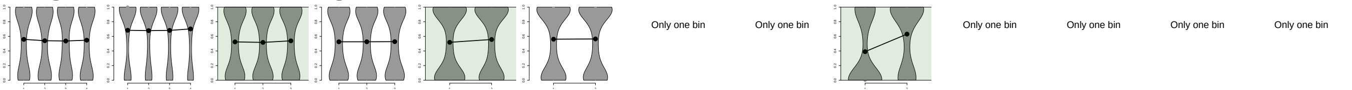

PROP EXON IN UTR

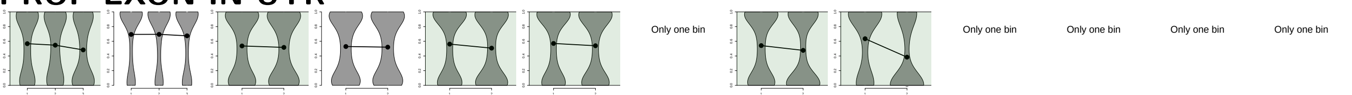

EXON COOCCURS WITH OTHER EXONS

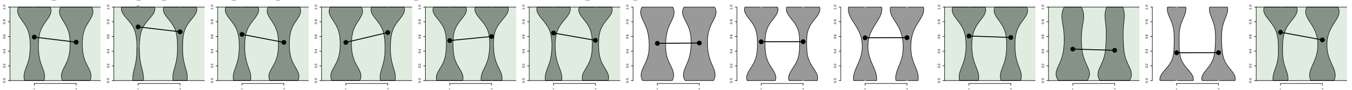

MEDIAN EXON EXON COOCCURRENCE NUMBER OVER ALL TRS

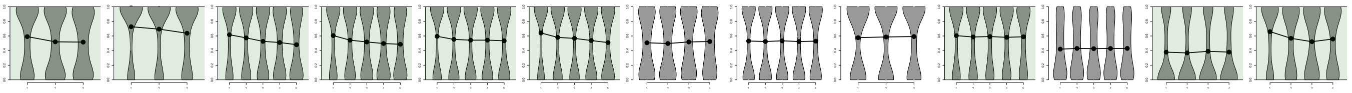

Supplement: gkad244_Supplemental_Files [file gkad244_supplemental_files.zip › Supplementary_File_1-R1.pdf]
