# Supplementary material for: Insplico: effective computational tool for studying splicing order of adjacent introns genome-wide with short and long RNA-seq reads
Source: Nucleic Acids Res. 2023 Apr 7;51(10):e56. doi: 10.1093/nar/gkad244 (PMC10250204; doi:10.1093/nar/gkad244)
Supplement: gkad244_Supplemental_Files [file gkad244_supplemental_files.zip › Supplementary_File_3-R1.pdf]

Comparison of exons grouped into: ctr, srrm4-upfi, srrm4-dofi

June 10, 2022
Matt version 1.3.0

Contents

1 Infos **4**

2 Warning: Please read this note carefully **4**

3 Notes for publishing results **4**

4 Data sets **5**

5 Overview: Features with statistically significant differences (p-val ≤ 0.05) **6**

6 Details: Box plots and statistical assessments for all features **17**

6.1 EXON LENGTH . . . 17
6.2 UPEXON MEDIANLENGTH . . . 18
6.3 DOEXON MEDIANLENGTH . . . 19
6.4 RATIO UPEXON EXON LENGTH . . . 20
6.5 RATIO DOEXON EXON LENGTH . . . 21
6.6 UPINTRON MEDIANLENGTH . . . 22
6.7 DOINTRON MEDIANLENGTH . . . 23
6.8 RATIO UPINTRON EXON LENGTH . . . 24
6.9 RATIO DOINTRON EXON LENGTH . . . 25
6.10 EXON GCC . . . 26
6.11 UPINTRON GCC . . . 27
6.12 UPEXON GCC . . . 28
6.13 DOINTRON GCC . . . 29
6.14 DOEXON GCC . . . 30
6.15 RATIO UPEXON EXON GCC . . . 31

6.16 RATIO UPINTRON EXON GCC . . . 32
6.17 RATIO DOINTRON EXON GCC . . . 33
6.18 RATIO DOEXON EXON GCC . . . 34
6.19 SF1 HIGHESTSCORE 3SS UPINTRON . . . 35
6.20 SF1 HIGHESTSCORE 3SS DOINTRON . . . 36
6.21 UP 5SS 20INT10EX GCC . . . 37
6.22 GCC 3SS 20INT10EX . . . 38
6.23 GCC 5SS 20INT10EX . . . 39
6.24 DO 3SS 20INT10EX GCC . . . 40
6.25 MAXENTSCR HSAMODEL UPSTRM 5SS . . . 41
6.26 MAXENTSCR HSAMODEL 3SS . . . 42
6.27 MAXENTSCR HSAMODEL 5SS . . . 43
6.28 MAXENTSCR HSAMODEL DOWNSTRM 3SS . . . 44
6.29 DIST FROM MAXBP TO 3SS UPINTRON . . . 45
6.30 SCORE FOR MAXBP SEQ UPINTRON . . . 46
6.31 PYRIMIDINECONT MAXBP UPINTRON . . . 47
6.32 POLYPYRITRAC OFFSET MAXBP UPINTRON . . . 48
6.33 POLYPYRITRAC LEN MAXBP UPINTRON . . . 49
6.34 POLYPYRITRAC SCORE MAXBP UPINTRON . . . 50
6.35 BPSCORE MAXBP UPINTRON . . . 51
6.36 NUM PREDICTED BPS UPINTRON . . . 52
6.37 MEDIAN DIST FROM BP TO 3SS UPINTRON . . . 53
6.38 MEDIAN SCORE FOR BPSEQ UPINTRON . . . 54
6.39 MEDIAN PYRIMIDINECONT UPINTRON . . . 55
6.40 MEDIAN POLYPYRITRAC OFFSET UPINTRON . . . 56
6.41 MEDIAN POLYPYRITRAC LEN UPINTRON . . . 57
6.42 MEDIAN POLYPYRITRAC SCORE UPINTRON . . . 58
6.43 MEDIAN BPSCORE UPINTRON . . . 59
6.44 DIST FROM MAXBP TO 3SS DOINTRON . . . 60
6.45 SCORE FOR MAXBP SEQ DOINTRON . . . 61
6.46 PYRIMIDINECONT MAXBP DOINTRON . . . 62
6.47 POLYPYRITRAC OFFSET MAXBP DOINTRON . . . 63
6.48 POLYPYRITRAC LEN MAXBP DOINTRON . . . 64
6.49 POLYPYRITRAC SCORE MAXBP DOINTRON . . . 65
6.50 BPSCORE MAXBP DOINTRON . . . 66
6.51 NUM PREDICTED BPS DOINTRON . . . 67
6.52 MEDIAN DIST FROM BP TO 3SS DOINTRON . . . 68
6.53 MEDIAN SCORE FOR BPSEQ DOINTRON . . . 69
6.54 MEDIAN PYRIMIDINECONT DOINTRON . . . 70
6.55 MEDIAN POLYPYRITRAC OFFSET DOINTRON . . . 71
6.56 MEDIAN POLYPYRITRAC LEN DOINTRON . . . 72
6.57 MEDIAN POLYPYRITRAC SCORE DOINTRON . . . 73
6.58 MEDIAN BPSCORE DOINTRON . . . 74
6.59 MEDIAN TR LENGTH . . . 75

6.60 MEDIAN EXON NUMBER 76
6.61 EXON MEDIANRELATIVERANK 77
6.62 EXON MEDIANRELATIVERANK 3BINS 78
6.63 EXON MEDIANRELATIVERANK 5BINS 79
6.64 EXON MEDIANRELATIVERANK 10BINS 80
6.65 NTRS ALL FOR GENE 81
6.66 PROP FIRST EXON 82
6.67 PROP LAST EXON 83
6.68 PROP INTERNAL EXON 84
6.69 PROP EXON IN UTR 85

1 Infos

Visualizations of exon features for different groups of exons. Each exon occurs in exactly one gene, but might occur in several transcripts of that gene. Hence, for some features like the exon length, there is exactly one value for each exon. For other features, e.g., length of the up-stream exon(s), which could be different in different transcripts, there might be several values for each exon. Consequently, in the latter cases, the median of these value gets reported.

2 Warning: Please read this note carefully

Please keep in mind that some features might affect other features. Especially: all branch-point features get extracted from sub-sequences of introns, by standard the last 150 nt at the 3' end of each intron (if you haven't changed this) always neglecting the first 20 nt at their 5' end. If introns of one set are especially short, i.e., many are shorter then these 150 nt, then the shorter intron length might affect branch-point features. For example, there might be less branch points found in shorter introns or their distance to the 3' intron ends might be generally shorter simply because of their shorter intron length.

3 Notes for publishing results

The Matt paper: *Matt: Unix tools for alternative splicing analysis, A. Gohr, M. Irimia, Bioinformatics, 2018, bty606, DOI: 10.1093/bioinformatics/bty606*
When publishing results wrt. splice site strengths which you determined for your data using matt, please cite: *Maximum entropy modeling of short sequence motifs with applications to RNA splicing signals, Yeo et al., 2003, DOI: 10.1089/1066527041410418*
When publishing results wrt. branch point features which you determined for your data with matt, please cite: *Genome-wide association between branch point properties and alternative splicing, Corvelo et al., 2010, DOI: 10.1371/journal.pcbi.1001016*
When publishing results with respect to the binding strength of the human Sf1 splicing factor, you might refert to where the Sf1 binding motif comes from: *Analysis of in situ pre-mRNA targets of human splicing factor SF1 reveals a function in alternative splicing, Margherita Corioni, Nicolas Antih, Goranka Tanackovic, Mihaela Zavolan, and Angela Kramer, 2011, DOI: 10.1093/nar/gkq1042*
The Sf1 binding motif is described in supplement, page 13, table S2: Weight matrix of the binding specificity of SF1.

4 Data sets

Input file:
`srrm4_exons_dpsi15-MI-v2.tab`
Selection criteria for defining exons groups:
`ctr` : having value `ctr` in column `GROUP`
`srrm4_upfi` : having value `srrm4_upfi` in column `GROUP`
`srrm4_dofi` : having value `srrm4_dofi` in column `GROUP`
Exon duplicates removal: yes
Numbers of exons per group before / after neglecting exons which were not found in GTF file (gene annotation). For the comparisons only exons which were found in the gene annotation are used. These numbers might change slightly for each feature if NAs occur.
`ctr`: 120 / 115
`srrm4_upfi`: 24 / 24
`srrm4_dofi`: 55 / 55

5 Overview: Features with statistically significant differences (p-val ≤ 0.05)

MAXENTSCR HSAMODEL 3SS

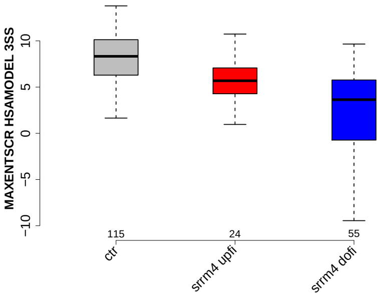

MEDIAN TR LENGTH

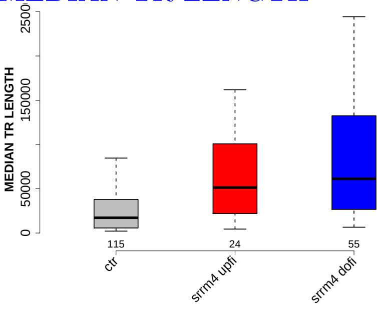

EXON LENGTH

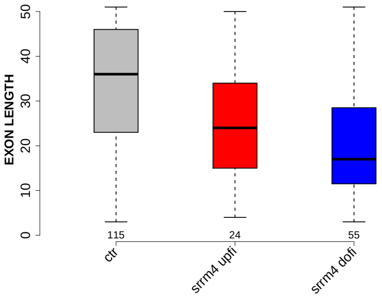

RATIO UPINTRON EXON LENGTH

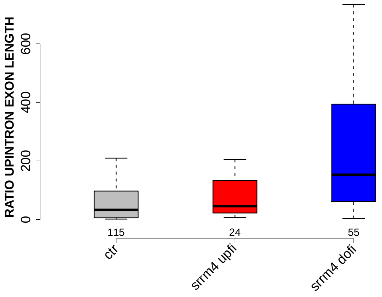

UPINTRON GCC

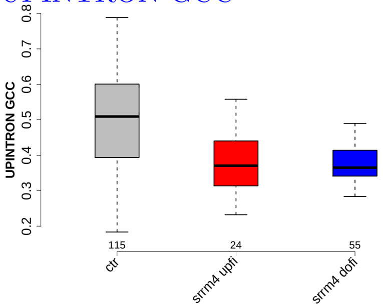

RATIO DOEXON EXON LENGTH

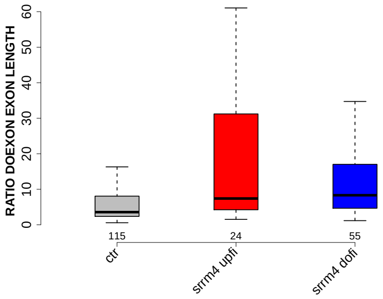

SF1 HIGHESTSCORE 3SS UPINTRON

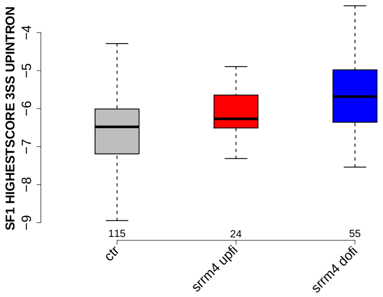

RATIO UPEXON EXON LENGTH

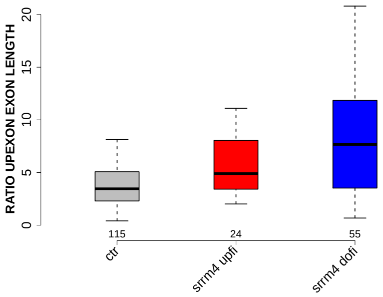

PROP INTERNAL EXON

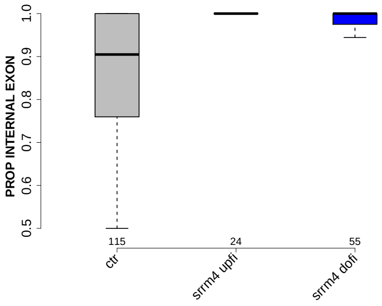

PROP EXON IN UTR

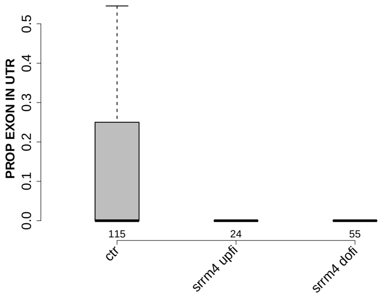

MEDIAN EXON NUMBER

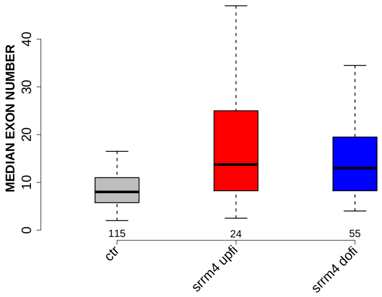

DOINTRON GCC

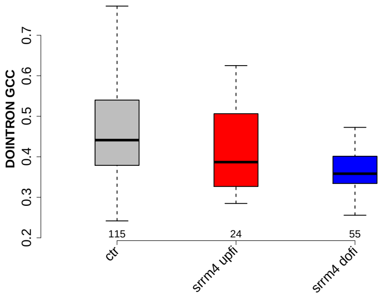

UPEXON GCC

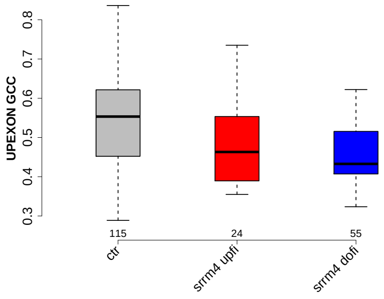

MEDIAN POLYPYRITRAC SCORE UPINTRON

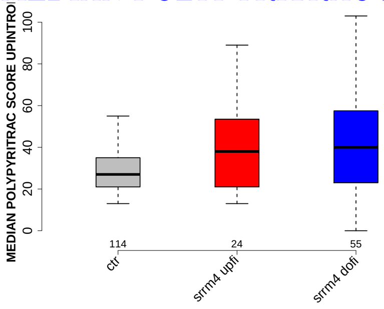

UPINTRON MEDIANLENGTH

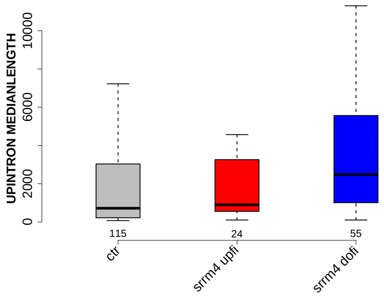

UP 5SS 20INT10EX GCC

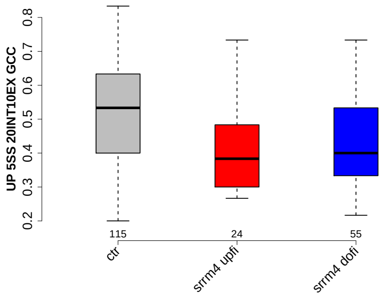

MEDIAN POLYPYRITRAC OFFSET UPINTRON

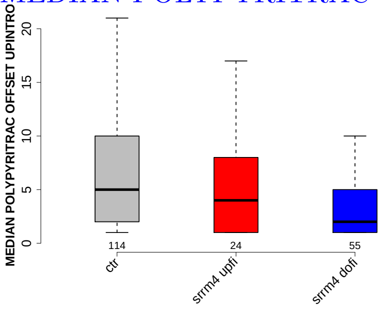

PROP LAST EXON

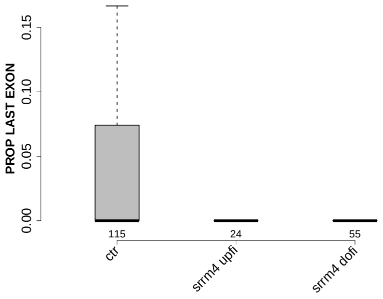

NUM PREDICTED BPS UPINTRON

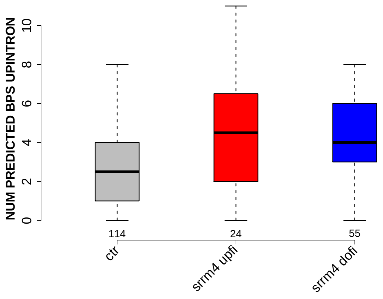

GCC 5SS 20INT10EX

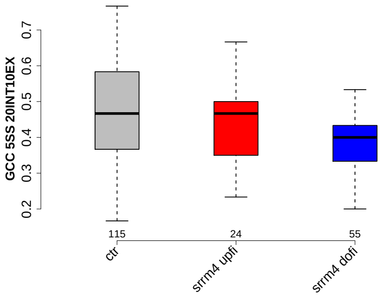

MEDIAN POLYPYRITRAC LEN UPINTRON

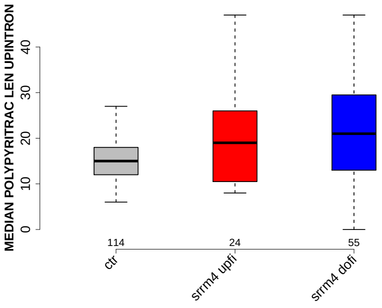

DOEXON GCC

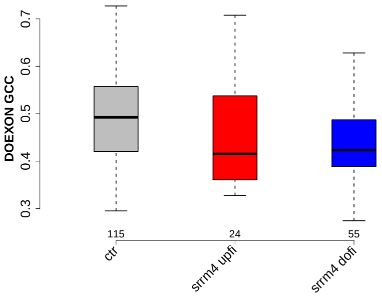

PROP FIRST EXON

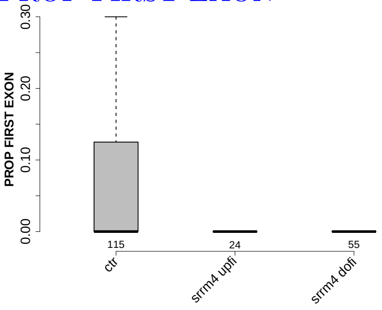

RATIO UPINTRON EXON GCC

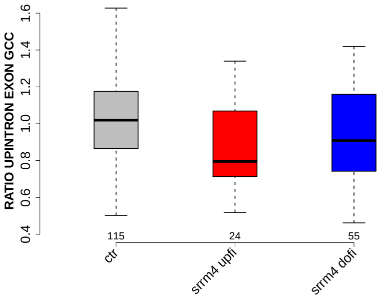

EXON MEDIANRELATIVERANK 5BINS

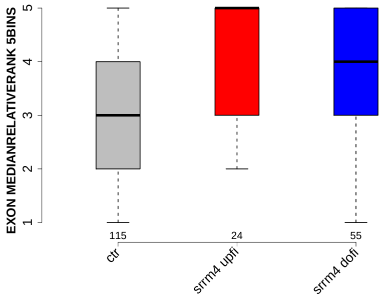

EXON MEDIANRELATIVERANK 10BINS

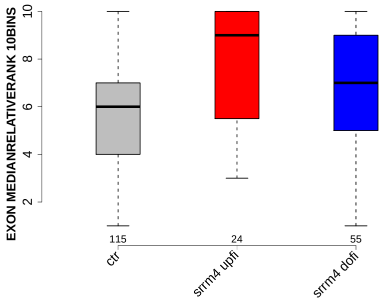

EXON MEDIANRELATIVERANK

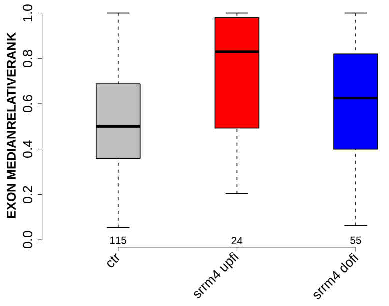

POLYPYRITRAC LEN MAXBP UPINTRON

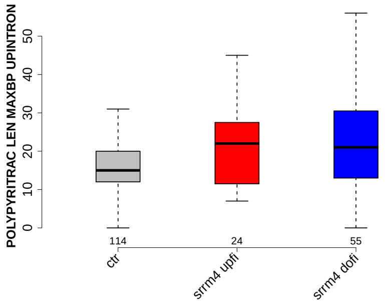

POLYPYRITRAC SCORE MAXBP UPINTRON

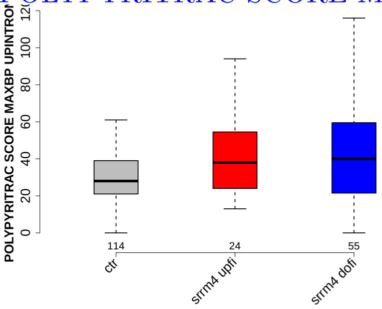

MAXENTSCR HSAMODEL 5SS

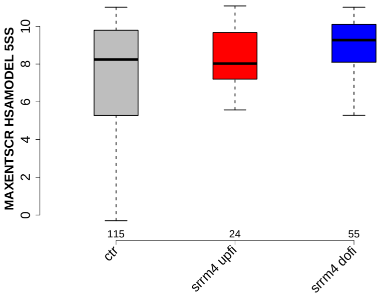

MAXENTSCR HSAMODEL UPSTRM 5SS

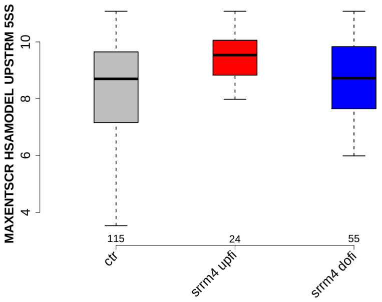

MEDIAN PYRIMIDINECONT UPINTRON

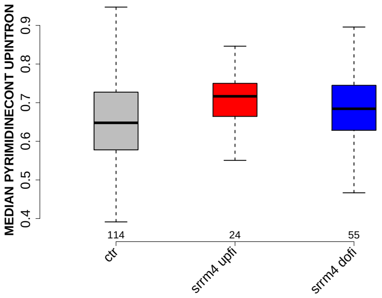

RATIO DOINTRON EXON LENGTH

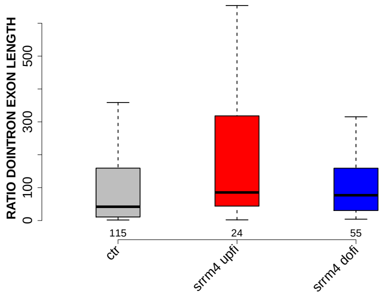

POLYPYRITRAC OFFSET MAXBP UPINTRON

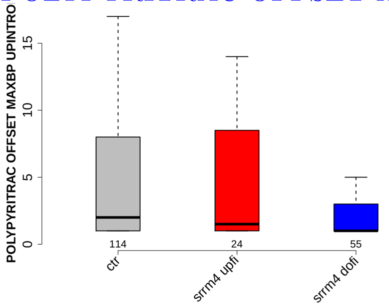

EXON GCC

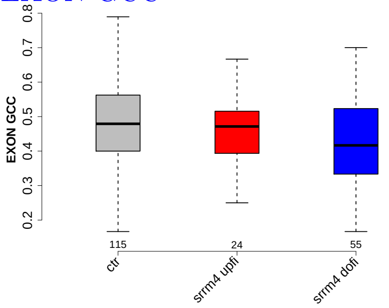

NUM PREDICTED BPS DOINTRON

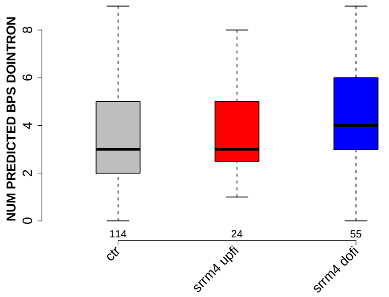

BPSCORE MAXBP UPINTRON

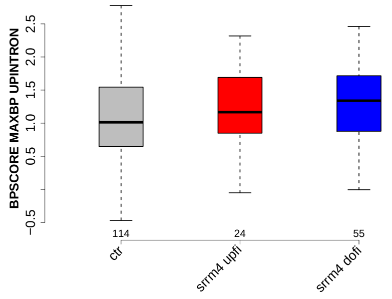

EXON MEDIANRELATIVERANK 3BINS

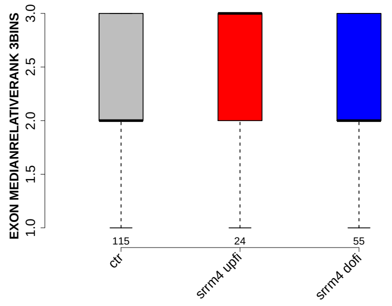

MEDIAN BPSCORE UPINTRON

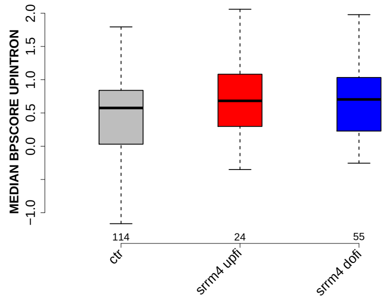

DOEXON MEDIANLENGTH

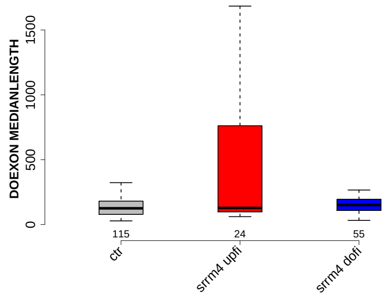

DOINTRON MEDIANLENGTH

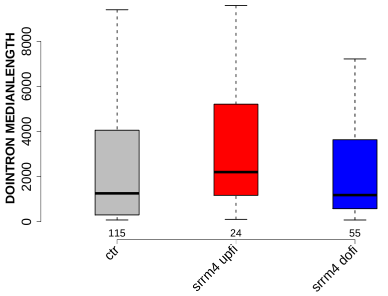

MEDIAN BPSCORE DOINTRON

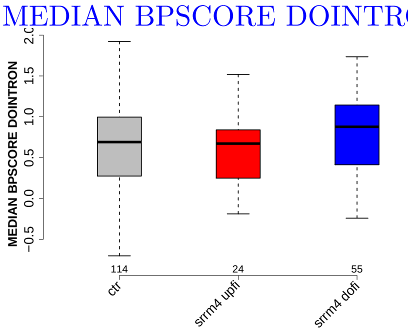

6 Details: Box plots and statistical assessments for all features

6.1 EXON LENGTH

Back to: Overview | ToC

Meaning:

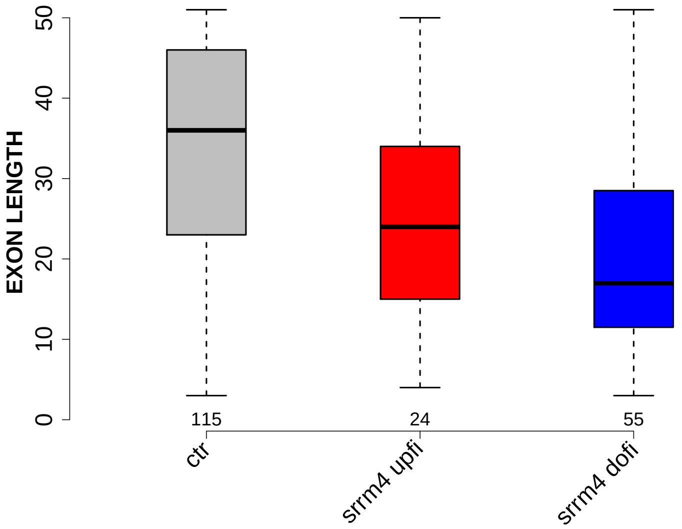

Significant results from Mann-Whitney U test:

- `ctr` vs `srrm4_upfi` : 0.0159894
  mean: 33.1304 > 25.3333 , median: 36 > 24
- `ctr` vs `srrm4_dofi` : 2.8587e-07
  mean: 33.1304 > 20.5091 , median: 36 > 17

6.2 UPEXON MEDIANLENGTH

Back to: Overview | ToC

Meaning: median length of up-stream exon

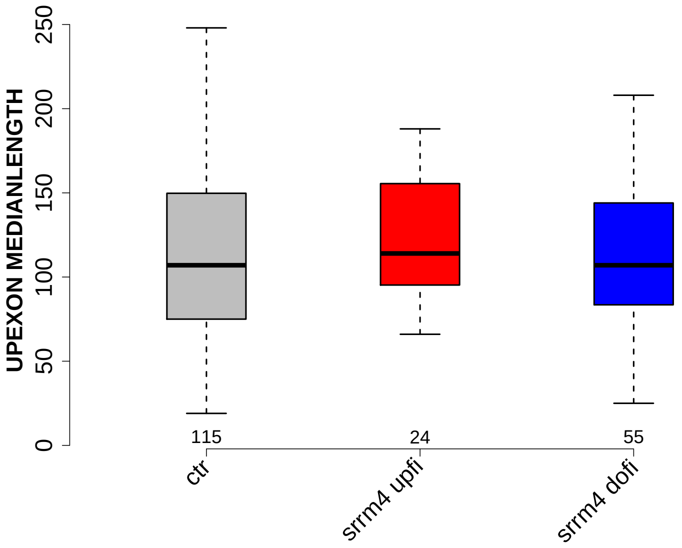

Significant results from Mann-Whitney U test:

- none

6.3 DOEXON MEDIANLENGTH

Back to: Overview | ToC

Meaning: median length of down-stream exon

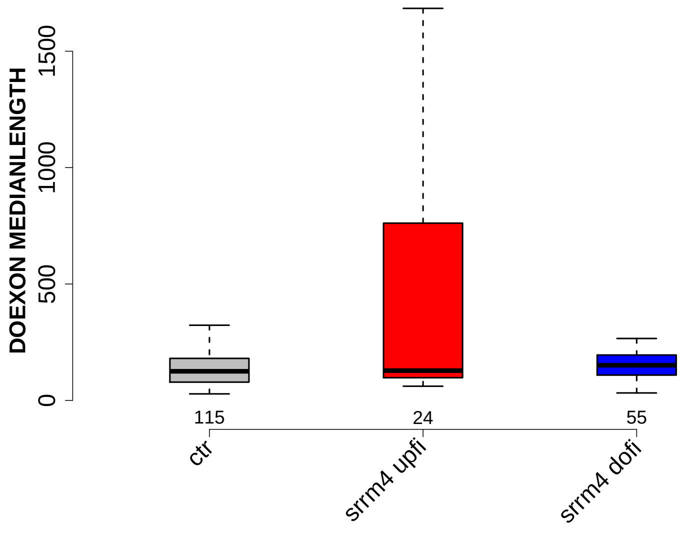

Significant results from Mann-Whitney U test:

- `ctr` vs `srrm4_dofi` : 0.0334315
  mean: 221.1174 > 209.1636 , median: 125 < 151

6.4 RATIO UPEXON EXON LENGTH

Back to: Overview | ToC

Meaning: median up-stream exon length / exon length

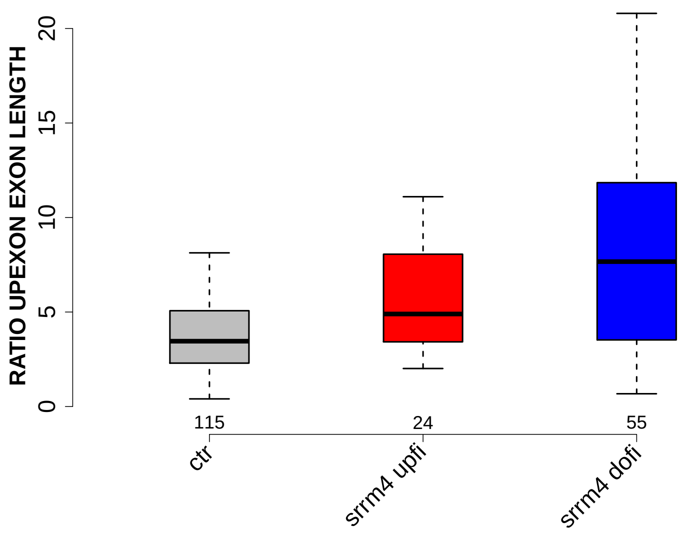

Significant results from Mann-Whitney U test:

- `ctr` vs `srrm4_upfi` : 0.00699159
  mean: 5.6095 < 7.4116 , median: 3.4565 < 4.8958
- `ctr` vs `srrm4_dofi` : 8.51637e-06
  mean: 5.6095 < 10.0838 , median: 3.4565 < 7.6667

6.5 RATIO DOEXON EXON LENGTH

Back to: Overview | ToC

Meaning: median down-stream exon length / exon length

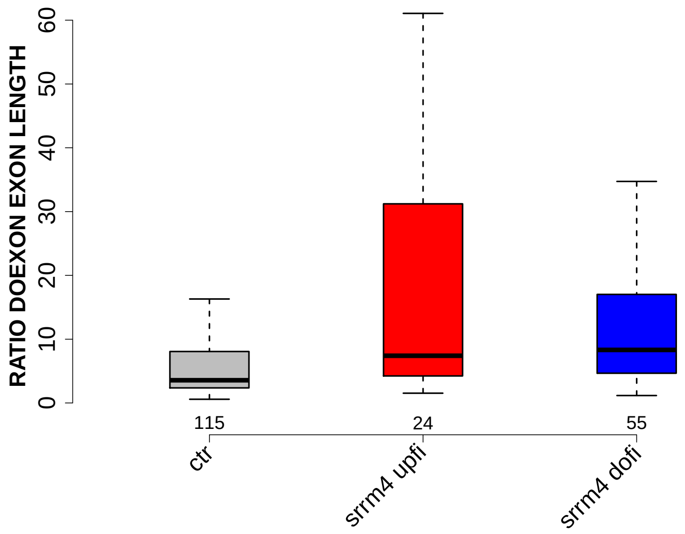

Significant results from Mann-Whitney U test:

- `ctr` vs `srrm4_upfi` : 0.00182094
  mean: 9.3354 < 39.2443 , median: 3.5625 < 7.4028
- `ctr` vs `srrm4_dofi` : 4.46799e-06
  mean: 9.3354 < 15.9763 , median: 3.5625 < 8.32

6.6 UPINTRON MEDIANLENGTH

Back to: Overview | ToC

Meaning: median length of up-stream introns

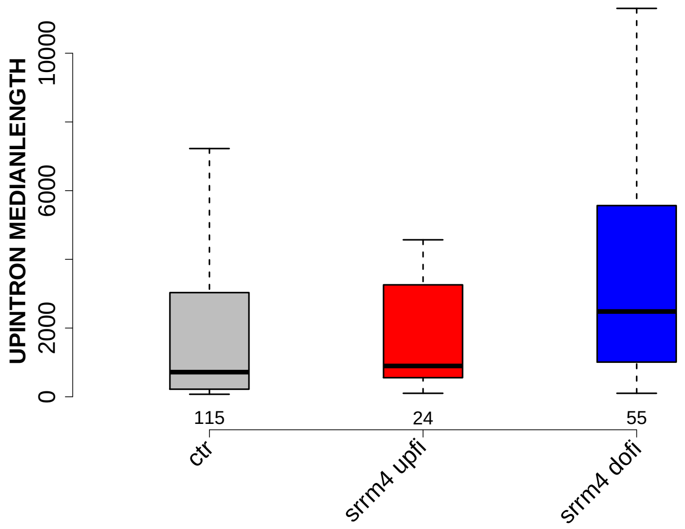

Significant results from Mann-Whitney U test:

- `ctr` vs `srrm4_dofi` : 0.000299529
  mean: 4293.1478 < 4827.0091 , median: 717 < 2482

6.7 DOINTRON MEDIANLENGTH

Back to: Overview | ToC

Meaning: median length of down-stream introns

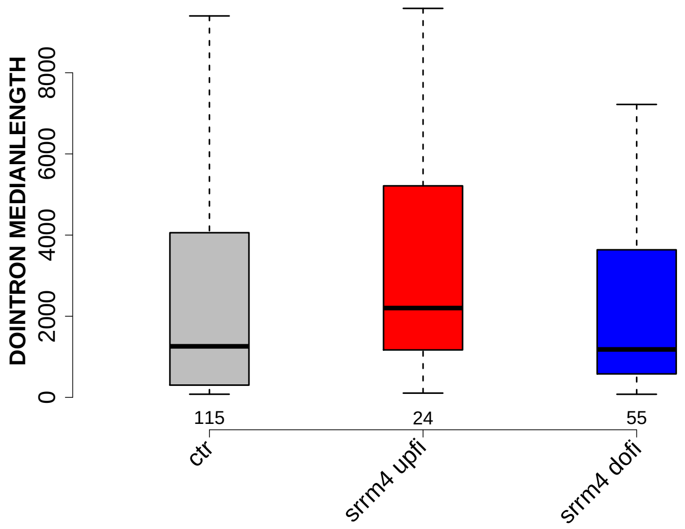

Significant results from Mann-Whitney U test:

- ctr vs srrm4_upfi : 0.0436588
  mean: 4657.3217 $<$ 5738.625 , median: 1258 $<$ 2201.5
- srrm4_upfi vs srrm4_dofi : 0.0479923
  mean: 5738.625 $>$ 2441.4727 , median: 2201.5 $>$ 1182

6.8 RATIO UPINTRON EXON LENGTH

Back to: Overview | ToC

Meaning: median up-stream intron length / exon length

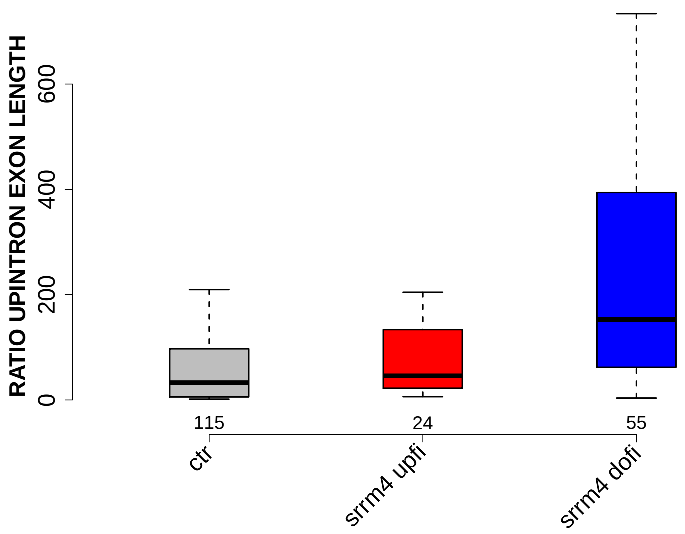

Significant results from Mann-Whitney U test:

- `ctr` vs `srrm4_dofi` : 7.1816e-07
  mean: 160.6581 $<$ 289.6254 , median: 32.875 $<$ 152.6667
- `srrm4_upfi` vs `srrm4_dofi` : 0.0116989
  mean: 217.7701 $<$ 289.6254 , median: 45.8611 $<$ 152.6667

6.9 RATIO DOINTRON EXON LENGTH

Back to: Overview | ToC

Meaning: median down-stream intron length / exon length

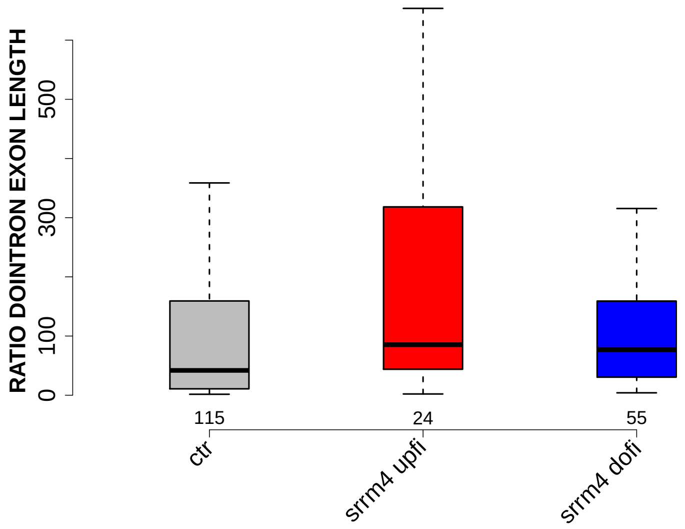

Significant results from Mann-Whitney U test:

- `ctr` vs `srrm4_upfi` : 0.011136
  mean: 233.2837 < 453.3498 , median: 41.9333 < 85.4111
- `ctr` vs `srrm4_dofi` : 0.0241319
  mean: 233.2837 > 170.4348 , median: 41.9333 < 76.9167

6.10 EXON GCC

Back to: Overview | ToC

Meaning: GC content of entire exon sequence

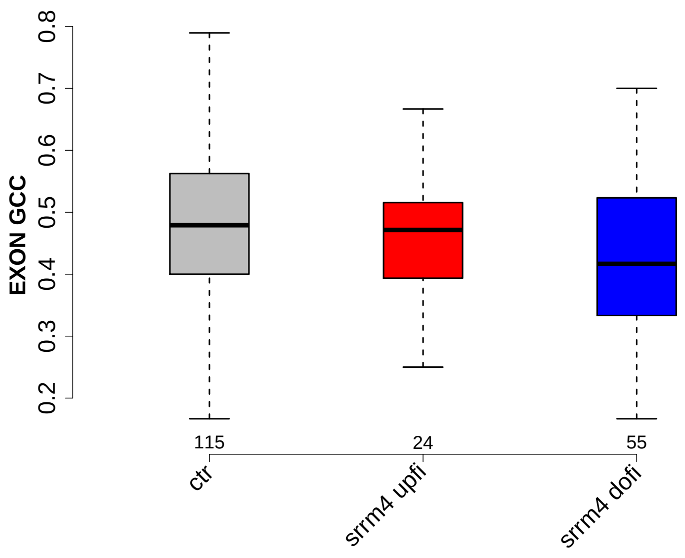

Significant results from Mann-Whitney U test:

- `ctr` vs `srrm4_dofi` : 0.0133733
  mean: 0.482338 > 0.433147 , median: 0.479167 > 0.416667

6.11 UPINTRON GCC

Back to: Overview | ToC

Meaning: GC content of entire up-stream intron sequence

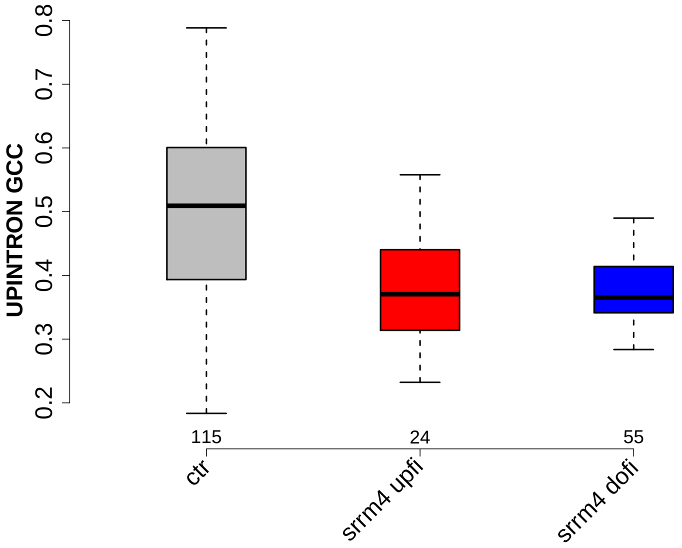

Significant results from Mann-Whitney U test:

- `ctr` vs `srrm4_upfi` : 0.000350309
  mean: 0.49662 > 0.393058 , median: 0.509219 > 0.370542
- `ctr` vs `srrm4_dofi` : 1.00954e-06
  mean: 0.49662 > 0.400146 , median: 0.509219 > 0.365022

6.12 UPEXON GCC

Back to: Overview | ToC

Meaning: GC content of entire up-stream exon sequence

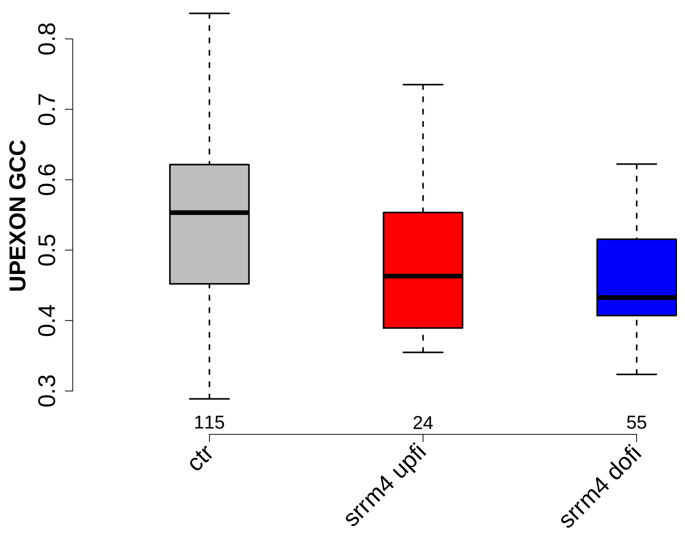

Significant results from Mann-Whitney U test:

- `ctr` vs `srrm4_upfi` : 0.0145383
  mean: 0.544426 > 0.48744 , median: 0.553299 > 0.463063
- `ctr` vs `srrm4_dofi` : 4.59343e-05
  mean: 0.544426 > 0.467907 , median: 0.553299 > 0.432692

6.13 DOINTRON GCC

Back to: Overview | ToC

Meaning: GC content of entire down-stream intron sequence

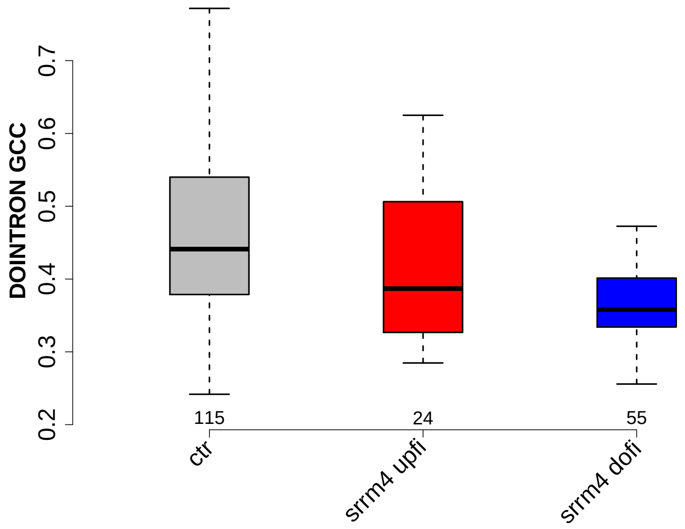

Significant results from Mann-Whitney U test:

- `ctr` vs `srrm4_upfi` : 0.0287194
  mean: 0.460786 > 0.410773 , median: 0.44111 > 0.386832
- `ctr` vs `srrm4_dofi` : 1.73228e-05
  mean: 0.460786 > 0.39575 , median: 0.44111 > 0.358217

6.14 DOEXON GCC

Back to: Overview | ToC

Meaning: GC content of entire down-stream exon sequence

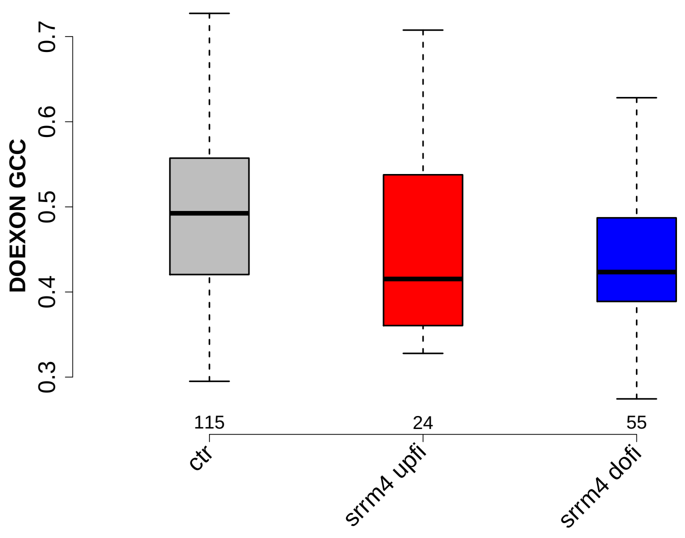

Significant results from Mann-Whitney U test:

- `ctr` vs `srrm4_dofi` : 0.00138524
  mean: 0.492421 > 0.443651 , median: 0.492453 > 0.423423

6.15 RATIO UPEXON EXON GCC

Back to: Overview | ToC

Meaning: UPEXON GCC / EXON GCC

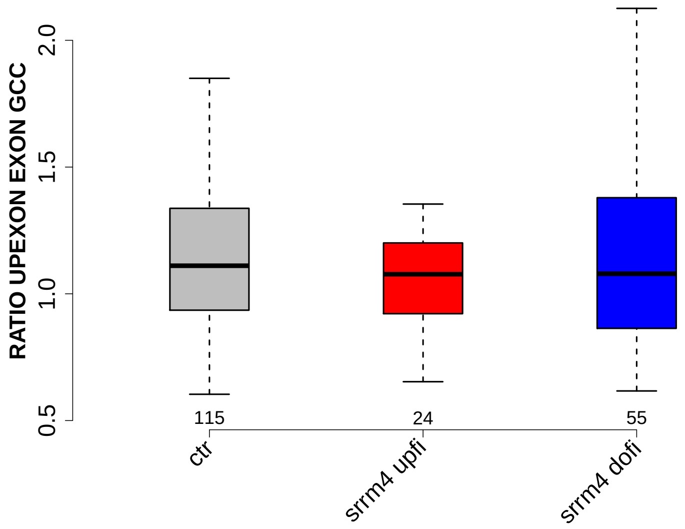

Significant results from Mann-Whitney U test:

- none

6.16 RATIO UPINTRON EXON GCC

Back to: Overview | ToC

Meaning: UPINTRON GCC / EXON GCC

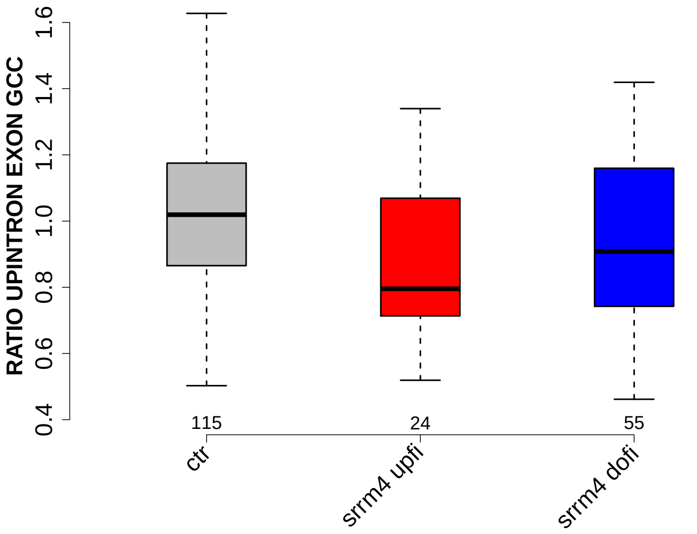

Significant results from Mann-Whitney U test:

- `ctr` vs `srrm4_upfi` : 0.00168796
  mean: 1.0708 > 0.870257 , median: 1.0193 > 0.79553

6.17 RATIO DOINTRON EXON GCC

Back to: Overview | ToC

Meaning: DOINTRON GCC / EXON GCC

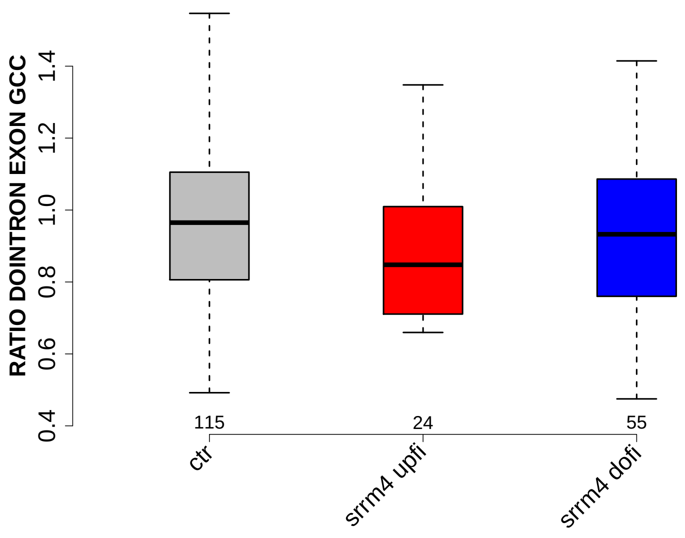

Significant results from Mann-Whitney U test:

- none

6.18 RATIO DOEXON EXON GCC

Back to: Overview | ToC

Meaning: DOEXON GCC / EXON GCC

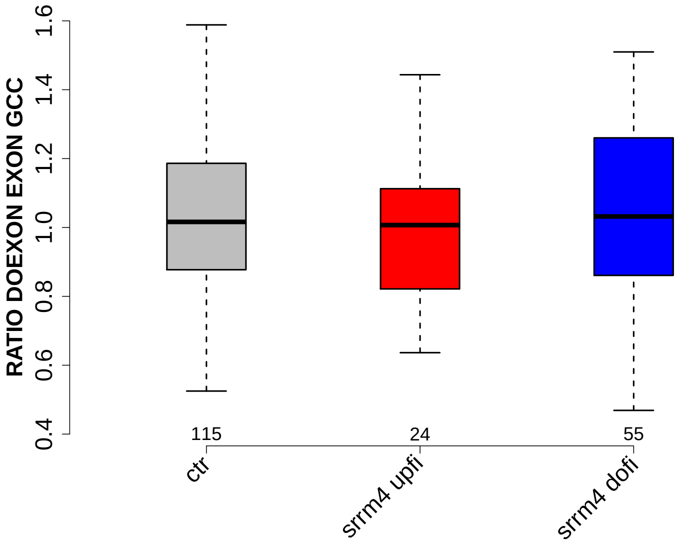

Significant results from Mann-Whitney U test:

- none

6.19 SF1 HIGHESTSCORE 3SS UPINTRON

Back to: Overview | ToC

Meaning: highest score of a SF1 positon weight matrix trained with human data in the last 150 nt 3 prime intron positons of up-stream intron

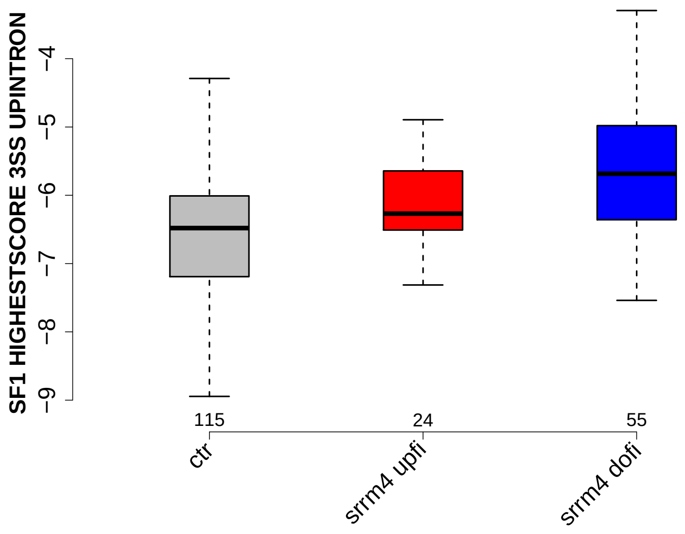

Significant results from Mann-Whitney U test:

- `ctr` vs `srrm4_upfi` : 0.0325769
  mean: -6.52951 < -6.13069 , median: -6.48026 < -6.26872
- `ctr` vs `srrm4_dofi` : 5.8465e-06
  mean: -6.52951 < -5.7077 , median: -6.48026 < -5.6844

6.20 SF1 HIGHESTSCORE 3SS DOINTRON

Back to: Overview | ToC

Meaning: highest score of a SF1 positon weight matrix trained with human data in the last 150 nt 3 prime intron positons of down-stream intron

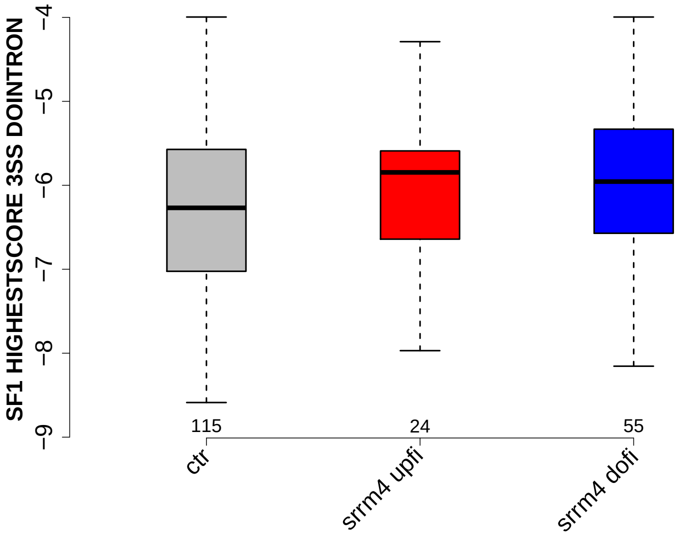

Significant results from Mann-Whitney U test:

- none

6.21 UP 5SS 20INT10EX GCC

Back to: Overview | ToC

Meaning: GC content of up-stream 5ss sequence (20int+10ex positions)

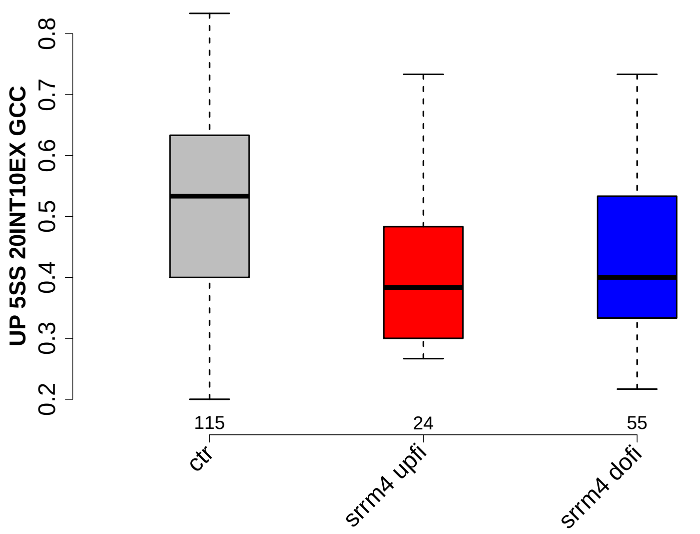

Significant results from Mann-Whitney U test:

- ctr vs srrm4_upfi : 0.00276321
  mean: 0.521884 > 0.425 , median: 0.533333 > 0.383333
- ctr vs srrm4_dofi : 0.000410572
  mean: 0.521884 > 0.433636 , median: 0.533333 > 0.4

6.22 GCC 3SS 20INT10EX

Back to: Overview | ToC

Meaning: GC content of 3ss sequence (20int+10ex positions)

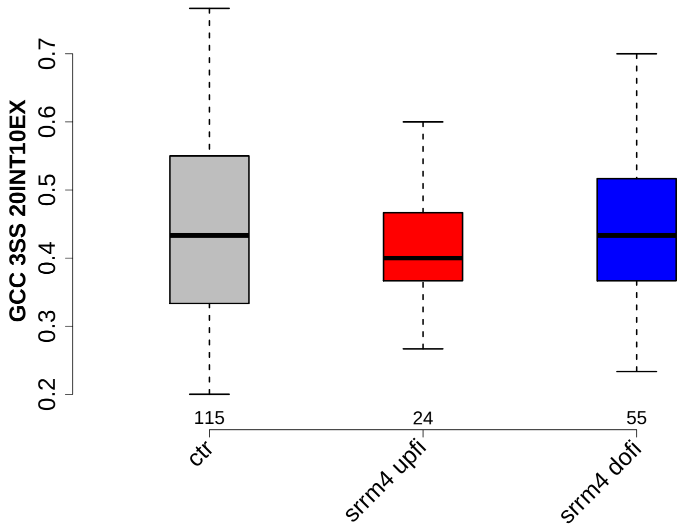

Significant results from Mann-Whitney U test:

- none

6.23 GCC 5SS 20INT10EX

Back to: Overview | ToC

Meaning: GC content of 5ss sequence (20int+10ex positions)

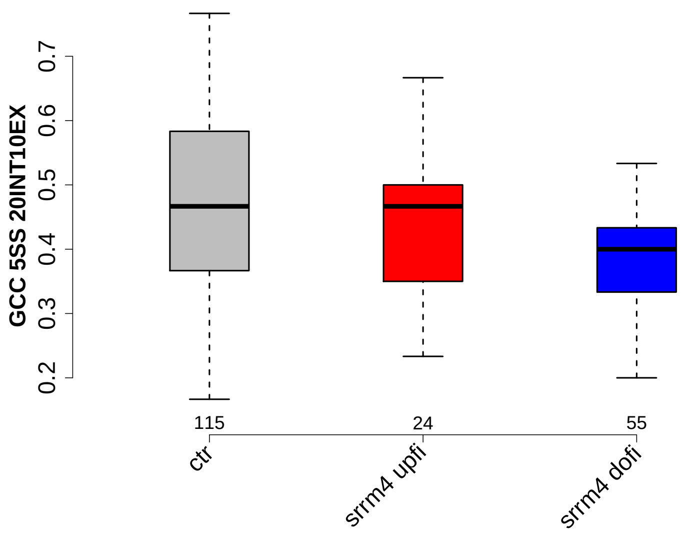

Significant results from Mann-Whitney U test:

- `ctr` vs `srrm4_dofi` : 0.000680785
  mean: 0.469855 > 0.39697 , median: 0.466667 > 0.4

6.24 DO 3SS 20INT10EX GCC

Back to: Overview | ToC

Meaning: GC content of down-stream 3ss sequence (20int+10ex positions)

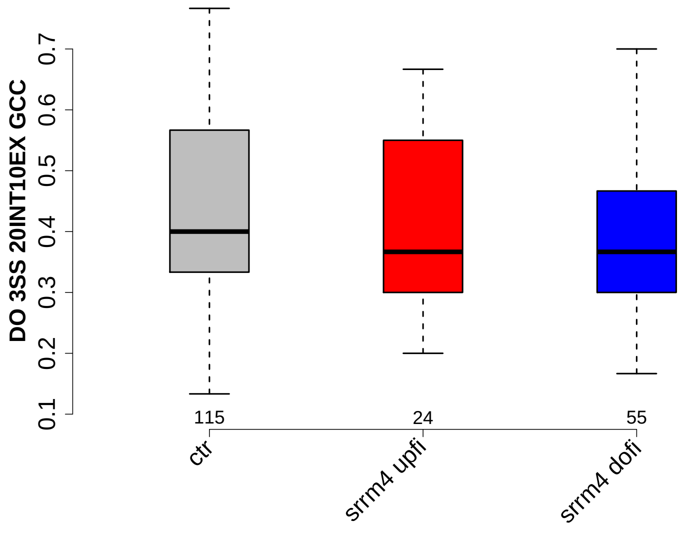

Significant results from Mann-Whitney U test:

- none

6.25 MAXENTSCR HSAMODEL UPSTRM 5SS

Back to: Overview | ToC

Meaning: maximum entropy score of 5ss of up-stream exon using a model trained with human splice sites

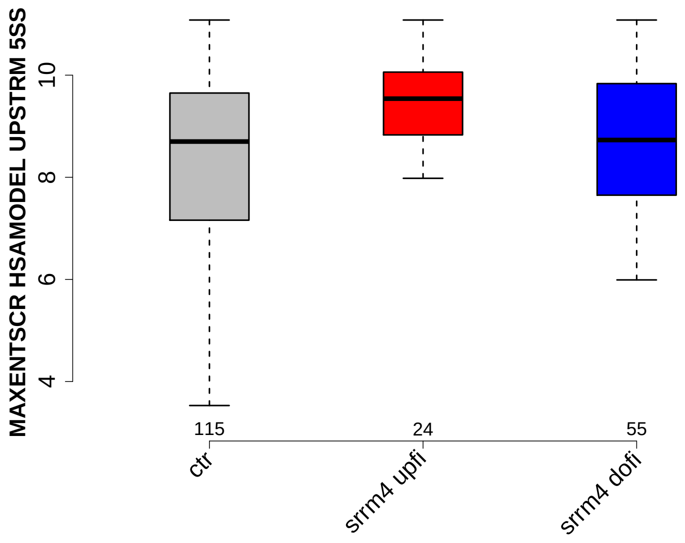

Significant results from Mann-Whitney U test:

- `ctr` vs `srrm4_upfi` : 0.00947595
  mean: 7.734 < 9.0471 , median: 8.7 < 9.54

6.26 MAXENTSCR HSAMODEL 3SS

Back to: Overview | ToC

Meaning: maximum entropy score of 3ss using a model trained with human splice sites

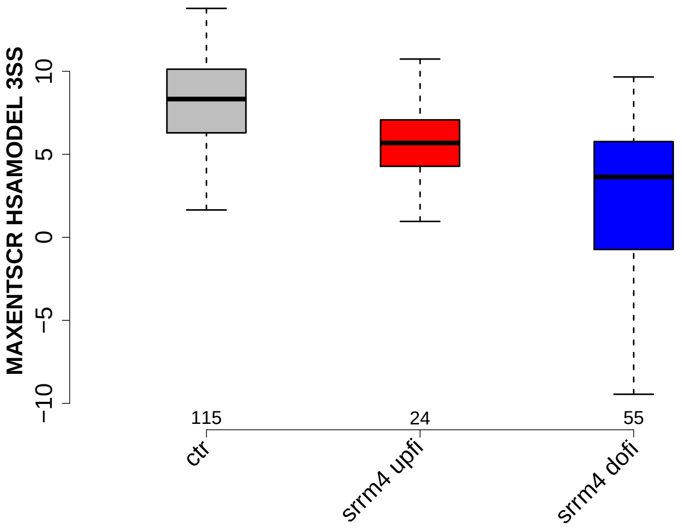

Significant results from Mann-Whitney U test:

- `ctr` vs `srrm4_upfi` : 0.000648238
  mean: 7.2993 > 4.3788 , median: 8.33 > 5.685
- `ctr` vs `srrm4_dofi` : 2.03641e-13
  mean: 7.2993 > 1.7489 , median: 8.33 > 3.64
- `srrm4_upfi` vs `srrm4_dofi` : 0.0103551
  mean: 4.3788 > 1.7489 , median: 5.685 > 3.64

6.27 MAXENTSCR HSAMODEL 5SS

Back to: Overview | ToC

Meaning: maximum entropy score of 5ss using a model trained with human splice sites

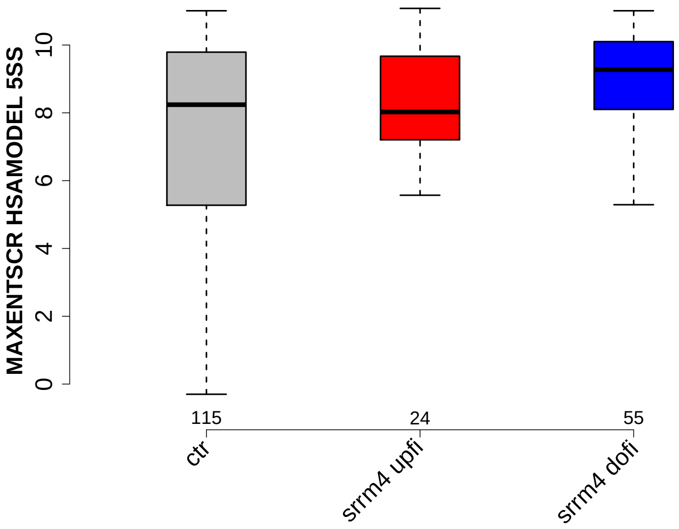

Significant results from Mann-Whitney U test:

- `ctr` vs `srrm4_dofi` : 0.00475608
  mean: 6.5228 < 8.834 , median: 8.24 < 9.27

6.28 MAXENTSCR HSAMODEL DOWNSTRM 3SS

Back to: Overview | ToC

Meaning: maximum entropy score of 3ss of down-stream exon using a model trained with human splice sites

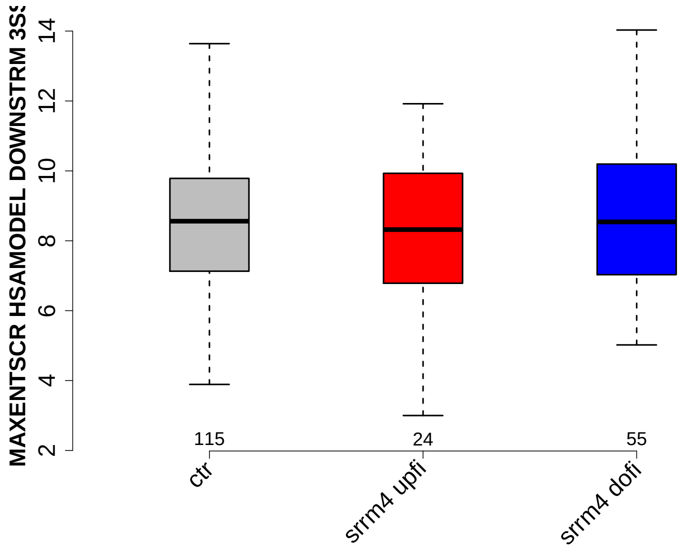

Significant results from Mann-Whitney U test:
- none

6.29 DIST FROM MAXBP TO 3SS UPINTRON

Back to: Overview | ToC

Meaning: distance to 3ss of best precited BP

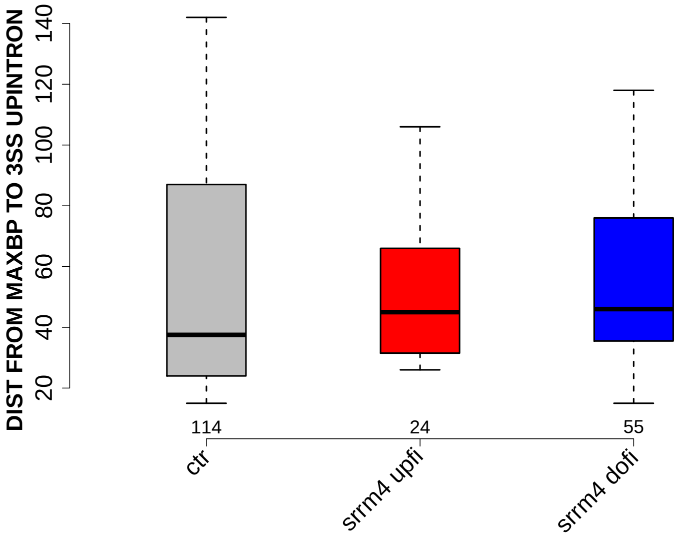

Significant results from Mann-Whitney U test:

- none

6.30 SCORE FOR MAXBP SEQ UPINTRON

Back to: Overview | ToC

Meaning: BP sequence score of best predicted BP

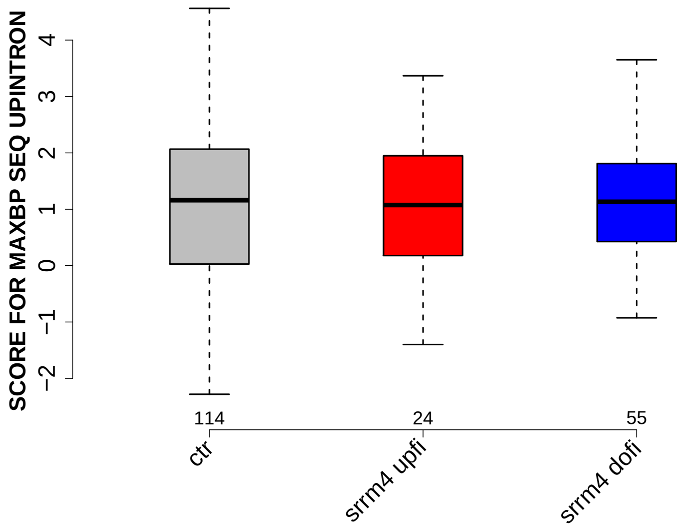

Significant results from Mann-Whitney U test:

- none

6.31 PYRIMIDINECONT MAXBP UPINTRON

Back to: Overview | ToC

Meaning: Pyrimidine content between the BP adenine and the 3 prime splice site for best BP

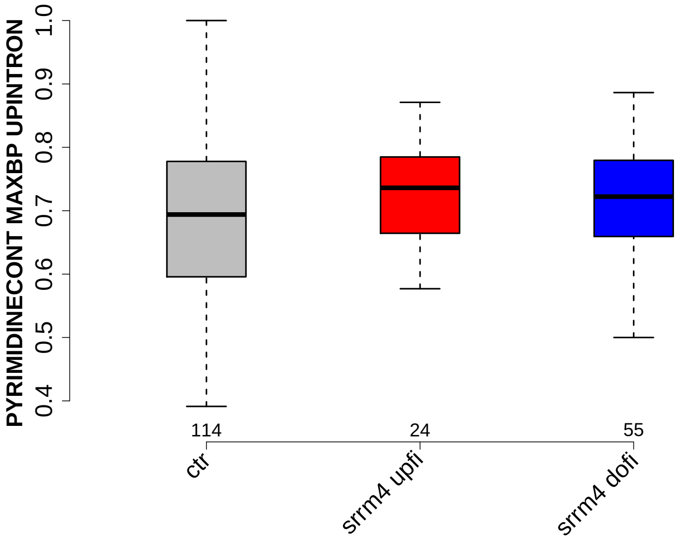

Significant results from Mann-Whitney U test:

- none

6.32 POLYPYRITRAC OFFSET MAXBP UPINTRON

Back to: Overview | ToC

Meaning: Polypyrimidine track offset relative to the BP adenine for best BP

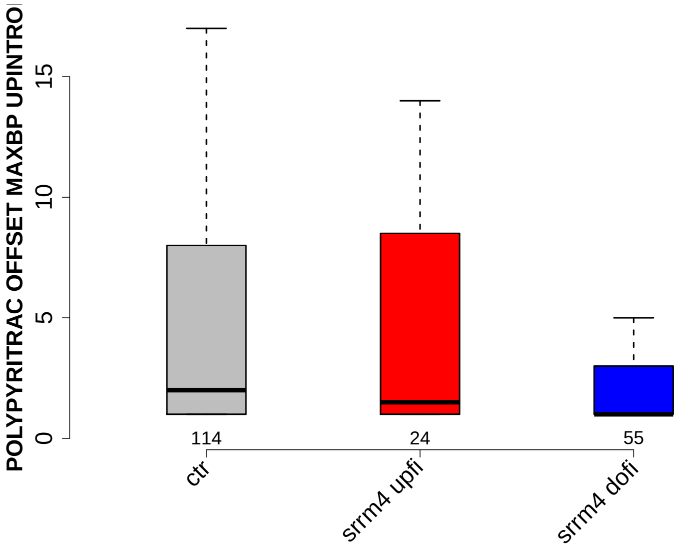

Significant results from Mann-Whitney U test:

- `ctr` vs `srrm4_dofi` : 0.0128239
  mean: 5.2807 > 3.0364 , median: 2 > 1

6.33 POLYPYRITRAC LEN MAXBP UPINTRON

Back to: Overview | ToC

Meaning: Polypyrimidine track length for best BP

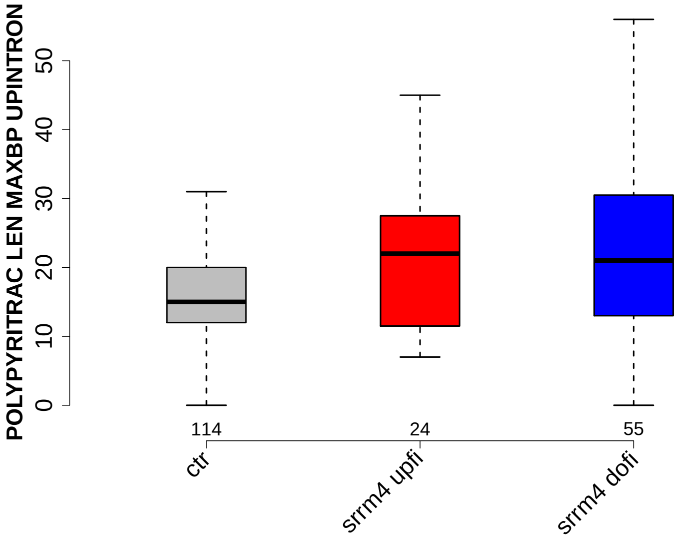

Significant results from Mann-Whitney U test:

- `ctr` vs `srrm4_upfi` : 0.0476083
  mean: 16.2193 < 21.7083 , median: 15 < 22
- `ctr` vs `srrm4_dofi` : 0.00348289
  mean: 16.2193 < 22.0182 , median: 15 < 21

6.34 POLYPYRITRAC SCORE MAXBP UPINTRON

Back to: Overview | ToC

Meaning: Polypyrimidine track score for best BP

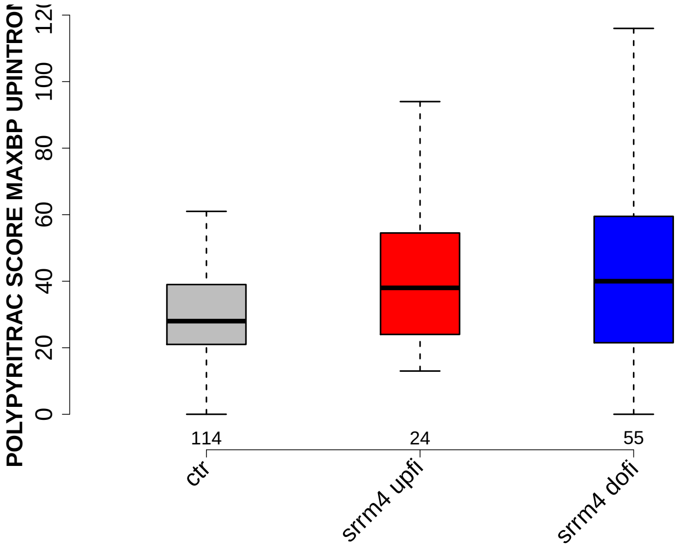

Significant results from Mann-Whitney U test:

- `ctr` vs `srrm4_upfi` : 0.0205878
  mean: 30.6667 < 43.8333 , median: 28 < 38
- `ctr` vs `srrm4_dofi` : 0.0036507
  mean: 30.6667 < 42.6 , median: 28 < 40

6.35 BPSCORE MAXBP UPINTRON

Back to: Overview | ToC

Meaning: SVM classification score of best BP

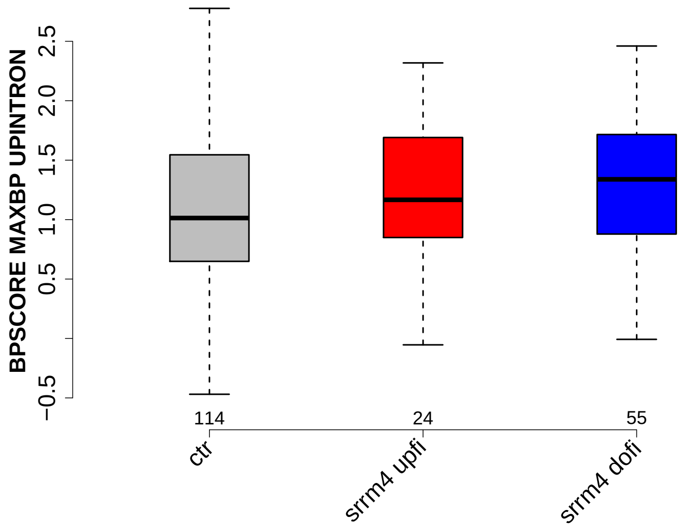

Significant results from Mann-Whitney U test:

- `ctr` vs `srrm4_dofi` : 0.020881
  mean: 1.0135 < 1.2932 , median: 1.0134 < 1.3389

6.36 NUM PREDICTED BPS UPINTRON

Back to: Overview | ToC

Meaning: number of all predicted BPs which have a positive BP score

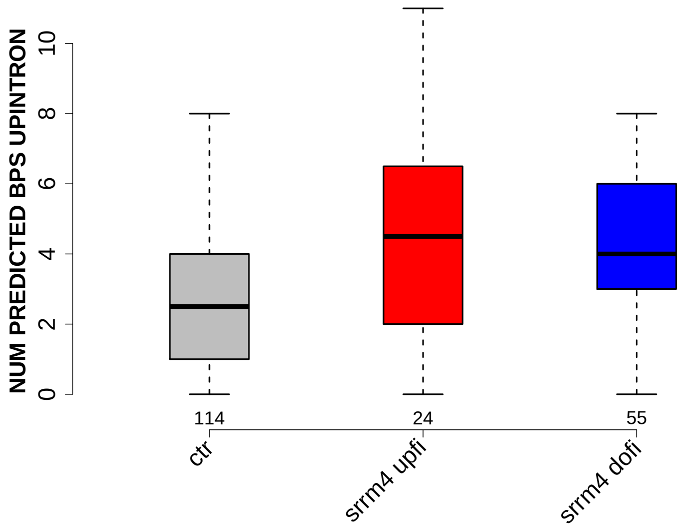

Significant results from Mann-Whitney U test:

- `ctr` vs `srrm4_upfi` : 0.00736109
  mean: 2.9474 < 4.625 , median: 2.5 < 4.5
- `ctr` vs `srrm4_dofi` : 0.000610198
  mean: 2.9474 < 4.0909 , median: 2.5 < 4

6.37 MEDIAN DIST FROM BP TO 3SS UPINTRON

Back to: Overview | ToC

Meaning: like DIST FROM MAXBP TO 3SS but median over top-3 predicted BPs

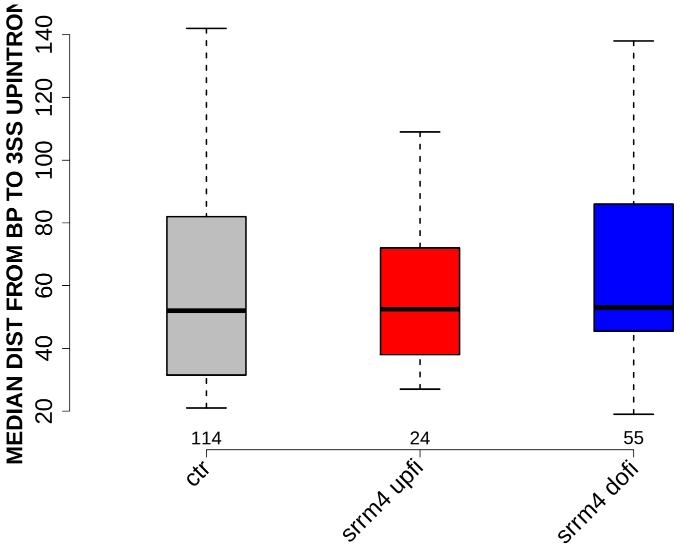

Significant results from Mann-Whitney U test:

- none

6.38 MEDIAN SCORE FOR BPSEQ UPINTRON

Back to: Overview | ToC

Meaning: like SCORE FOR MAXBP SEQ but median over top-3 predicted BPs

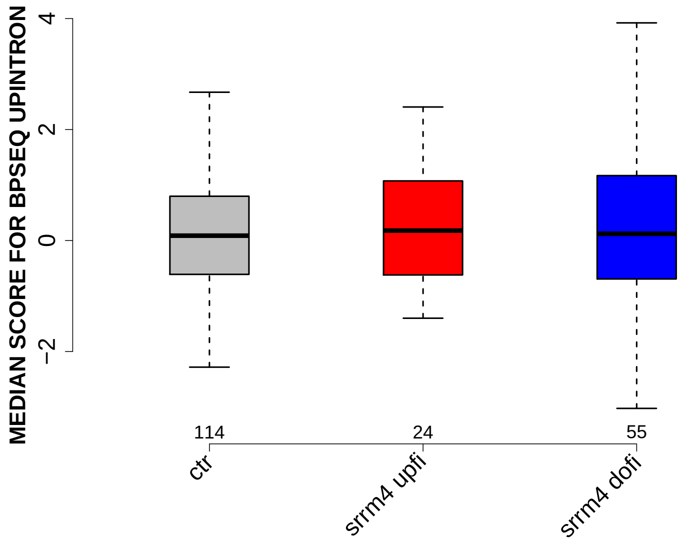

Significant results from Mann-Whitney U test:
- none

6.39 MEDIAN PYRIMIDINECONT UPINTRON

Back to: Overview | ToC

Meaning: like PYRIMIDINECONT MAXBP but median over top-3 predicted BPs

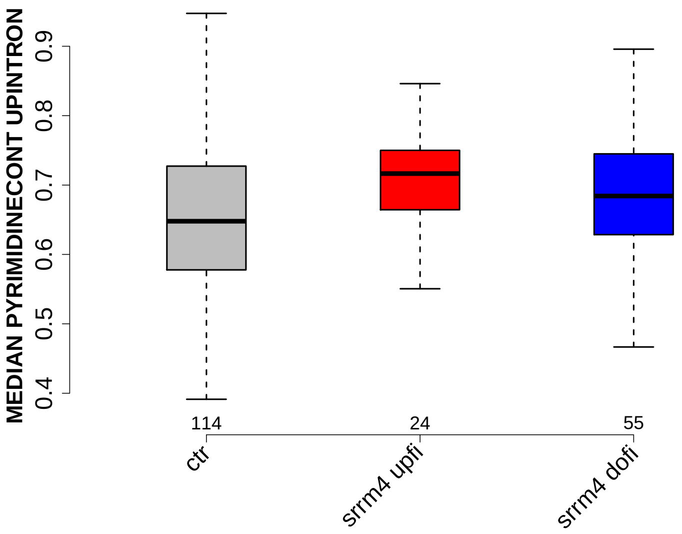

Significant results from Mann-Whitney U test:

- `ctr` vs `srrm4_upfi` : 0.0102522
  mean: 0.653347 < 0.707328 , median: 0.647854 < 0.716518

6.40 MEDIAN POLYPYRITRAC OFFSET UPINTRON

Back to: Overview | ToC

Meaning: like POLYPYRITRAC OFFSET MAXBP but median over top-3 predicted BPs

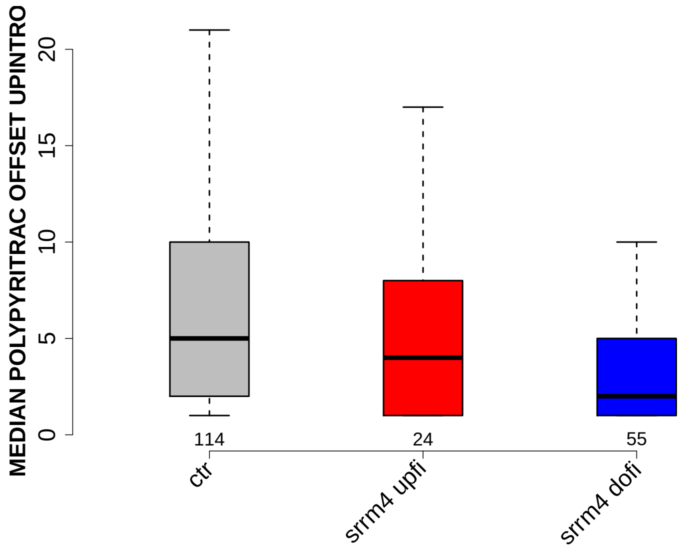

Significant results from Mann-Whitney U test:

- `ctr` vs `srrm4_dofi` : 0.000455268
  mean: 9.1404 > 4.0182 , median: 5 > 2

6.41 MEDIAN POLYPYRITRAC LEN UPINTRON

Back to: Overview | ToC

Meaning: like POLYPYRITRAC LEN MAXBP but median over top-3 predicted BPs

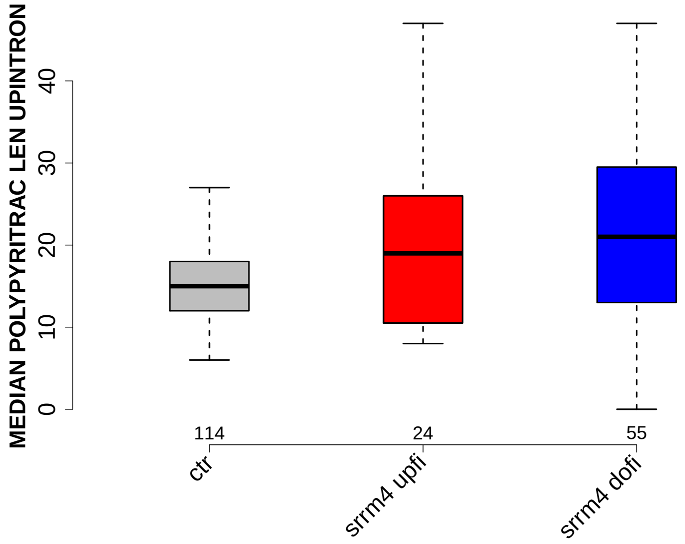

Significant results from Mann-Whitney U test:

- `ctr` vs `srrm4_dofi` : 0.000859434
  mean: 15.8772 < 21.9273 , median: 15 < 21

6.42 MEDIAN POLYPYRITRAC SCORE UPINTRON

Back to: Overview | ToC

Meaning: like POLYPYRITRAC SCORE MAXBP but median over top-3 predicted BPs

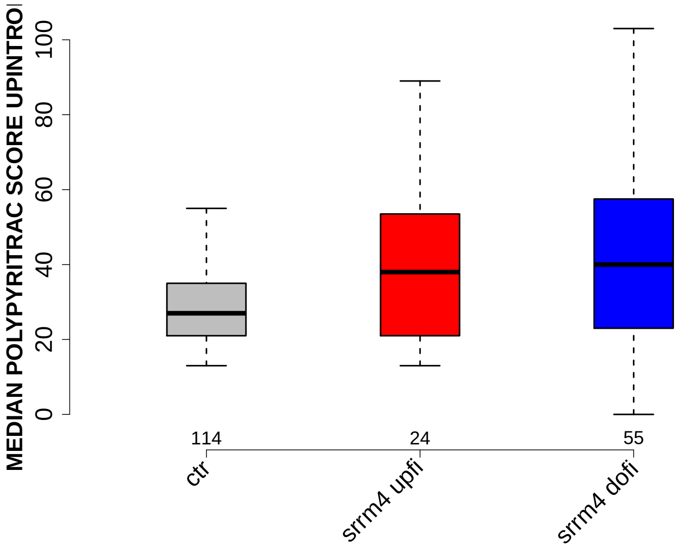

Significant results from Mann-Whitney U test:

- `ctr` vs `srrm4_dofi` : 0.000198875
  mean: 29.5702 < 42.5091 , median: 27 < 40

6.43 MEDIAN BPSCORE UPINTRON

Back to: Overview | ToC

Meaning: like BPSCORE MAXBP but median over top-3 predicted BPs

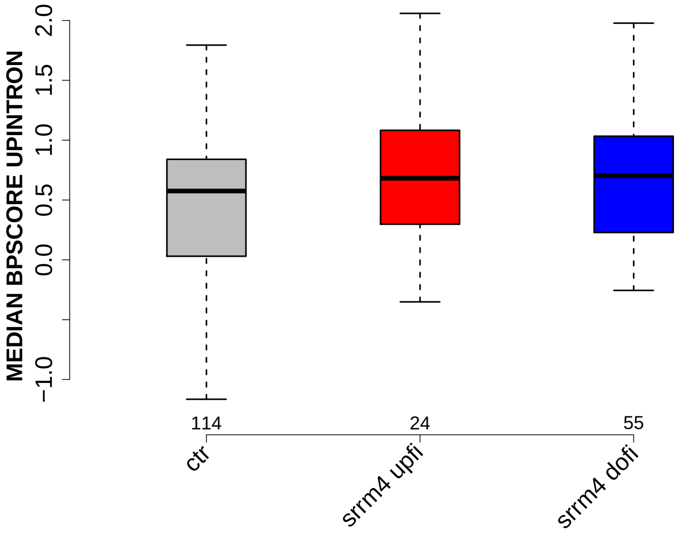

Significant results from Mann-Whitney U test:

- `ctr` vs `srrm4_dofi` : 0.0288168
  mean: 0.303545 < 0.680841 , median: 0.575111 < 0.703222

6.44 DIST FROM MAXBP TO 3SS DOINTRON

Back to: Overview | ToC

Meaning: distance to 3ss of best precited BP

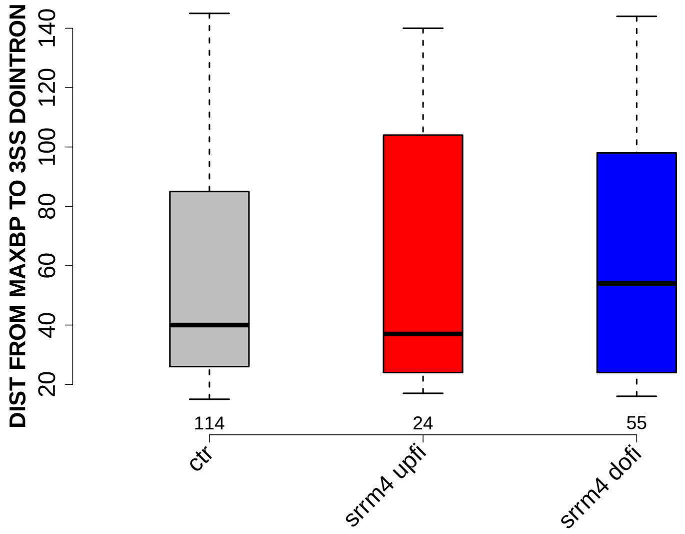

Significant results from Mann-Whitney U test:

- none

6.45 SCORE FOR MAXBP SEQ DOINTRON

Back to: Overview | ToC

Meaning: BP sequence score of best predicted BP

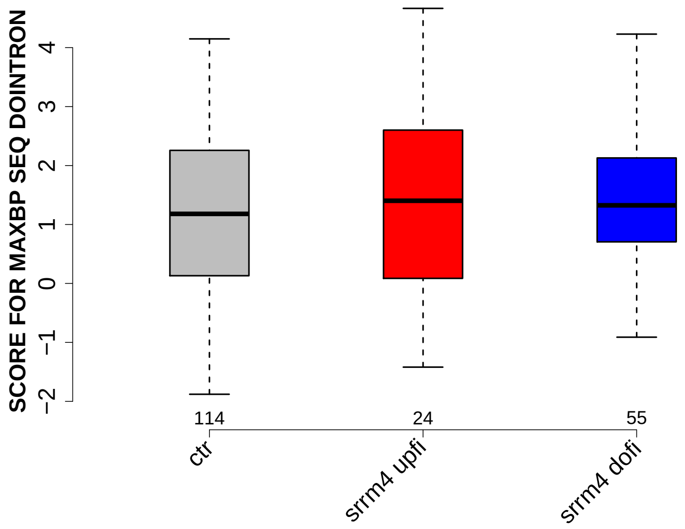

Significant results from Mann-Whitney U test:

- none

6.46 PYRIMIDINECONT MAXBP DOINTRON

Back to: Overview | ToC

Meaning: Pyrimidine content between the BP adenine and the 3 prime splice site for best BP

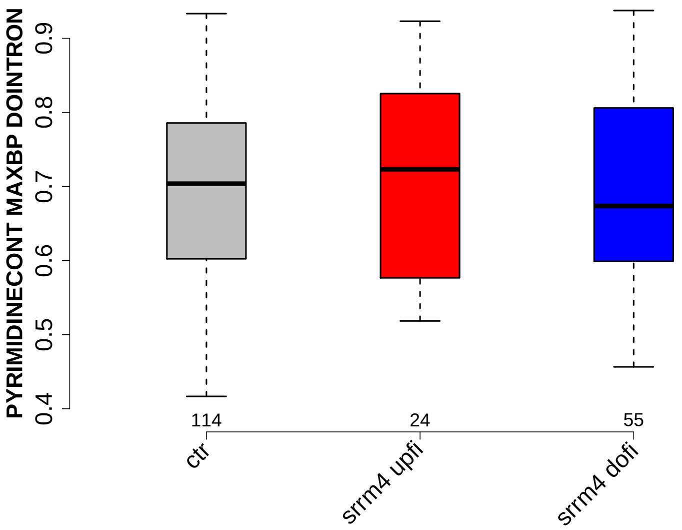

Significant results from Mann-Whitney U test:

- none

6.47 POLYPYRITRAC OFFSET MAXBP DOINTRON

Back to: Overview | ToC

Meaning: Polypyrimidine track offset relative to the BP adenine for best BP

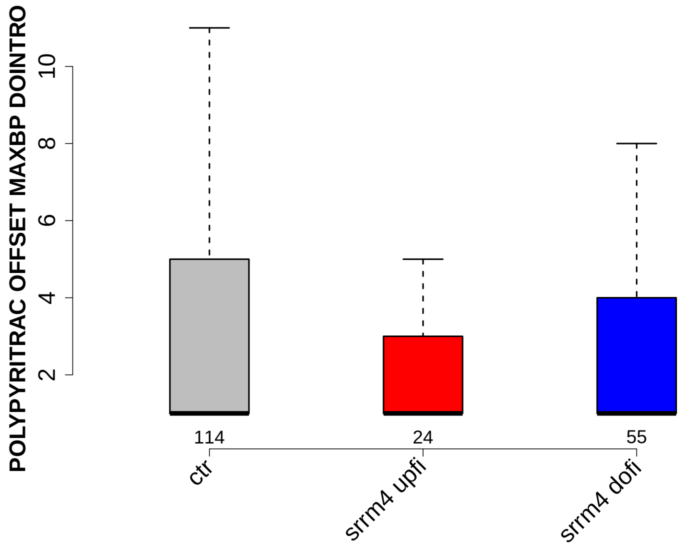

Significant results from Mann-Whitney U test:

- none

6.48 POLYPYRITRAC LEN MAXBP DOINTRON

Back to: Overview | ToC

Meaning: Polypyrimidine track length for best BP

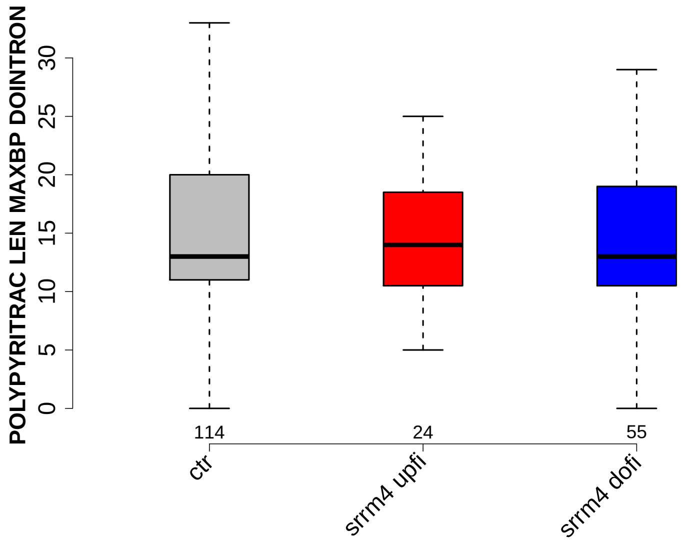

Significant results from Mann-Whitney U test:

- none

6.49 POLYPYRITRAC SCORE MAXBP DOINTRON

Back to: Overview | ToC

Meaning: Polypyrimidine track score for best BP

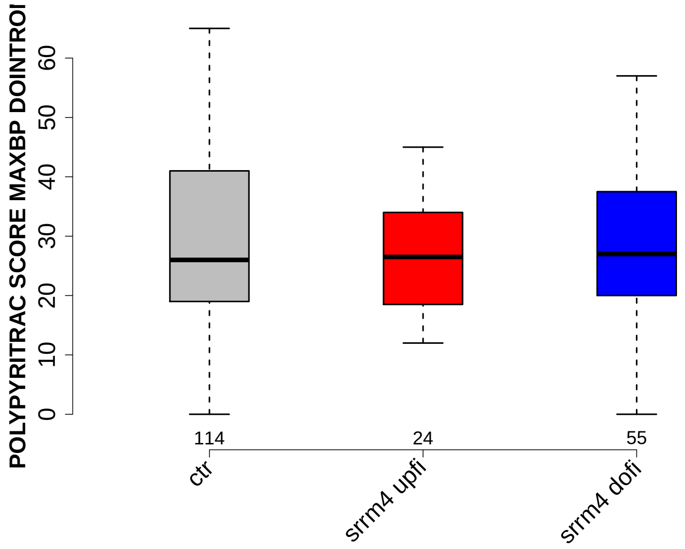

Significant results from Mann-Whitney U test:

- none

6.50 BPSCORE MAXBP DOINTRON

Back to: Overview | ToC

Meaning: SVM classification score of best BP

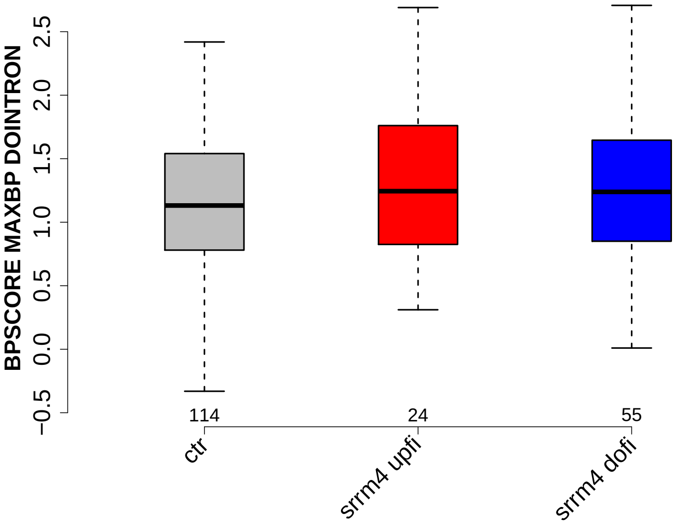

Significant results from Mann-Whitney U test:

- none

6.51 NUM PREDICTED BPS DOINTRON

Back to: Overview | ToC

Meaning: number of all predicted BPs which have a positive BP score

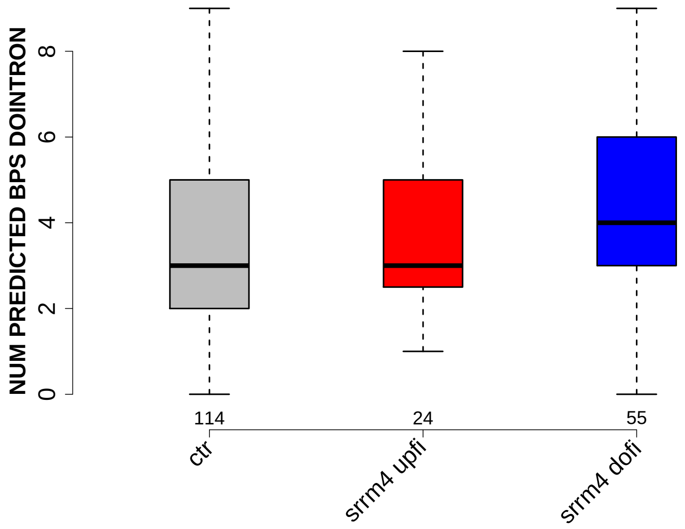

Significant results from Mann-Whitney U test:

- `ctr` vs `srrm4_dofi` : 0.0176817
  mean: 3.4211 < 4.2727 , median: 3 < 4

6.52 MEDIAN DIST FROM BP TO 3SS DOINTRON

Back to: Overview | ToC

Meaning: like DIST FROM MAXBP TO 3SS but median over top-3 predicted BPs

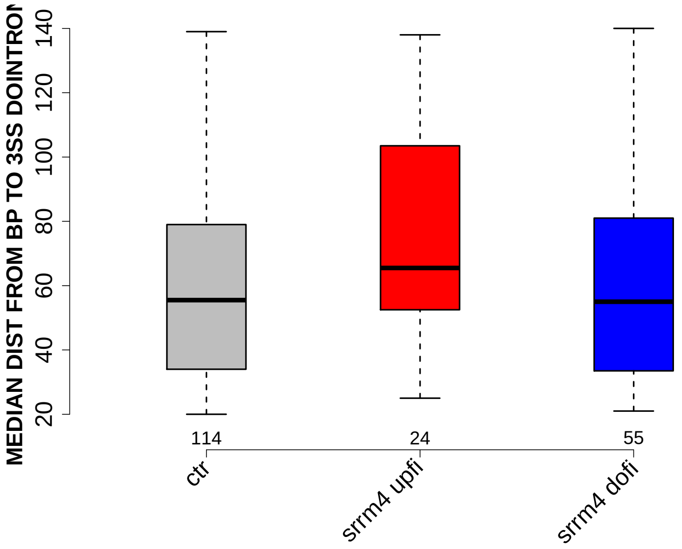

Significant results from Mann-Whitney U test:

- none

6.53 MEDIAN SCORE FOR BPSEQ DOINTRON

Back to: Overview | ToC

Meaning: like SCORE FOR MAXBP SEQ but median over top-3 predicted BPs

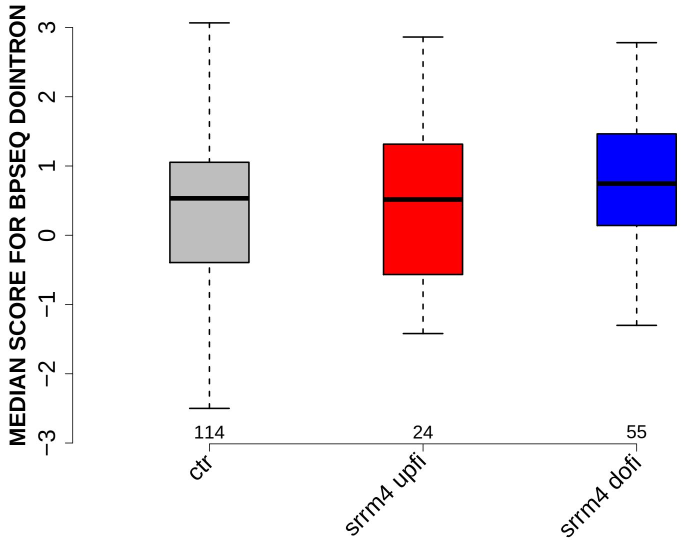

Significant results from Mann-Whitney U test:

- none

6.54 MEDIAN PYRIMIDINECONT DOINTRON

Back to: Overview | ToC

Meaning: like PYRIMIDINECONT MAXBP but median over top-3 predicted BPs

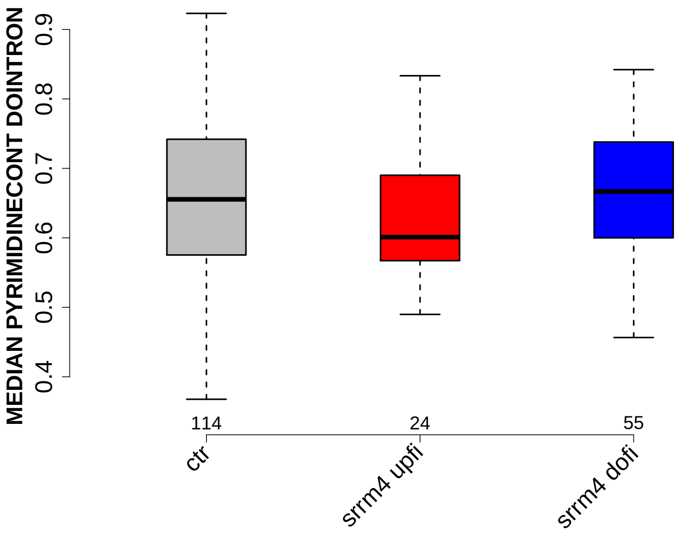

Significant results from Mann-Whitney U test:

- none

6.55 MEDIAN POLYPYRITRAC OFFSET DOINTRON

Back to: Overview | ToC

Meaning: like POLYPYRITRAC OFFSET MAXBP but median over top-3 predicted BPs

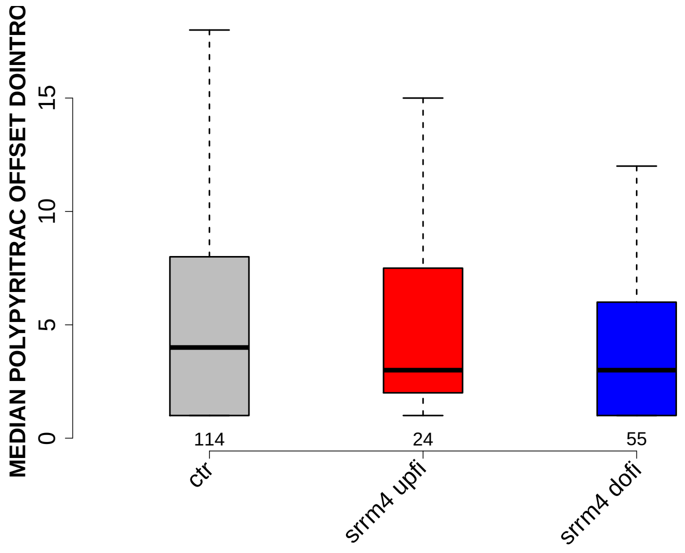

Significant results from Mann-Whitney U test:

- none

6.56 MEDIAN POLYPYRITRAC LEN DOINTRON

Back to: Overview | ToC

Meaning: like POLYPYRITRAC LEN MAXBP but median over top-3 predicted BPs

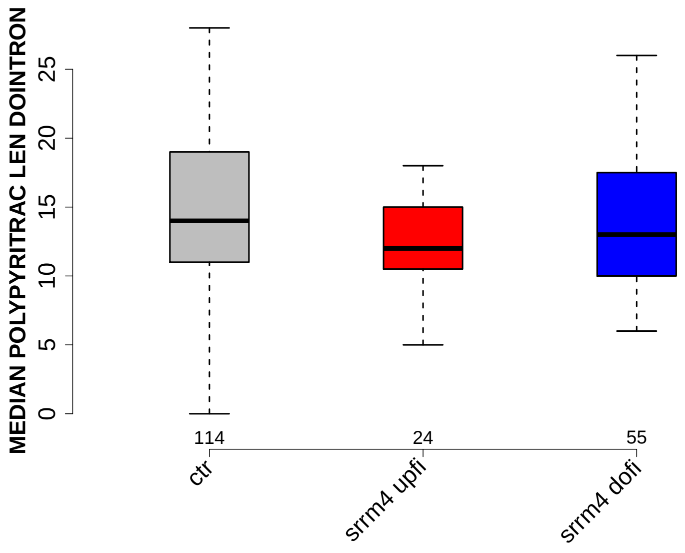

Significant results from Mann-Whitney U test:

- none

6.57 MEDIAN POLYPYRITRAC SCORE DOINTRON

Back to: Overview | ToC

Meaning: like POLYPYRITRAC SCORE MAXBP but median over top-3 predicted BPs

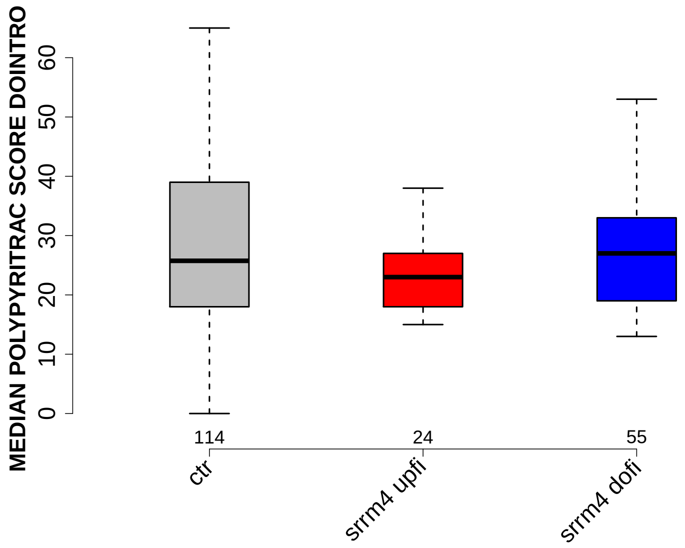

Significant results from Mann-Whitney U test:

- none

6.58 MEDIAN BPSCORE DOINTRON

Back to: Overview | ToC

Meaning: like BPSCORE MAXBP but median over top-3 predicted BPs

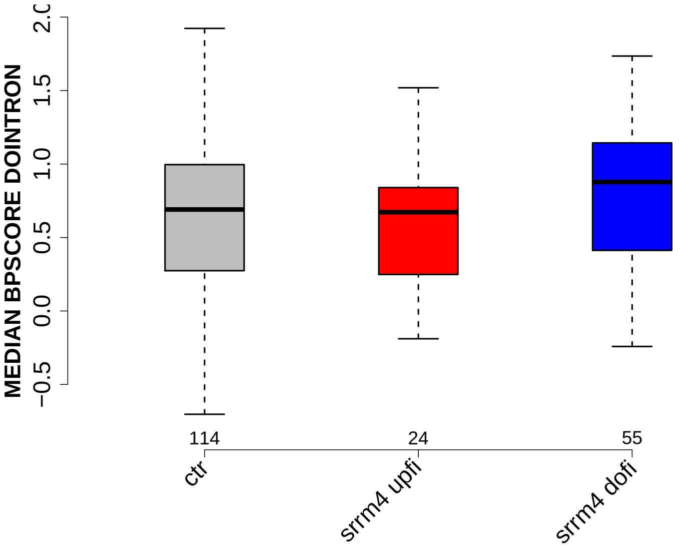

Significant results from Mann-Whitney U test:

- `ctr` vs `srrm4_dofi` : 0.0471817
  mean: 0.464177 < 0.768531 , median: 0.690675 < 0.877449

6.59 MEDIAN TR LENGTH

Back to: Overview | ToC

Meaning: median length of transcripts the exon occurs in

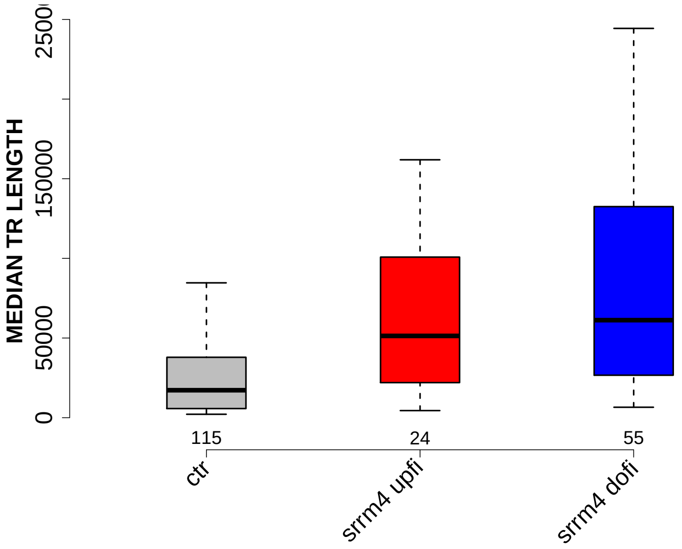

Significant results from Mann-Whitney U test:

- `ctr` vs `srrm4_upfi` : 0.00136682
  mean: 34533.1217 < 85219.8333 , median: 17235 < 51337.75
- `ctr` vs `srrm4_dofi` : 3.80683e-09
  mean: 34533.1217 < 103036.1818 , median: 17235 < 61218

6.60 MEDIAN EXON NUMBER

Back to: Overview | ToC

Meaning: ... of transcripts where exon was found in

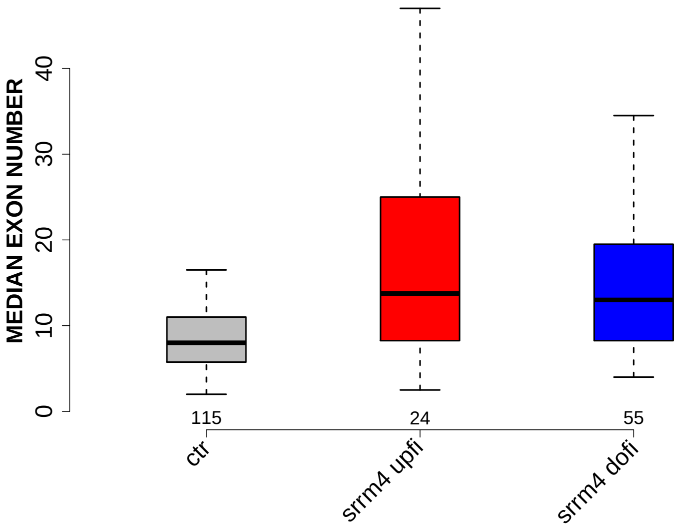

Significant results from Mann-Whitney U test:

- `ctr` vs `srrm4_upfi` : 0.00111351
  mean: 9.5522 < 19.1042 , median: 8 < 13.75
- `ctr` vs `srrm4_dofi` : 1.29223e-05
  mean: 9.5522 < 14.7364 , median: 8 < 13

6.61 EXON MEDIANRELATIVERANK

Back to: Overview | ToC

Meaning: relative rank = rank / number of all exons in transcript, is between 0 and 1

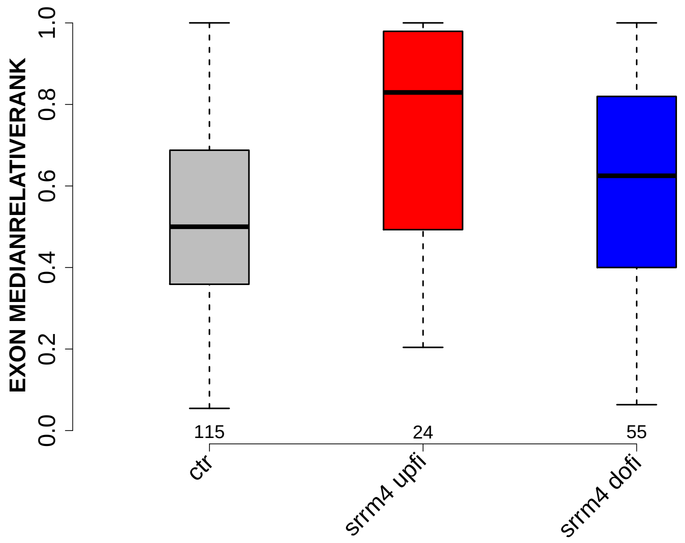

Significant results from Mann-Whitney U test:

- `ctr` vs `srrm4_upfi` : 0.00329206
  mean: 0.531743 < 0.712914 , median: 0.5 < 0.82938

6.62 EXON MEDIANRELATIVERANK 3BINS

Back to: Overview | ToC

Meaning: median bin into which EXON MEDIANRELATIVERANK falls when binning 0-1 into 3 bins

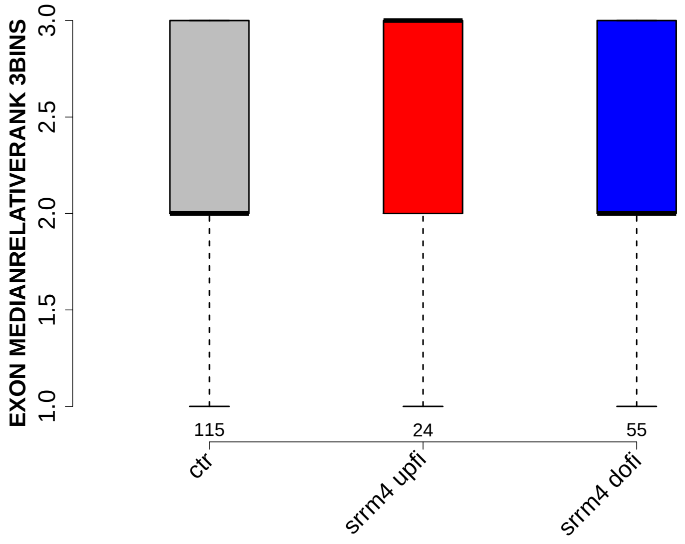

Significant results from Mann-Whitney U test:

- `ctr` vs `srrm4_upfi` : 0.0266448
  mean: 2.1304 < 2.4583 , median: 2 < 3

6.63 EXON MEDIANRELATIVERANK 5BINS

Back to: Overview | ToC

Meaning: similar to EXON MEDIANRELATIVERANK 3BINS with 5 bins

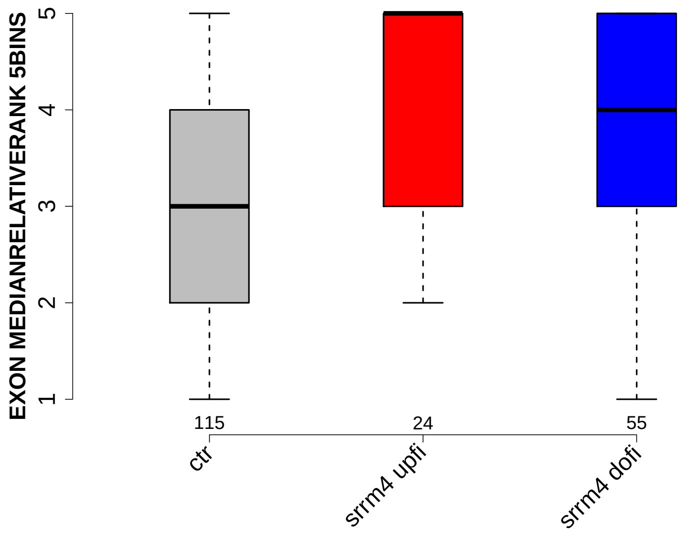

Significant results from Mann-Whitney U test:

- `ctr` vs `srrm4_upfi` : 0.00242098
  mean: 3.1565 $<$ 4 , median: 3 $<$ 5

6.64 EXON MEDIANRELATIVERANK 10BINS

Back to: Overview | ToC

Meaning: similar to EXON MEDIANRELATIVERANK 3BINS with 10 bins

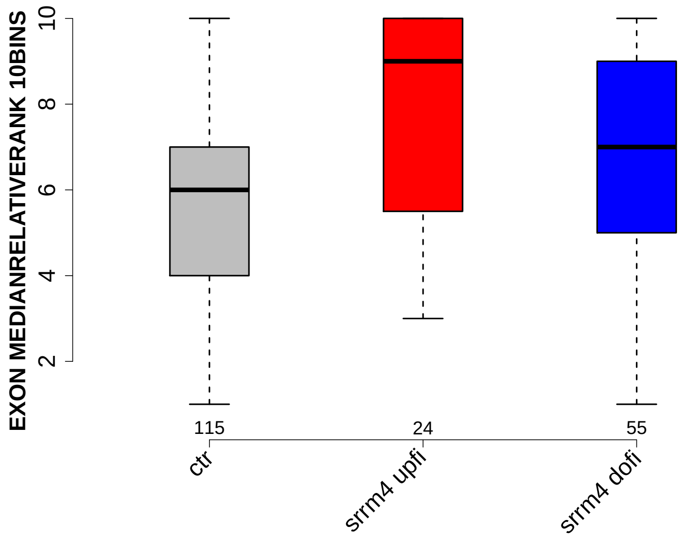

Significant results from Mann-Whitney U test:

- `ctr` vs `srrm4_upfi` : 0.00266538
  mean: 5.8348 < 7.5833 , median: 6 < 9

6.65 NTRS ALL FOR GENE

Back to: Overview | ToC

Meaning: number of transcripts of gene where the exon was found in

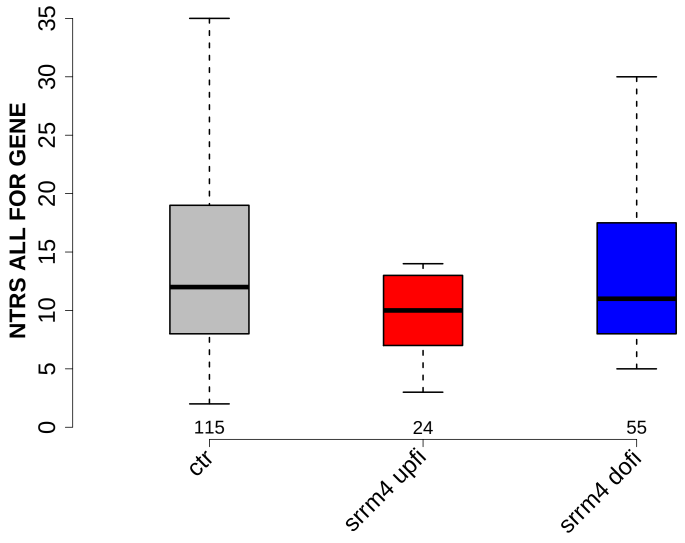

Significant results from Mann-Whitney U test:

- none

6.66 PROP FIRST EXON

Back to: Overview | ToC

Meaning: NTRS WITH EXON AS FIRST EXON / NTRS WITH EXON

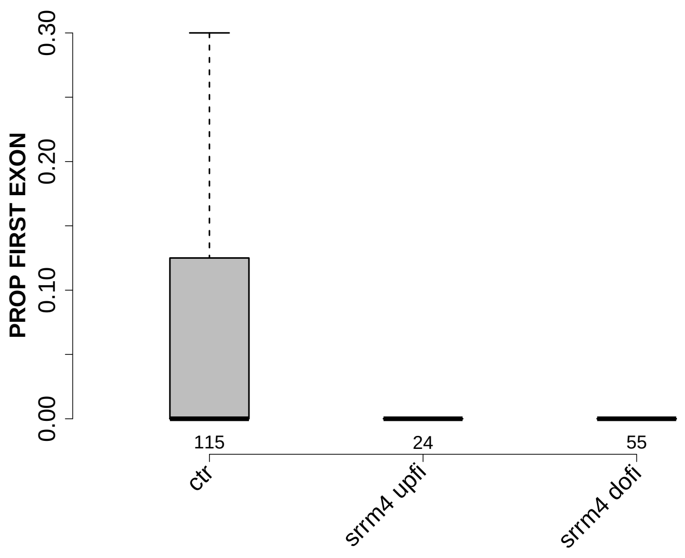

Significant results from Mann-Whitney U test:

- `ctr` vs `srrm4_upfi` : 0.00163247
  mean: 0.0848067 > 0.0104167 , median: 0 = 0
- `ctr` vs `srrm4_dofi` : 0.00587701
  mean: 0.0848067 > 0.0286305 , median: 0 = 0

6.67 PROP LAST EXON

Back to: Overview | ToC

Meaning: NTRS WITH EXON AS LAST EXON / NTRS WITH EXON

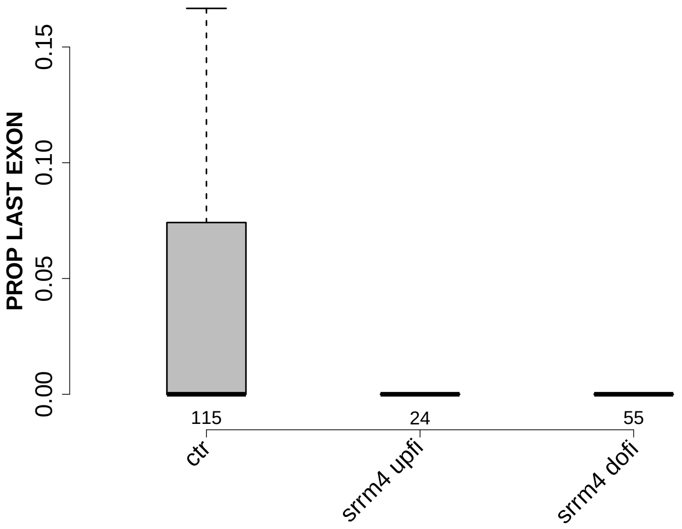

Significant results from Mann-Whitney U test:

- `ctr` vs `srrm4_dofi` : 0.000537025
  mean: 0.0558943 > 0.00854257 , median: 0 = 0

6.68 PROP INTERNAL EXON

Back to: Overview | ToC

Meaning: NTRS WITH EXON AS INTERNAL EXON / NTRS WITH EXON

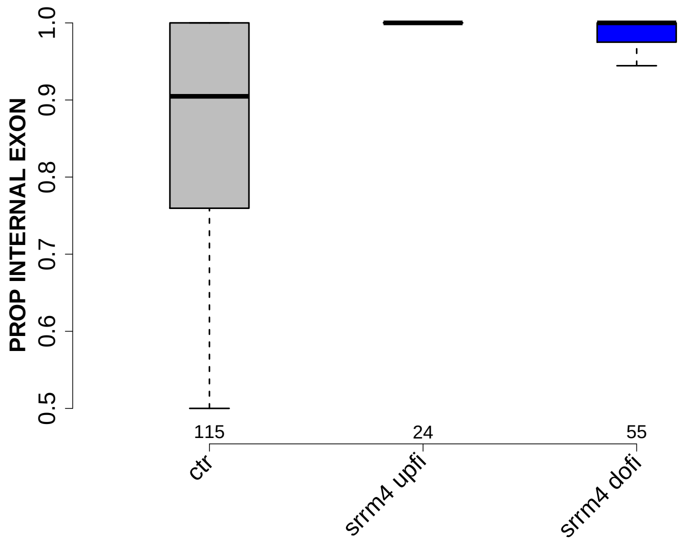

Significant results from Mann-Whitney U test:

- `ctr` vs `srrm4_upfi` : 0.000390521
  mean: 0.860024 $<$ 0.96875 , median: 0.904762 $<$ 1
- `ctr` vs `srrm4_dofi` : 9.09738e-06
  mean: 0.860024 $<$ 0.962827 , median: 0.904762 $<$ 1

6.69 PROP EXON IN UTR

Back to: Overview | ToC

Meaning: NTRS WITH EXON IN UTR / NTRS WITH EXON

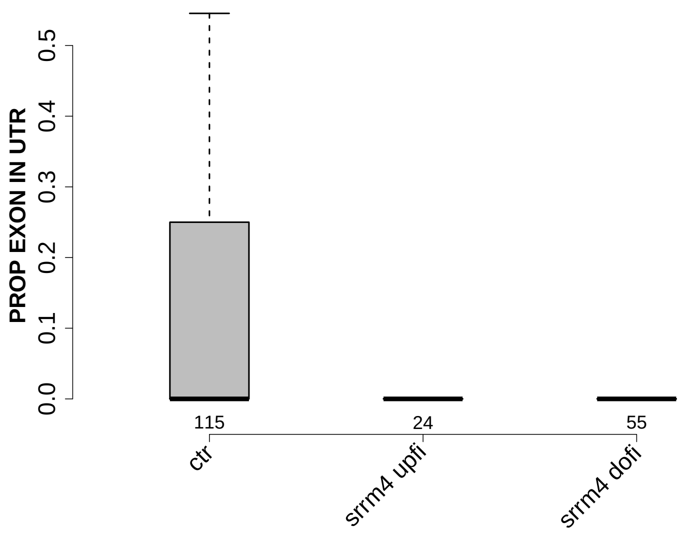

Significant results from Mann-Whitney U test:

- `ctr` vs `srrm4_upfi` : 0.019758
  mean: 0.149222 > 0.0590278 , median: 0 = 0
- `ctr` vs `srrm4_dofi` : 1.06979e-05
  mean: 0.149222 > 0.0251787 , median: 0 = 0
